# Supplementary material for: Regulation of CYLD activity and specificity by phosphorylation and ubiquitin-binding CAP-Gly domains
Source: Cell Rep. 2021 Oct 5;37(1):109777. doi: 10.1016/j.celrep.2021.109777 (PMC8511506; doi:10.1016/j.celrep.2021.109777)
Supplement: Document S1. Table S1, Figures S1–S7, and Data S1–S4 [file mmc1.pdf]

Cell Reports, Volume *37*

Supplemental information

Regulation of CYLD activity and specificity by phosphorylation and ubiquitin-binding CAP-Gly domains

Paul R. Elliott, Derek Leske, Jane Wagstaff, Lisa Schlicher, Georgina Berridge, Sarah Maslen, Frederik Timmermann, Biao Ma, Roman Fischer, Stefan M.V. Freund, David Komander, and Mads Gyrd-Hansen

Table S1. Data Collection and Refinement Statistics, Related to Figure 2.

	CYLD 464-552 + Ub	CYLD 464-565 + Ub
**Data collection**		
Beamline	Diamond I03	Diamond I03
Space group	$P\ 4_1 2_1 2$	$P\ 2_1 2_1 2_1$
a, b, c (Å)	43.93, 42.93, 171.34	63.93, 64.49, 75.93
α, β, γ (°)	90.00, 90.00, 90.00	90.00, 90.00, 90.00
Wavelength	0.96863	0.96863
Resolution (Å)	41.64-1.71 (1.74-1.71)	64.49-1.85 (1.89-1.85)
R_{merge}	4.2 (59.1)	6.1 (56.6)
$< I / \sigma I >$	15.0 (1.9)	14.0 (2.1)
CC(1/2)	0.99 (0.90)	0.99 (0.75)
Completeness (%)	100.0 (99.9)	100.0 (100.0)
Redundancy	6.0 (6.3)	6.3 (6.3)
**Refinement**		
Resolution (Å)	41.64 - 1.71	49.15 - 1.85
No. reflections (test set)	18,237	27,435
R_{work} / R_{free}	21.0/23.8	18.3/23.6
No. atoms		
Protein	1342	2658
Water	98	192
B-factors		
Wilson B	34.76	29.47
Protein	49.9	40.6
Water	55.1	46.4
R.m.s deviations		
Bond lengths (Å)	0.011	0.009
Bond angles (°)	1.174	1.017
Ramachandran statistics (outliers, allowed, favoured)	0.6, 3.8, 95.6	0.0, 1.5, 98.5

Numbers in brackets are for the highest resolution bin.
Datasets were collected and structures determined from a single crystal each.

Figure S1

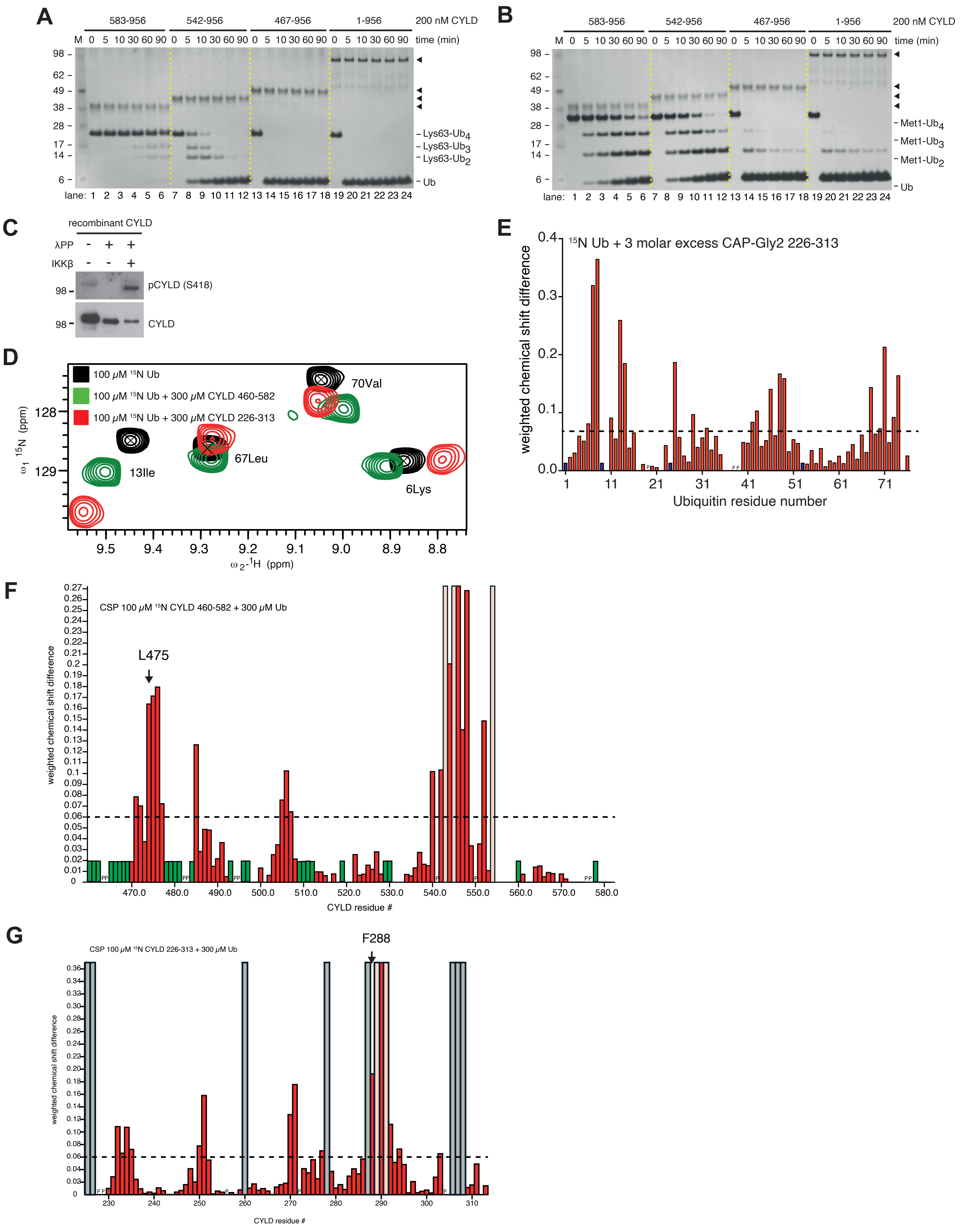

A
583-956
542-956
467-956
1-956
200 nM CYLD
time (min)
Lys63-Ub4
Lys63-Ub3
Lys63-Ub2
Ub
lane:
B
Met1-Ub4
Met1-Ub3
Met1-Ub2
C
recombinant CYLD
λPP
IKKβ
pCYLD (S418)
CYLD
D
100 μM 15N Ub
100 μM 15N Ub + 300 μM CYLD 460-582
100 μM 15N Ub + 300 μM CYLD 226-313
70Val
67Leu
13Ile
6Lys
ω1 15N (ppm)
ω2-1H (ppm)
E
15N Ub + 3 molar excess CAP-Gly2 226-313
weighted chemical shift difference
Ubiquitin residue number
F
CSP 100 μM 15N CYLD 460-582 + 300 μM Ub
L475
weighted chemical shift difference
CYLD residue #
G
CSP 100 μM 15N CYLD 226-313 + 300 μM Ub
F288
weighted chemical shift difference
CYLD residue #

Figure S1: IKKβ phosphorylates CYLD *in vitro*, Related to Figure 1.

(A) Qualitative DUB assays for assessing CYLD enzyme activity. Lys63-Ub4 was incubated with different CYLD fragments (200 nM) and Ub cleavage was monitored over 90 minutes. Samples were resolved by SDS-PAGE and stained using silver stain. (B) Qualitative DUB assays as in (A) but using Met1 Ub4 as a substrate. (C) To investigate phosphorylation of CYLD by IKKβ, full-length CYLD purified from sf9 cells was treated with λPP. The λPP was subsequently removed by size exclusion chromatography and the dephosphorylated CYLD was either incubated with buffer alone (-) or IKKβ. Phosphorylation status of CYLD was determined by western blotting as indicated. (D) BEST-TROSY region for ^{15}N-labelled Ub (**Extended Data 1**) collected alone or in the presence of three-molar excess CAP-Gly2 (226-313) or CAP-Gly3 (460-582). (E) Chemical shift perturbation plot of ^{15}N-labelled Ub titrated with three-fold excess of CAP-Gly2 (226-313). (F) Chemical shift perturbation plot of ^{15}N-labelled CAP-Gly3 titrated with three-fold excess of Ub. (G) Chemical shift perturbation plot as in (F) with ^{15}N-labelled CAP-Gly2.

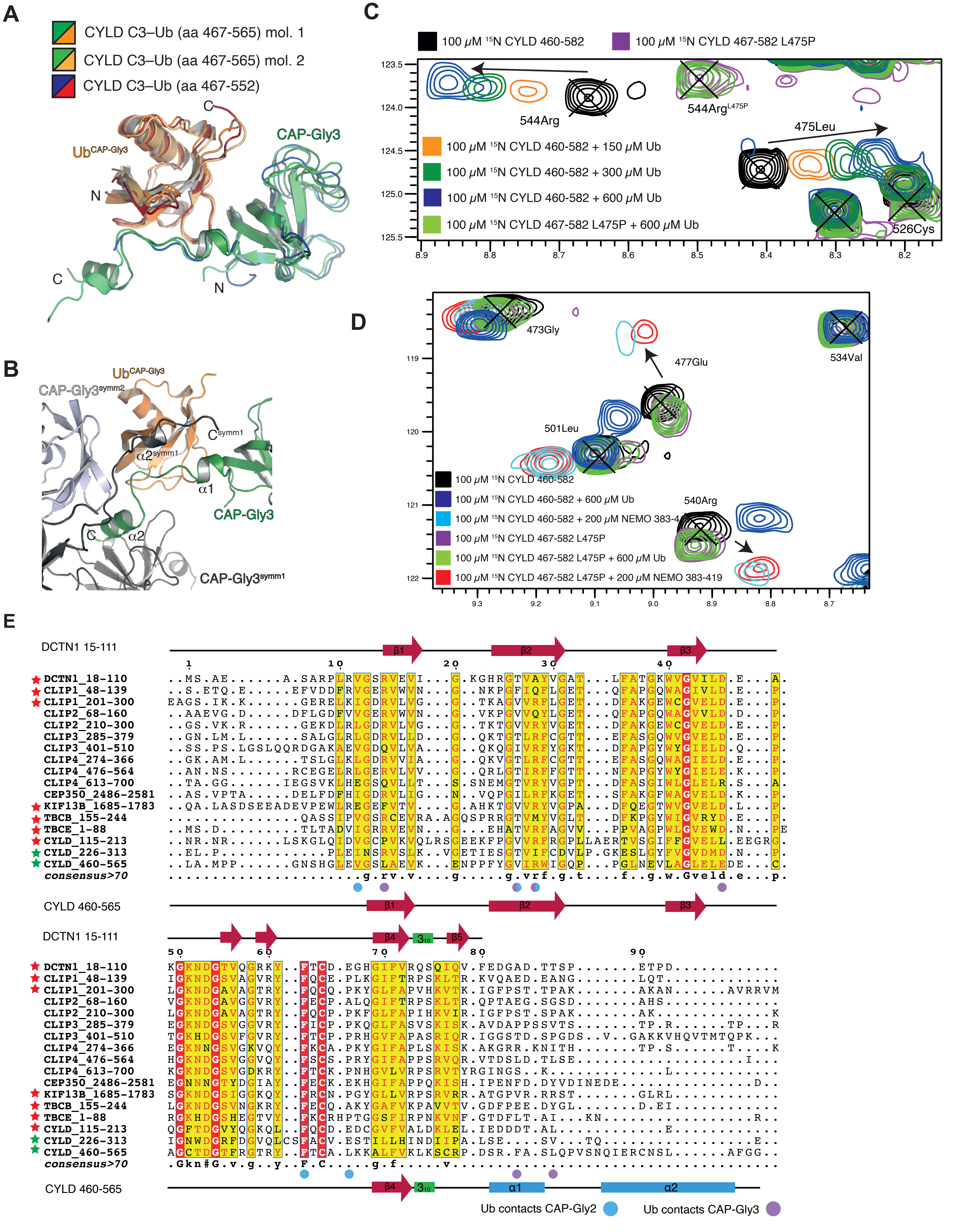
A
CYLD C3–Ub (aa 467-565) mol. 1
CYLD C3–Ub (aa 467-565) mol. 2
CYLD C3–Ub (aa 467-552)
C
Ub^CAP-Gly3
CAP-Gly3
N
C
N
B
Ub^CAP-Gly3
CAP-Gly3^symm2
C^symm1
α2^symm1
α1
CAP-Gly3
C
α2
CAP-Gly3^symm1
C
100 μM ¹⁵N CYLD 460-582
100 μM ¹⁵N CYLD 467-582 L475P
123.5
124.0
124.5
125.0
125.5
544Arg
544Arg^L475P
475Leu
526Cys
100 μM ¹⁵N CYLD 460-582 + 150 μM Ub
100 μM ¹⁵N CYLD 460-582 + 300 μM Ub
100 μM ¹⁵N CYLD 460-582 + 600 μM Ub
100 μM ¹⁵N CYLD 467-582 L475P + 600 μM Ub
8.9
8.8
8.7
8.6
8.5
8.4
8.3
8.2
D
473Gly
534Val
477Glu
501Leu
540Arg
119
120
121
122
100 μM ¹⁵N CYLD 460-582
100 μM ¹⁵N CYLD 460-582 + 600 μM Ub
100 μM ¹⁵N CYLD 460-582 + 200 μM NEMO 383-419
100 μM ¹⁵N CYLD 467-582 L475P
100 μM ¹⁵N CYLD 467-582 L475P + 600 μM Ub
100 μM ¹⁵N CYLD 467-582 L475P + 200 μM NEMO 383-419
9.3
9.2
9.1
9.0
8.9
8.8
8.7
E
DCTN1 15-111
β1
β2
β3
1
10
20
30
40
DCTN1_18-110 ..MS.AE......A.SARPLRVGSRVEVI...G.KGHRGTVAYVGAT...LFATGKWVGVILD.E...A.
CLIP1_48-139 ..S.ETQ.E.....EFVDDFRVGERVWVN...G..NKPGFIQFLGET...QFAPGQWAGIVLD.E...P.
CLIP1_201-300 EAGS.IK.K......GERELKIGDRVLVG...G..TKAGVVRFLGET...DFAKGEWCGVELD.E...P.
CLIP2_68-160 ..AAEVG.D.....DFLGDFVVGERVWVN...G..VKPGVVQYLGET...QFAPGQWAGVVLD.D...P.
CLIP2_210-300 ..GS.VK.R......GEKDLRLGDRVLVG...G..TKTGVVRYVGET...DFAKGEWCGVELD.E...P.
CLIP3_285-379 ..GN.LM.L......SALGLRLGDRVLLD...G..QKTGTLRFCGTT...EFASGQWVGVELD.E...P.
CLIP3_401-510 ..SS.PS.LGSLQQRDGAKAEVGDQVLVA...G..QKQGIVRFYGKT...DFAPGYWYGIELD.Q...P.
CLIP4_274-366 ..GK.AM.L......TSLGLKLGDRVVIA...G..QKVGTLRFCGTT...EFASGQWAGIELD.E...P.
CLIP4_476-564 ..AN.NS......RCEGELRLGERVLVV...G..QRLGTIRFFGTT...NFAPGYWYGIELE.K...P.
CLIP4_613-700 ..TA.GG......IEGSVKLHEGSQVLLT...S.SNEMGTVRYVGPT...DFASGIWLGLELR.S...A.
CEP350_2486-2581 ..AS.VPTA......DELFDFHIGDRVLIG...N..VQPGILRFKGET...SFAKGFWAGVELD.K...P.
KIF13B_1685-1783 ..QA.LASDSEEADEVPEWLREGEFVTV....G.AHKTGVVRYVGPA...DFQEGTWVGVELD.L...P.
TBCB_155-244 ...............QASSIPVGSRCEVRA.AGQSPRRGTVMYVGLT...DFKPGYWIGVRYD.E...P.
TBCE_1-88 ..MS.D.........TLTADVIGRRVEVN....G...EHATVRFAGVV...PPVAGPWLGVEWD.N...PE
CYLD_115-213 ..NR.NR......LSKGLQIDVGCPVKVQLRSGEEKFPGVVRFRGPLLAERTVSGIFFGVELL.EEGRG.
CYLD_226-313 ..EL.P...........PLEINSRVSLK..VGETIESGTVIFCDVLP.GKESLGYFVGVDMD.N...P.
CYLD_460-565 ..LA.MPP......GNSHGLEVGSLAEVK...ENPPFYGVIRWIGQP...PGLNEVLAGLELEDE...C.
consensus>70 ....g.rv.v....g.....g.vrf.g.t....f..g.w.Gveld.e...p.
CYLD 460-565
β1
β2
β3
DCTN1 15-111
β4
3₁₀
β5
50
60
70
80
90
DCTN1_18-110 KGKNDGTVQGRKY..FTCD.EGHGIFVRQSQIQV.FEDGAD.TTSP.......ETPD............
CLIP1_48-139 IGKNDGSVAGVRY..FQCE.PLKGIFTRPSKLTR.KVQAED.EANG.......LQT.............
CLIP1_201-300 LGKNDGAVAGTRY..FQCQ.PKYGLFAPVHKVTK.IGFPST.TPAK.......AKAN.......AVRRVM
CLIP2_68-160 VGKNDGAVGGVRY..FECP.ALQGIFTRPSKLTR.QPTAEG.SGSD.......AHS.............
CLIP2_210-300 LGKNDGAVAGTRY..FQCP.PKFGLFAPIHKVIR.IGFPST.SPAK.......AK.............K
CLIP3_285-379 EGKNDGSVGGVRY..FICP.PKQGLFASVSKISK.AVDAPPSSVTS.......TP.R.......TP...R
CLIP3_401-510 TGKHDGSVFGVRY..FTCP.PRHGVFAPASRIQR.IGGSTD.SPGDS..V..GAKKVHQVTMTQPK...
CLIP4_274-366 EGKNNGSVGKVQY..FKCA.PKYGIFAPLSKISK.AKGRR.KNITH.......TP.S.......T....K
CLIP4_476-564 HGKNDGSVGGVQY..FSCS.PRYGIFAPPSRVQR.VTDSLD.TLSE.....................
CLIP4_613-700 KGKNDGSVGDKRY..FTCK.PNHGVLVRPSRVTYRGINGS..K.............L.......I....
CEP350_2486-2581 EGNNNGTYDGIAY..FECK.EKHGIFAPPQKISH.IPENFD.DYVDINEDE..............D..
KIF13B_1685-1783 SGKNDGSIGGKQY..FRCN.PGYGLLVRPSRVRR.ATGPVR.RRST.......GLRL............
TBCB_155-244 LGKNDGSVNGKRY..FECQ.AKYGAFVKPAVVTV.GDFPEE.DYGL.......D.EI............
TBCE_1-88 RGKHDGSHEGTVY..FKCRHPTGGSFIRPNKVNF.GTDFLT.AI...KN..................R..
CYLD_115-213 QGFTDGVYQGKQL..FQCD.EDCGVFVALDKLEL.IEDDDT.AL.........E...............
CYLD_226-313 IGNWDGRFDGVQLCSFACV.ESTILLHINDIIPA.LSE.....SV...TQ..................E..
CYLD_460-565 ACTDGTFRGTRY..FTCA.LKKALFVKLKSCRP.DSR.FA.SLQPVSNQIERCNSL......AFGG..
consensus>70 .Gkn#G.v.g..y..F.C......g.f......v.....................
CYLD 460-565
β4
3₁₀
α1
α2
Ub contacts CAP-Gly2
Ub contacts CAP-Gly3

Figure S2: CAP-Gly domain 2 and 3 of CYLD bind Ub, Related to Figure 2.

(A) Superimposition of the two CAP-Gly3 molecules from crystal form 2 (467-565) (orthorhombic space group $P2_12_12_1$) and CAP-Gly3 (467-552) from crystal form 1 (tetragonal space group $P4_12_12$) onto one another, revealing low RMSD between the Ub orientation in all the structures obtained. (B) Crystal packing within crystal form 2 stabilizes helix α2 of CAP-Gly3 and positions Val551 against a separate interface of Ub. (C) BEST-TROSY region for ^{15}N-labelled CAP-Gly3 and the CAP-Gly3 Ub-binding mutant, L475P (**Extended Data 3-4).** The L475P mutation does not significantly alter the conformation of the CAP-Gly3 domain and is unable to bind Ub. (D) BEST-TROSY region for ^{15}N-labelled CAP-Gly3 and CAP-Gly3 L475P in the presence of six-fold molar excess of Ub or two-fold molar excess NEMO (383-419), revealing that mutation of the Ub binding interface does not affect NEMO binding. (E) Structure-based sequence alignment of all reported mammalian CAP-Gly domains. Secondary structure for DCTN1 CAP-Gly domain (18-110) is shown on top and CYLD CAP-Gly3 (460-565) is shown on the bottom. CAP-Gly2 and CAP-Gly3 residues that bind Ub are shown as blue and purple spheres respectively. CAP-Gly domains tested for Ub binding (**Extended Data 1**) that do not bind Ub are depicted as red stars, whereas CAP-Gly2 and CAP-Gly3 that bind Ub are represented as green stars.

Figure S3

A

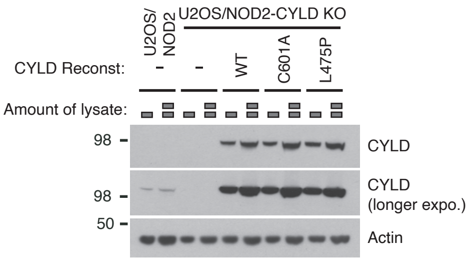

U2OS/ NOD2
U2OS/NOD2-CYLD KO
CYLD Reconst:
–
–
WT
C601A
L475P
Amount of lysate:
98
CYLD
98
CYLD (longer expo.)
50
Actin

B

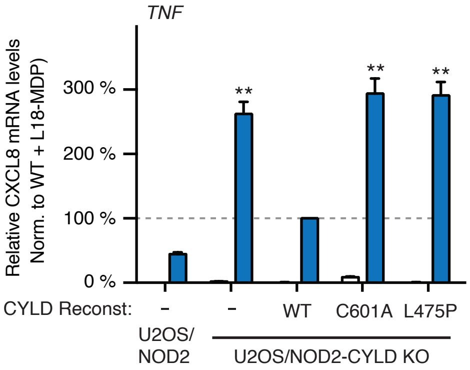

TNF
Relative CXCL8 mRNA levels Norm. to WT + L18-MDP)
300 %
200 %
100 %
0 %
**
**
**
CYLD Reconst:
–
–
WT
C601A
L475P
U2OS/ NOD2
U2OS/NOD2-CYLD KO

Figure S3: CAP-Gly3 of CYLD is required for regulation of Ub deposition and signaling outcome, Related to Figure 3.

(A) Western blot analysis of CYLD levels in U2OS/NOD2 CYLD KO cells with reintroduced CYLD mutants as indicated. (B) qRT-PCR of U2OS/NOD2 CYLD KO cell lines with reintroduced CYLD mutants to assess *TNF* mRNA levels. Cells were stimulated for 3 h with 200 ng/ml L18-MDP. Values were normalized to values from L18-MDP-treated CYLD KO cells reconstituted with $CYLD^{WT}$. Data shown represents mean ±SEM of at least 3 independent experiments.

Figure S4

A

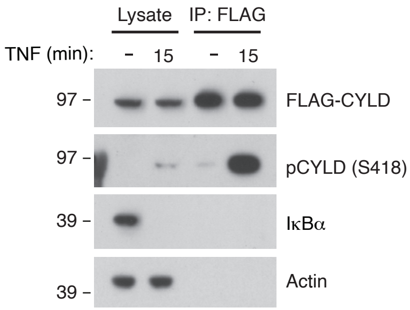
Lysate
IP: FLAG
TNF (min):
–
15
–
15
97 –
FLAG-CYLD
97 –
pCYLD (S418)
39 –
IκBα
39 –
Actin

Figure S4: TNF-induced phosphorylation of CYLD, Related to Figure 4.

(A) Immunoprecipitation of FLAG-tagged CYLD from lysates of U2OS/NOD2 CYLD KO cells expressing FLAG-CYLD^{WT} and stimulated or not with 20ng/ml TNF for 15 min. Lysates and IP samples were analyzed by western blotting as indicated before being processed for LC-MS/MS.

Figure S5

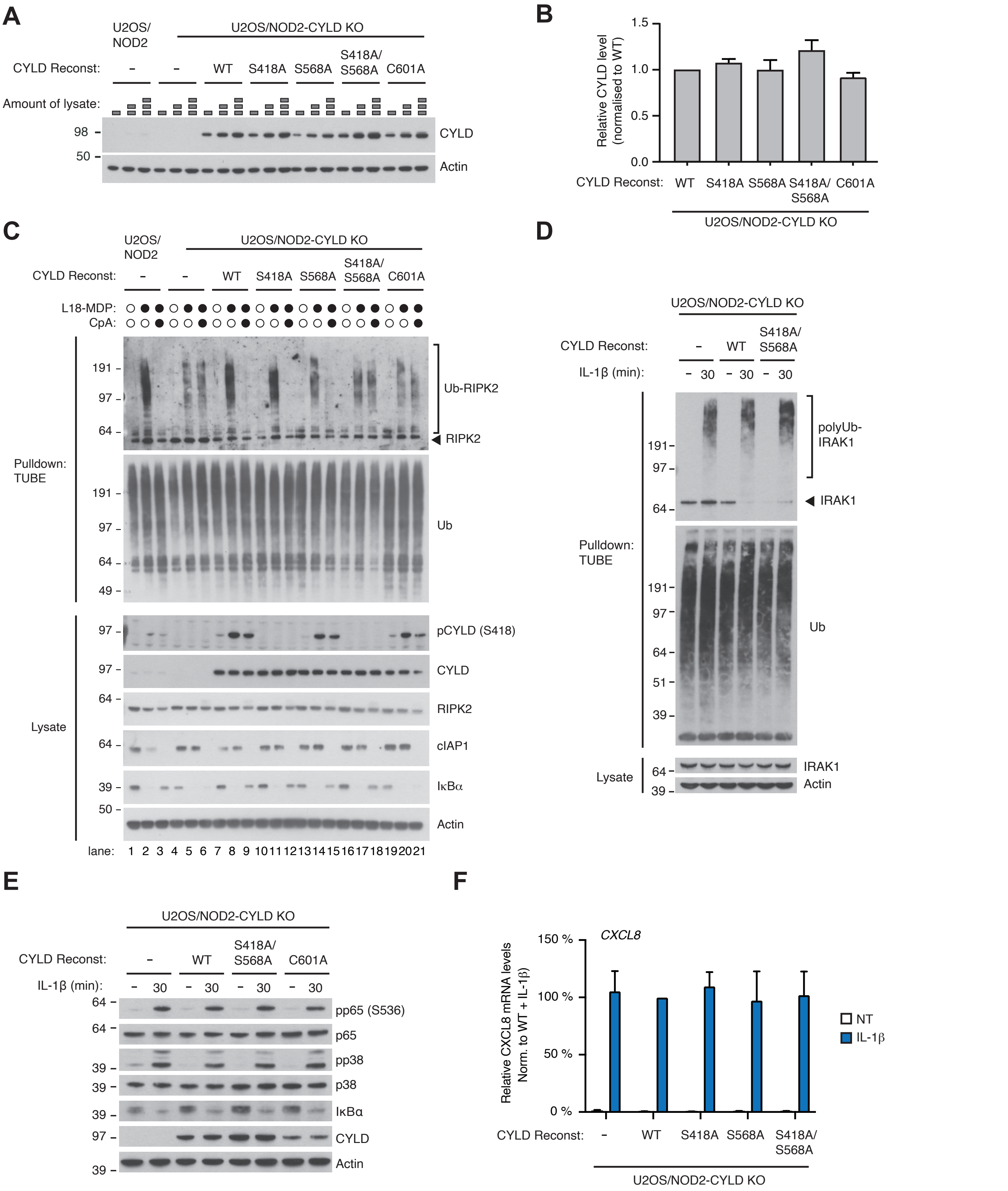

A
U2OS/ NOD2
U2OS/NOD2-CYLD KO
CYLD Reconst:
–
–
WT
S418A
S568A
S418A/ S568A
C601A
Amount of lysate:
98
50
CYLD
Actin
B
Relative CYLD level (normalised to WT)
1.5
1.0
0.5
0.0
CYLD Reconst:
WT
S418A
S568A
S418A/ S568A
C601A
U2OS/NOD2-CYLD KO
C
U2OS/ NOD2
U2OS/NOD2-CYLD KO
CYLD Reconst:
L18-MDP:
CpA:
191
97
64
Ub-RIPK2
RIPK2
Pulldown: TUBE
49
Ub
Lysate
pCYLD (S418)
CYLD
RIPK2
cIAP1
39
IκBα
50
Actin
lane: 1 2 3 4 5 6 7 8 9 10 11 12 13 14 15 16 17 18 19 20 21
D
U2OS/NOD2-CYLD KO
CYLD Reconst:
IL-1β (min):
30
polyUb- IRAK1
IRAK1
51
IRAK1
Actin
E
pp65 (S536)
p65
pp38
p38
F
CXCL8
Relative CXCL8 mRNA levels Norm. to WT + IL-1β)
150 %
100 %
50 %
0 %
NT
IL-1β

Figure S5: Ser568 phosphorylation together with Ser418 regulates CYLDs ability to control Ub deposition and signaling outcome after NOD2 but not IL-1β receptor stimulation, Related to Figure 5.

(A) Western blot analysis of CYLD levels in U2OS/NOD2 CYLD KO cells reconstituted with CYLD variants as indicated. (B) Densitometric quantification of CYLD levels in (A). Data shown represents mean ±SEM of 2 values. (C) Purification and western blot analysis of Ub conjugates from U2OS/NOD2 cell lines. Cells were treated for 1 h with CpA (1 μM) or DMSO prior to stimulation with 200 ng/ml L18-MDP for 1 h as indicated. Repeat experiment of data shown in Figure 5C to illustrate the partial reversal of RIPK2-Ub in cells expressing CYLD S568A. (D) Purification and western blot analysis of Ub conjugates from U2OS/NOD2 cell lines. Cells were treated for 30 min with 20 ng/ml IL-1β as indicated. (E) Western blot analysis to assess signaling after IL-1β treatment. Cells were treated with 10 ng/ml IL-1β for 30 min as indicated. (F) Relative levels of *CXCL8* mRNA in U2OS/NOD2 cell lines treated or not with 10 ng/ml IL-1β for 3 h. Data shown represents mean ±SEM of at least 3 independent experiments. *p < 0.05, **p < 0.01, *n.s.,* not significant.

Figure S6

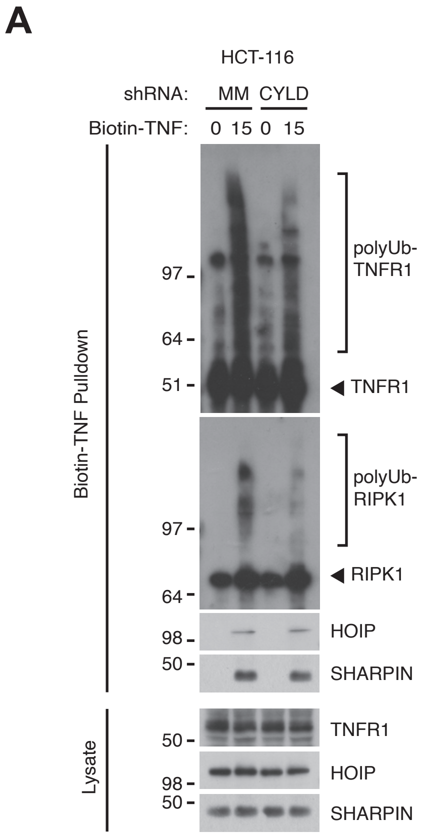
A
HCT-116
shRNA:
MM
CYLD
Biotin-TNF:
0
15
0
15
Biotin-TNF Pulldown
97
64
51
polyUb-
TNFR1
TNFR1
97
64
polyUb-
RIPK1
RIPK1
98
HOIP
50
SHARPIN
Lysate
50
TNFR1
98
HOIP
50
SHARPIN

Figure S6: The absence of CYLD activity interferes with ubiquitination of TNF-RSC components, Related to Figure 6.

(A) Purification and western blot analysis of the TNFR1 complex in HCT-116 cells with shRNA knockdown of CYLD or mismatch control. Cells were stimulated with 50 ng/ml Biotin-TNF for indicated time points. Samples used for western blots analysis are from the same experiment shown in Figure 4B.

Figure S7

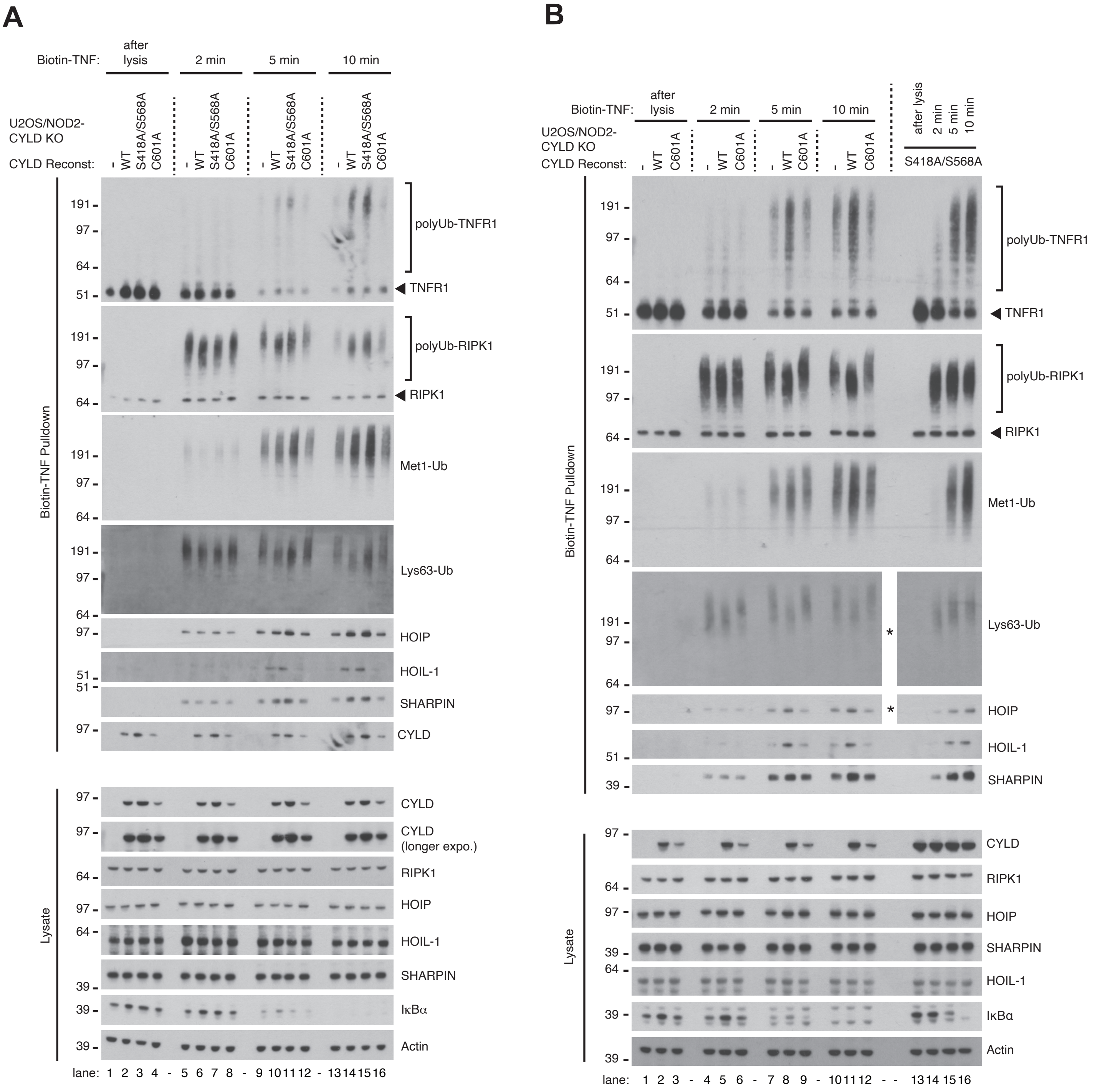
A
Biotin-TNF:
after lysis
2 min
5 min
10 min
U2OS/NOD2-CYLD KO
CYLD Reconst:
- WT S418A/S568A C601A
polyUb-TNFR1
TNFR1
polyUb-RIPK1
RIPK1
Met1-Ub
Lys63-Ub
HOIP
HOIL-1
SHARPIN
CYLD
Biotin-TNF Pulldown
CYLD (longer expo.)
IκBα
Actin
Lysate
lane: 1 2 3 4 - 5 6 7 8 - 9 10 11 12 - 13 14 15 16
B
Biotin-TNF:
after lysis
2 min
5 min
10 min
U2OS/NOD2-CYLD KO
CYLD Reconst:
- WT C601A
S418A/S568A
polyUb-TNFR1
TNFR1
polyUb-RIPK1
RIPK1
Met1-Ub
Lys63-Ub
*
HOIP
HOIL-1
SHARPIN
Biotin-TNF Pulldown
CYLD
RIPK1
HOIP
SHARPIN
HOIL-1
IκBα
Actin
Lysate
lane: 1 2 3 - 4 5 6 - 7 8 9 - 10 11 12 - - 13 14 15 16

Figure S7: Non-phosphorylation-mediated activity of CYLD retains LUBAC at the TNF-RSC and restores normal Met1-Ub, but not Lys63-Ub, deposition, Related to Figure 7.

(A and B) Independent repeats of the data shown in Figure 7A. Purification and western blot analysis of the TNFR1 complex in U2OS/NOD2 cell lines as indicated. Cells were stimulated with 50 ng/ml Biotin-TNF for indicated time points. Lanes 1-12 in (B) are also shown in Figure 6C. Asterisks in (B) indicate that the scanned blots for Lys63-Ub and HOIP have been cut and reassembled to align with other blots in the figure. Due to a loading error the CYLD$^{S418A/S586A}$ samples (lanes 13-16) were shifted one lane relative to the other blots.

Extended data 1

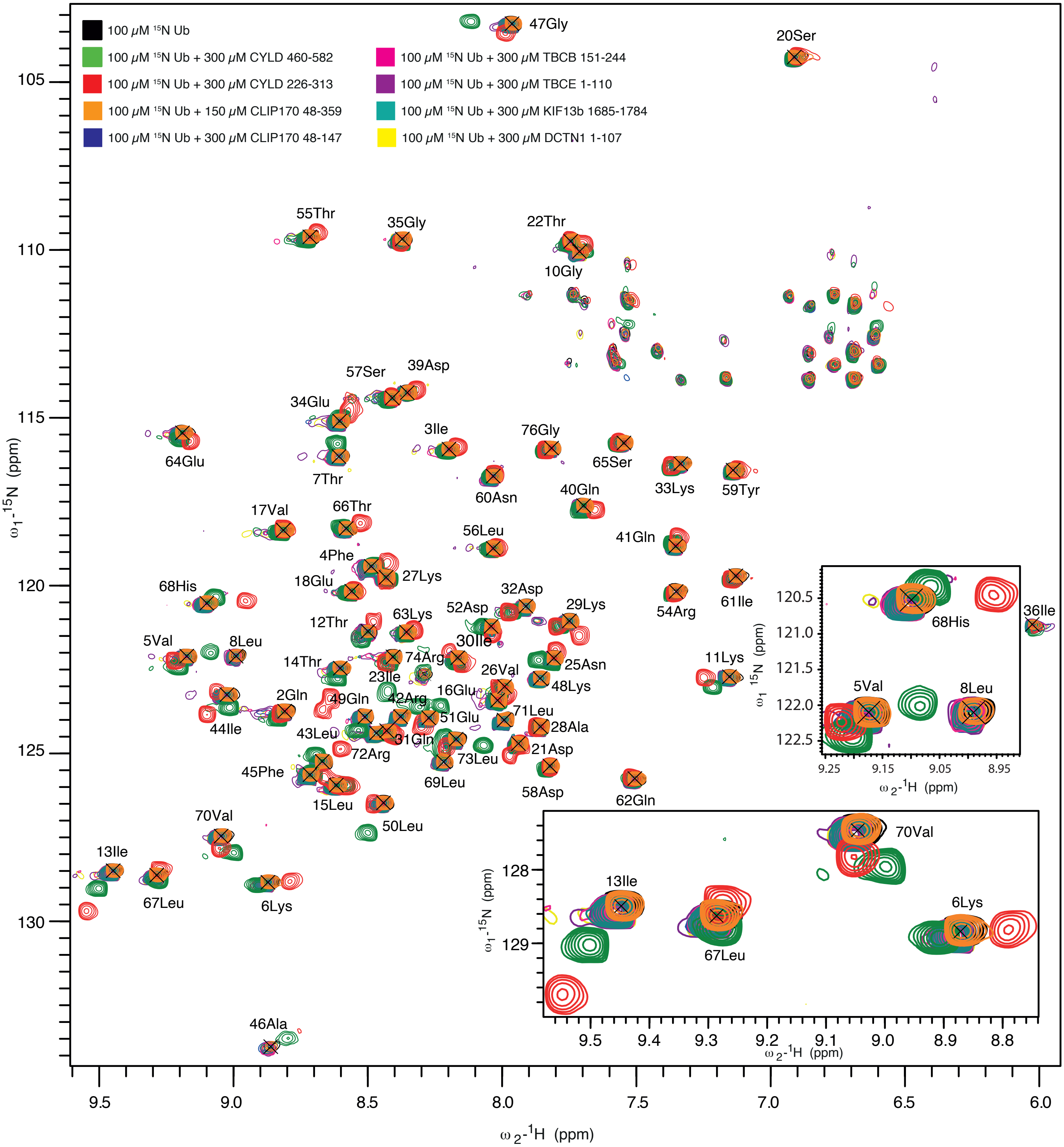

Extended Data 1. Map of chemical shift perturbations, Related to Figure 1.

BEST-TROSY spectra for ^{15}N-labelled Ub alone (black) or in the presence of three-fold molar excess CAP-Gly domains. Chemical shift perturbations are only observed for CYLD CAP-Gly2 and CAP-Gly3 domains, green and red respectively. Residue numbers correspond to resonance assignment of Ub are shown.

Extended data 2

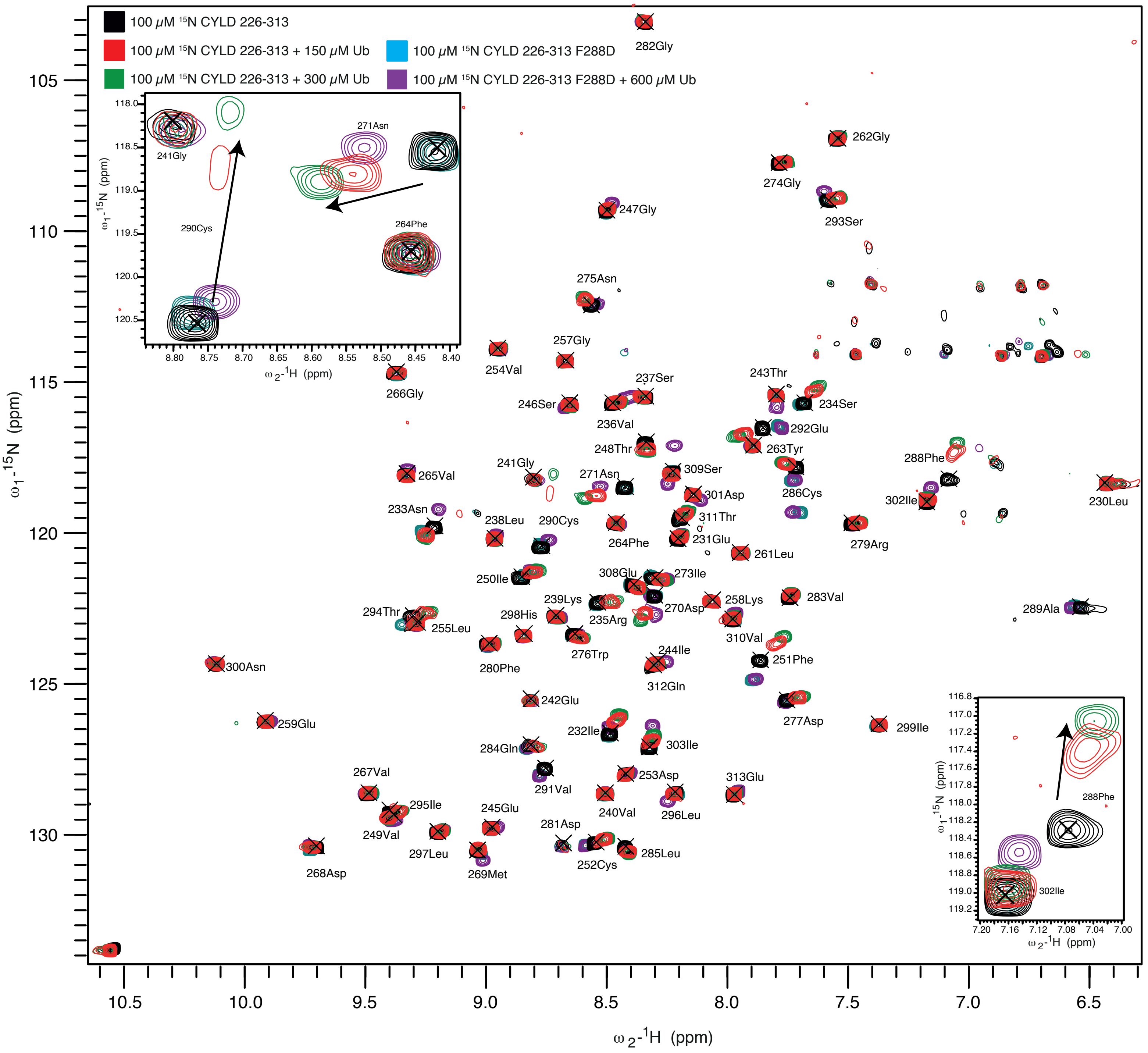

Extended Data 2. Map of chemical shift perturbations, Related to Figure 2.

BEST-TROSY spectra for ^{15}N-labelled CAP-Gly2 (black) or CAP-Gly2 F288D (blue) alone or in the presence of increasing concentrations of Ub. Complete backbone assignment of ^{13}C, ^{15}N CAP-Gly2 resonances permitted detailed interactions between CAP-Gly2 and Ub to be observed. Insert two regions show detailed titration of resonances Cys290 and Phe288.

Extended data 3

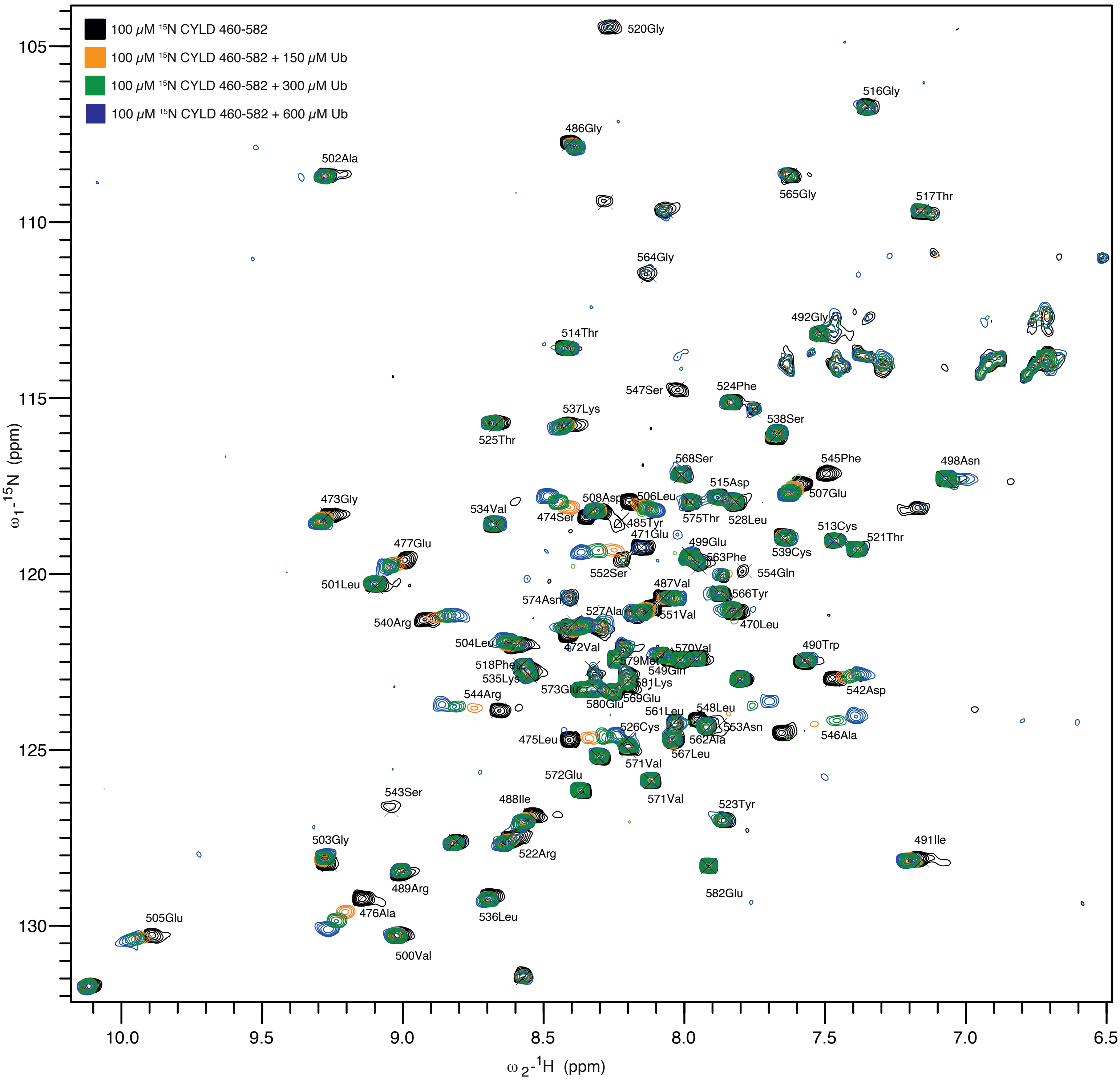

Extended Data 3. Map of chemical shift perturbations, Related to Figure 2.

BEST-TROSY spectra for ^{15}N-labelled CAP-Gly3 (black) with increasing concentrations of Ub. Resonance positions for CAP-Gly3 amino acids derived from complete backbone assignment of ^{13}C, ^{15}N CAP-Gly3 (460-582) spectra are shown.

Extended data 4

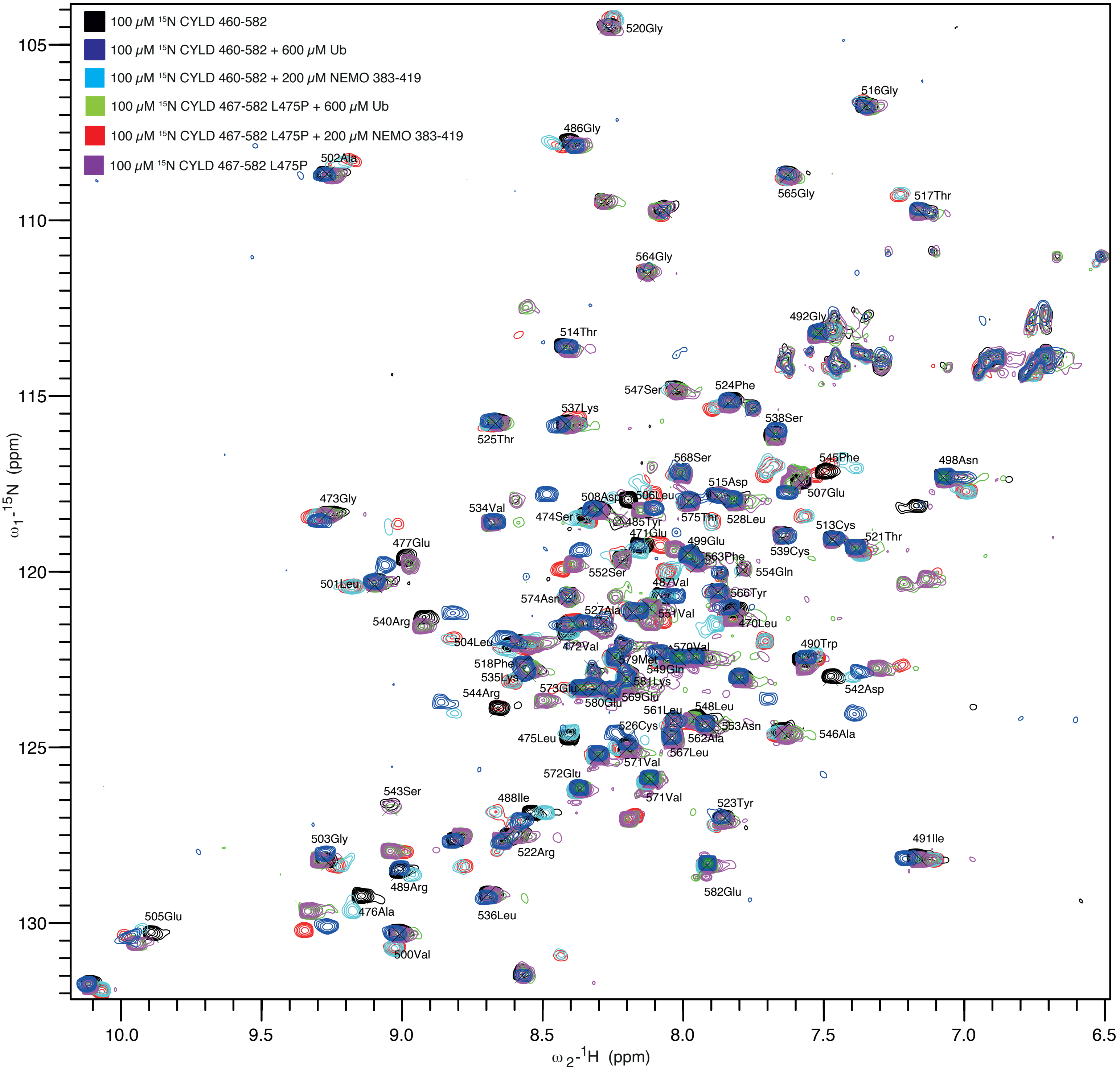

Extended Data 4. Map of chemical shift perturbations, Related to Figure 2.

BEST-TROSY spectra for ^{15}N-labelled CAP-Gly3 (black) in the presence of six-fold molar excess of Ub (dark blue) or two-fold molar excess of NEMO (light blue). The CAP-Gly3 Ub-binding mutant L475P, was recorded alone (purple) or in the presence of six-fold molar excess NEMO (red) exhibited similar chemical shift perturbations as WT CAP-Gly3. However, L475P is does not undergo any chemical shift perturbations in the presence of six-fold molar excess Ub (green).
