# Supplementary material for: Regulation of CYLD activity and specificity by phosphorylation and ubiquitin-binding CAP-Gly domains
Source: Cell Rep. 2021 Oct 5;37(1):109777. doi: 10.1016/j.celrep.2021.109777 (PMC8511506; doi:10.1016/j.celrep.2021.109777)
Supplement: Document S2. Article plus supplemental information [file mmc3.pdf]

Cell Reports

Article

Regulation of CYLD activity and specificity by phosphorylation and ubiquitin-binding CAP-Gly domains

Graphical abstract

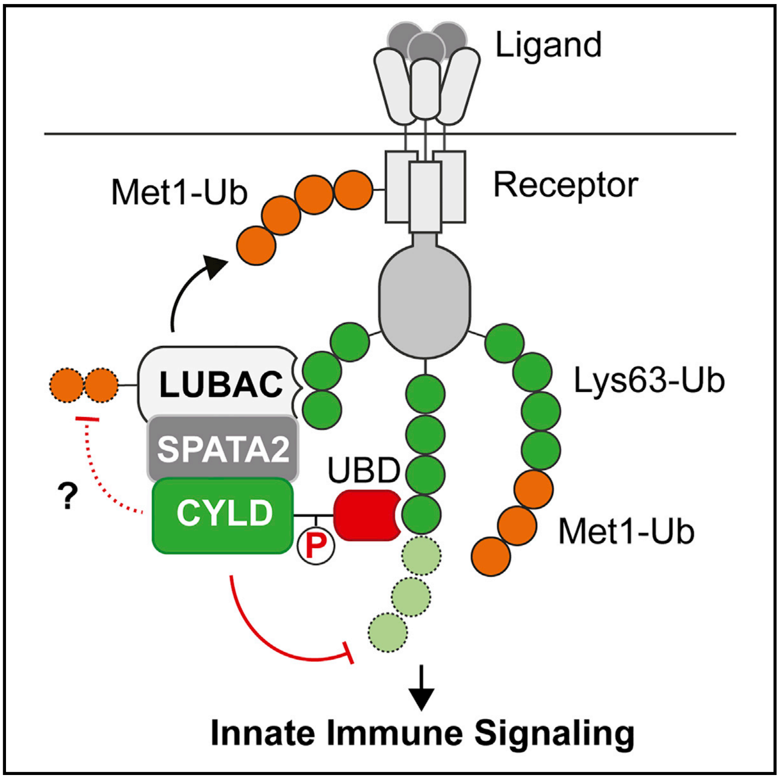

Highlights

- CAP-Gly domains in CYLD are ubiquitin (Ub)-binding domains
- CAP-Gly3 is indispensable for full CYLD catalytic activity
- Phosphorylation of Ser568 activates CYLD Lys63-Ub DUB activity
- Ub binding and phosphorylation impacts CYLD regulation of receptor signaling

Authors

Paul R. Elliott, Derek Leske, Jane Wagstaff, ..., Stefan M.V. Freund, David Komander, Mads Gyrd-Hansen

Correspondence

paul.elliott@bioch.ox.ac.uk (P.R.E.),
dk@wehi.edu.au (D.K.),
mgyrd@sund.ku.dk (M.G.-H.)

In brief

Elliott et al. show that the catalytic activity of CYLD relies on ubiquitin binding via its CAP-Gly3 domain, and they identify a phosphorylation switch that stimulates cleavage of Lys63-Ub. Cellular work provides evidence that phosphorylated CYLD balances Lys63- and Met1-linked polyubiquitin at receptor signaling complexes to promote LUBAC signaling.

Elliott et al., 2021, Cell Reports *37*, 109777
October 5, 2021 © 2021 The Author(s).
https://doi.org/10.1016/j.celrep.2021.109777

CellPress

Cell Reports

CellPress
OPEN ACCESS

Article

Regulation of CYLD activity and specificity by phosphorylation and ubiquitin-binding CAP-Gly domains

Paul R. Elliott,[1,2,8,*] Derek Leske,[3,8] Jane Wagstaff,[1] Lisa Schlicher,[3] Georgina Berridge,[4] Sarah Maslen,[1] Frederik Timmermann,[3] Biao Ma,[3] Roman Fischer,[4] Stefan M.V. Freund,[1] David Komander,[1,5,6,*] and Mads Gyrd-Hansen[3,7,9,*]

[1]Division of Protein and Nucleic Acid Chemistry, MRC Laboratory of Molecular Biology, Francis Crick Avenue, Cambridge CB2 0QH, UK
[2]Department of Biochemistry, University of Oxford, South Parks Road, Oxford OX1 3QU, UK
[3]Ludwig Institute for Cancer Research, University of Oxford, Old Road Campus Research Building, Off-Roosevelt Drive, Oxford OX3 7DQ, UK
[4]TDI Mass Spectrometry Laboratory, Target Discovery Institute, Nuffield Department of Medicine, University of Oxford, Roosevelt Drive, Oxford OX3 7FZ, UK
[5]The Walter and Eliza Hall Institute of Medical Research, 1G Royal Parade, Parkville VIC 3052, Australia
[6]Department for Medical Biology, University of Melbourne, Melbourne VIC 3000, Australia
[7]LEO Foundation Skin Immunology Research Center, Department of Immunology and Microbiology, University of Copenhagen, Maersk Tower, Blegdamsvej 3B, 2200 Copenhagen, Denmark
[8]These authors contributed equally
[9]Lead contact
*Correspondence: paul.elliott@bioch.ox.ac.uk (P.R.E.), dk@wehi.edu.au (D.K.), mgyrd@sund.ku.dk (M.G.-H.)
https://doi.org/10.1016/j.celrep.2021.109777

SUMMARY

Non-degradative ubiquitin chains and phosphorylation events govern signaling responses by innate immune receptors. The deubiquitinase CYLD in complex with SPATA2 is recruited to receptor signaling complexes by the ubiquitin ligase LUBAC and regulates Met1- and Lys63-linked polyubiquitin and receptor signaling outcomes. Here, we investigate the molecular determinants of CYLD activity. We reveal that two CAP-Gly domains in CYLD are ubiquitin-binding domains and demonstrate a requirement of CAP-Gly3 for CYLD activity and regulation of immune receptor signaling. Moreover, we identify a phosphorylation switch outside of the catalytic USP domain, which activates CYLD toward Lys63-linked polyubiquitin. The phosphorylated residue Ser568 is a novel tumor necrosis factor (TNF)-regulated phosphorylation site in CYLD and works in concert with Ser418 to enable CYLD-mediated deubiquitination and immune receptor signaling. We propose that phosphorylated CYLD, together with SPATA2 and LUBAC, functions as a ubiquitin-editing complex that balances Lys63- and Met1-linked polyubiquitin at receptor signaling complexes to promote LUBAC signaling.

INTRODUCTION

Ubiquitination and phosphorylation constitute dynamic post-translational modifications (PTMs) that regulate a wide variety of cellular processes, including inflammatory signaling and innate immune responses (Kulathu and Komander, 2012). Polymeric ubiquitin (Ub) chains control signaling downstream of innate immune receptors such as nuclear oligomerization domain (NOD)-like receptors (NLRs) and tumor necrosis factor (TNF) receptor 1 (TNFR1) to activate nuclear factor κB (NF-κB)-mediated gene activation (Hrdinka and Gyrd-Hansen, 2017). Receptor stimulation leads to assembly of receptor signaling complexes (RSCs) where Ub E3 ligases facilitate assembly of Ub chains linked via lysine 63 (Lys63-Ub) and methionine 1 ("linear" Ub, hereafter Met1-Ub) on receptor-interacting kinases (RIPKs) and other protein substrates at the RSC to activate kinase signaling cascades via the transforming growth factor (TGF)-β-activated kinase (TAK1) and inhibitor of κB-kinase (IKK) complexes (Hrdinka and Gyrd-Hansen, 2017). The deubiquitinases (DUBs) CYLD, OTULIN, and A20 play critical roles in restricting signaling through regulation of Ub landscapes at RSCs. Indeed, mutations affecting the activity of these DUBs give rise to inflammatory immune disorders and can cause cancer (Bignell et al., 2000; Damgaard et al., 2016, 2020; Harhaj and Dixit, 2011; Verboom et al., 2020; Zhou et al., 2016).

The linear Ub chain assembly complex (LUBAC) consists of HOIP, HOIL-1, and SHARPIN and appears to be the sole Ub ligase responsible for conjugating Met1-Ub in cells (Elliott, 2016). LUBAC is required for productive inflammatory signaling and is regulated by OTULIN and CYLD, which associate with LUBAC's catalytic subunit HOIP via the HOIP PUB domain. While OTULIN binds to HOIP directly, CYLD binds to HOIP via an adaptor, SPATA2 (Draber et al., 2015; Elliott et al., 2014, 2016; Hrdinka et al., 2016; Schaeffer et al., 2014; Schlicher et al., 2016; Takiuchi et al., 2014; Wagner et al., 2016). It is currently thought that OTULIN controls global Met1-Ub levels and auto-ubiquitination of

This is an open access article under the CC BY license (http://creativecommons.org/licenses/by/4.0/).

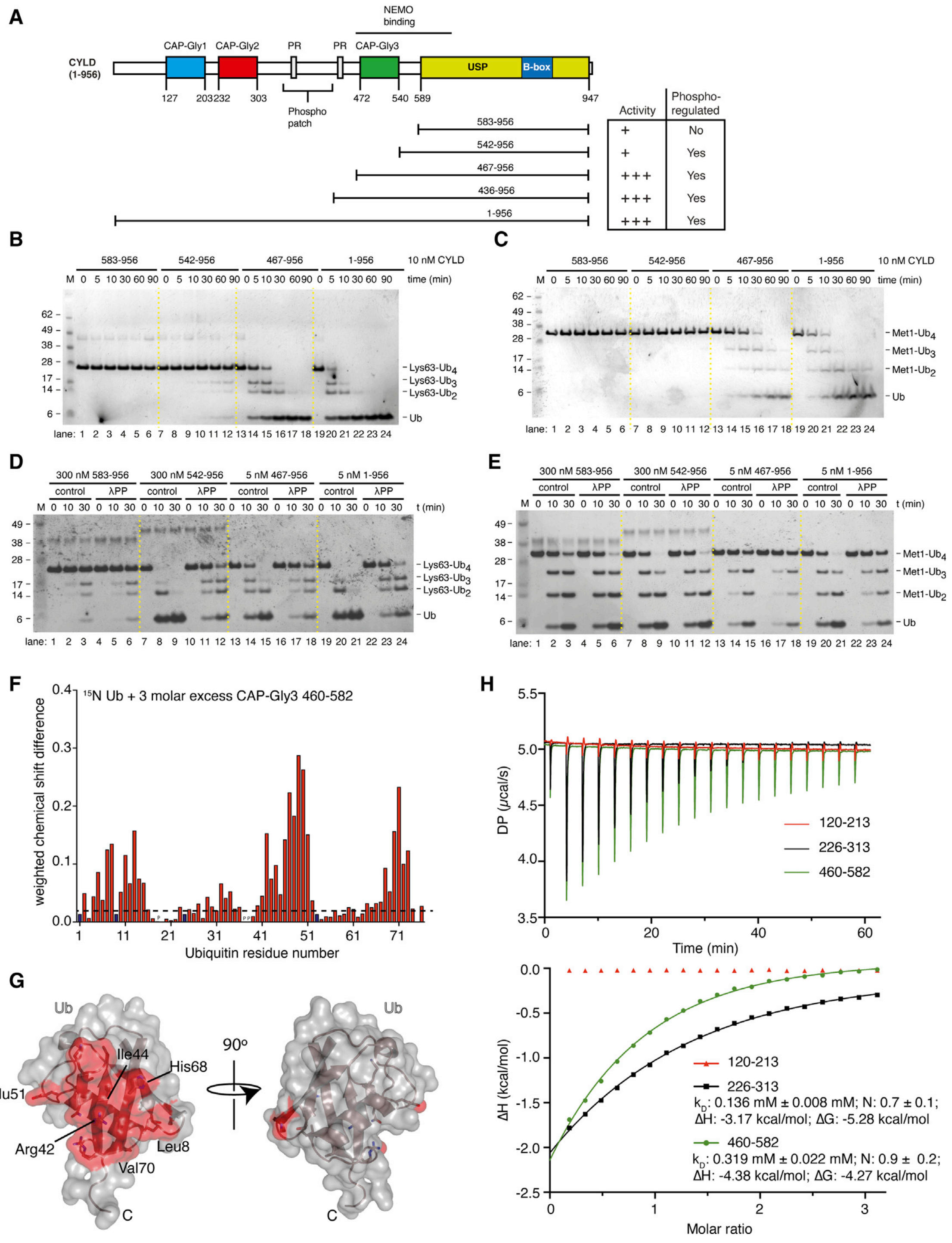

Figure 1. CYLD is a Lys63-DUB reliant on extra-catalytic domains and phosphorylation for full activity

(A) Schematic representation of the constructs of CYLD generated to test enzymatic activity and reliance on phosphorylation. Table summarizes data shown in subsequent panels.

(B) Qualitative DUB assays for assessing CYLD enzyme activity. Lys63-Ub4 was incubated with different CYLD fragments, and Ub cleavage was monitored over 90 min. Samples were resolved by SDS-PAGE and stained using silver stain.

(C) Qualitative DUB assays as in (B) but using Met1-Ub4 as a substrate. CYLD enzyme activity at 200 nM concentration is shown in Figures S1A and S1B.

(D) Qualitative DUB assay to investigate the phosphorylation status of purified CYLD variants from sf9 cells. sf9-purified CYLD variants were either incubated with λPP or in λPP buffer (control) and then used in a DUB assay as in (B).

(legend continued on next page)

LUBAC, while CYLD restricts ubiquitination primarily at RSCs to regulate inflammatory signaling and cell death (Draber et al., 2015; Elliott et al., 2016; Heger et al., 2018; Hrdinka et al., 2016; Kupka et al., 2016; Schlicher et al., 2016; Wagner et al., 2016). While some of the aspects of DUB-mediated regulation of inflammation have become clearer in recent years, the specific details of how DUBs are regulated themselves are often not well understood. DUBs are subject to many layers of regulation (Mevissen and Komander, 2017; Sahtoe and Sixma, 2015) and their activity and linkage preference can be modulated by direct modification such as phosphorylation (Huang et al., 2012; Wertz et al., 2015; Zhao et al., 2018). Moreover, DUB exo-domains, i.e., domains outside of the catalytic domain, may alter DUB activity and specificity; of particular relevance in many DUBs are Ub-binding domains (UBDs) (Clague et al., 2013; Mevissen and Komander, 2017).

CYLD is a comparably well-studied DUB in terms of its activity, specificity, and regulation. It is an unusual and divergent member of the Ub-specific protease (USP) family of DUBs, since its structurally distinct USP domain preferentially hydrolyses Met1- and Lys63-Ub (Komander et al., 2008, 2009; Sato et al., 2015). The catalytic domain harbors a dimerization-inducing B-box module (Komander et al., 2008) and is sufficient to bind to the adaptors SPATA2 and SPATA2L (Elliott et al., 2016; Kupka et al., 2016; Schlicher et al., 2016; Wagner et al., 2016); SPATA2 binding enhances CYLD activity by 2- to 3-fold but does not change linkage preference (Elliott et al., 2016). Upstream of its C-terminal catalytic domain, CYLD harbors three cytoskeleton-associated protein glycine-rich (CAP-Gly1/2/3) domains, which are ~70-aa SRC homology 3 (SH3)-fold protein interaction domains known to bind to microtubules (Yan et al., 2015). The first and second CYLD CAP-Gly domains indeed facilitate interaction with microtubules, whereas CYLD CAP-Gly3 interacts with NEMO/IKKγ, the non-catalytic subunit of the IKK complex (Gao et al., 2008; Saito et al., 2004; Wickström et al., 2010). CYLD is phosphorylated by IKKs on multiple Ser/Thr residues within a "phospho patch" in the linker region between CAP-Gly2 and CAP-Gly3 (aa 344–444, see https://www.phosphosite.org/homeAction.action) (Hutti et al., 2009; Reiley et al., 2005; Thein et al., 2014). However, it remains controversial how phosphorylation affects CYLD, as it has been reported to both increase and suppress CYLD activity (Hutti et al., 2009; Reiley et al., 2005; Thein et al., 2014). At this stage, it is unclear whether and how regions outside of the CYLD catalytic domain directly regulate CYLD activity and function.

Here, we report that the CAP-Gly2 and CAP-Gly3 of CYLD are novel UBDs and demonstrate that CAP-Gly3 is indispensable for full CYLD catalytic activity *in vitro* and for CYLD-regulated NOD2 signaling in cells. Furthermore, we identify a previously unannotated phosphorylation site, Ser568, which stimulates CYLD's Lys63-Ub DUB activity and that, in concert with Ser418 phosphorylation, contributes to CYLD-dependent regulation of NOD2 and TNFR1 signaling.

RESULTS

N-terminally extended CYLD variants improve activity and change CYLD specificity

The wealth of data on the biological function of CYLD are currently in contrast to the limited biochemical and mechanistic understanding of the full-length enzyme. While the C-terminal catalytic USP domain has been studied in molecular detail (Komander et al., 2008, 2009; Sato et al., 2015), it has remained unclear whether and how the CYLD N-terminal regions impact CYLD activity and specificity. Reported regulation by phosphorylation have not been mechanistically understood and have led to conflicting reports (Hutti et al., 2009; Reiley et al., 2005).

We revisited activity and linkage preference studies with full-length and N-terminally truncated variants of human CYLD purified from insect cells (Figure 1A). Analysis of CYLD variants in *in vitro* DUB assays with Lys63-Ub4 and Met1-Ub4 confirmed that the isolated USP domain (aa 583–956) preferentially hydrolyzed Met1-Ub4 over Lys63-Ub4 (Figures 1B and 1C; compare lanes 1–6) (Komander et al., 2008; Sato et al., 2015). Strikingly, all N-terminally extended USP domain constructs showed significantly higher activity, in particular toward Lys63-Ub4 (Figures 1B, 1C, S1A, and S1B). The much higher activity of full-length CYLD was obtained by constructs just encompassing the third CAP-Gly domain (CAP-Gly3; aa 467–543) (Figures 1B and 1C; compare lanes 13–24), which significantly improved activity toward both Lys63- and Met1-Ub4. However, in addition to improved activity, a reversal of specificity was clear, as extended variants of CYLD preferentially cleaved Lys63-Ub over Met1-Ub (Figures 1B and 1C). These experiments showed that Lys63-Ub cleavage is activated by regions outside a barely active catalytic domain; the effect of these regions for Met1 cleavage seems less pronounced (Figures 1B and 1C). Our results reveal that the N-terminal part of CYLD is essential for CYLD activity and contributes to linkage specificity.

Proteins purified from insect cells are often phosphorylated by insect cell kinases. Indeed, full-length CYLD was phosphorylated on Ser418 as detected by a phospho-specific antibody (Gringhuis et al., 2014) (Figure S1C and see below). Although most of our constructs did not encompass the Ser418 phosphorylation site (see Figure 1A), we nonetheless tested whether phosphorylation of CYLD affects its activity *in vitro*. CYLD variants were dephosphorylated by incubation with recombinant lambda protein phosphatase (λPP) before performing DUB assays. Strikingly, dephosphorylation of full-length CYLD led to a sharp reduction in cleavage of Lys63-Ub4 while it had a more modest effect on cleavage of Met1-Ub4 (Figures 1D and 1E, compare lanes 19–21 with lanes 22–24). The inhibition of Lys63-Ub4 activity by dephosphorylation was evident for all tested CYLD

(E) As in (D) using Met1-Ub4 chains as substrate.

(F) Chemical shift perturbation plot of Ub for titration of 3-fold excess of CAP-Gly3 (aa 460–582).

(G) Surface of Ub (gray) with chemical shifts mapped from (F) showing that CAP-Gly3 binds the Ub Ile44 patch.

(H) Isothermal titration calorimetry thermograms of the three CYLD CAP-Gly domains. Raw isotherms are shown (top) with integrated fits (bottom) for CAP-Gly2 and CAP-Gly3.

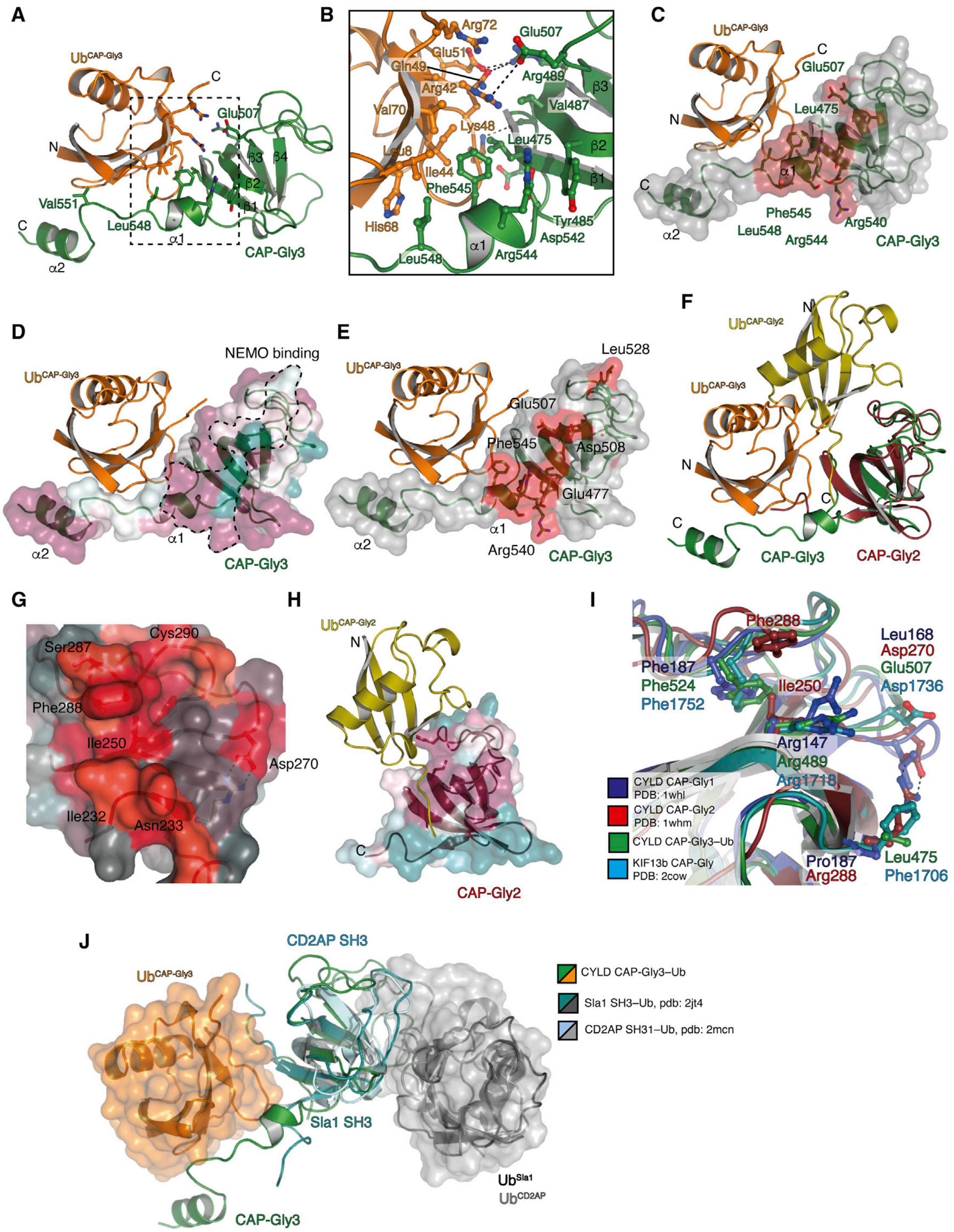

Figure 2. CAP-Gly domains 2 and 3 of CYLD bind Ub

(A) Crystal structure of CAP-Gly3 (aa 464–565, green) to Ub (orange).

(B) Close-up view of the CAP-Gly3-Ub interface from (A). Interacting residues are shown as sticks.

(C) Surface of CAP-Gly3 (gray) with chemical shift perturbations (red) confirming the interaction interface is the same in solution.

(D) Surface view of CAP-Gly3 bound to Ub with residues colored onto the CAP-Gly3 surface based on conservation among different CYLD orthologs. Conservation scores were calculated with the Consurf server (Landau et al., 2005); purple, most conserved; blue, least conserved. Region interacting with NEMO, mapped by NMR in (E), is shown as a dotted line.

(E) Surface of CAP-Gly3 (gray) with chemical shift perturbations (red) of the interaction of CAP-Gly3 with NEMO ZnF.

(legend continued on next page)

variants except for the isolated USP domain (Figures 1D and 1E). The shortest extension comprised merely 40 residues upstream of the USP domain (CYLD 542–956), yet the extended constructs were markedly more active compared to the catalytic core (Figures 1B and 1D; compare lanes 7–9 with lanes 1–3). The additional activity was reversed by λPP treatment (Figure 1D), suggesting additional phosphorylation sites in this region that stimulate cleavage of Lys63-Ub. Phosphorylation regulated cleavage of Met1-Ub chains to a lesser extent; all constructs showed only a mild decrease in cleavage of this chain type after dephosphorylation (Figure 1E). The set of *in vitro* biochemical data presented above begged for more detailed molecular and cellular understanding.

CYLD CAP-Gly domains 2 and 3 are novel UBDs

Even without phosphorylation and in the absence of other factors (e.g., NEMO), we noted significantly higher activity in CYLD constructs that included CAP-Gly3 (Figure 1D, note the 60-fold lower enzyme concentration for extended CYLD constructs). We hence tested whether the CAP-Gly domain had the ability to bind Ub directly. Nuclear magnetic resonance (NMR) is an excellent method to uncover weak protein-protein interactions often found in UBDs. We calculated weighted chemical shift perturbations for ^{15}N-isotopically labeled Ub with 3-fold molar excess of isolated, purified CAP-Gly3 (aa 460–582, Figures 1F and S1D; Data S1). Robust chemical shifts indicated a strong binding event with a characteristic footprint, which when mapped onto the structure of Ub covers the Ile44 patch (Figure 1G), the canonical site for UBD interactions. Identical experiments with isolated CAP-Gly2 (aa 226–313) revealed that CAP-Gly2 also binds Ub through the Ile44 patch (Figures S1D and S1E; Data S1).

We next determined Ub binding characteristics and affinity by isothermal titration calorimetry (ITC). While CAP-Gly1 did not show any detectable binding to Ub, CAP-Gly2 and CAP-Gly3 bound Ub with high micromolar affinities (Figure 1H). Although these prevent us from calculating absolute dissociation constants, these affinities are within a common range for UBD/Ub interactions (Raasi et al., 2005) and identify the CYLD CAP-Gly domains as new UBDs.

Molecular details of the CAP-Gly3-Ub interaction

CAP-Gly domains comprise a five-strand β sheet topology and show similarities to SH3 domains; a subset of SH3 domains binds Ub (Stamenova et al., 2007). A detailed understanding of how CYLD CAP-Gly domains bind Ub came from two crystal structures of the CAP-Gly3 domain bound to Ub determined at 1.7 and 1.9 Å resolution (Figure 2A; Table S1). To obtain crystals, we slightly extended the CAP-Gly domains C-terminally; crystal form 1 (aa 467–552, 1.7 Å) crystallized in the tetragonal space group $P4_12_12$ and crystal form 2 (aa 467–565, 1.9 Å) crystallized in the orthorhombic space group $P2_12_12_1$. Both structures were solved by molecular replacement using the previously published NMR structure of CAP-Gly3 (PDB: 1IXD) and Ub (PDB: 1UBQ) and are highly similar with an overall root-mean-square deviation (RMSD) of 0.64 Å (Figure S2A). Also, the Ub binding mode between the two structures is highly similar, with hydrophobic CAP-Gly3 residues Leu475 and Val487 engaging the Ile44 patch of Ub (Figure 2B). In addition, hydrogen bonds are formed between CAP-Gly3 Arg488 and Glu507 with Ub Glu51 and Arg42, respectively. Interestingly, the extended C terminus of CAP-Gly3 (from aa 542–565) adopts two short helices, and Phe545 from helix1 forms further hydrophobic interactions with Ub Ile44. Finally, an additional residue toward the C terminus, Val551, engages with Ub in crystal form 2; however, this interaction may be artificially stabilized through crystal contacts (Figure S2B).

We used NMR as a complementary method to study Ub binding to CYLD CAP-Gly3. Uniformly ^{15}N,^{13}C-labeled CAP-Gly3 (aa 460–582) allowed for unambiguous assignment of backbone resonances (Data S3 and S4). Chemical shift perturbation mapping of CAP-Gly3 bound to 3-fold molar excess of Ub revealed a comparable Ub interface in solution studies as compared to the crystal structures (Figures 2C and S2C). Importantly, resonances for Leu475 and Phe545 were perturbed upon addition of Ub, while Val551 remained unperturbed (Figure S1F), suggesting that this interaction may indeed arise from a crystal contact. Residues from CAP-Gly3 that engage with Ub are conserved among CYLD orthologs, whereas Val551 is less well conserved (Figure 2D).

Mutation of Leu475 to either Pro (as in non-Ub binding CAP-Gly1) but also Arg (representative of CAP-Gly2 and most other CAP-Gly domains, Figure S2E) abrogated Ub binding without significantly perturbing the structure of the CAP-Gly domain (Figure S2C; Data S4). Importantly, CAP-Gly3 L475P was still able to bind to the NEMO ZnF domain. The chemical shift perturbations within labeled CAP-Gly3 upon NEMO binding mapped to a separate highly conserved surface, revealing that NEMO and Ub interactions are independent of one another (Figures 2E and S2D).

Ub binding in CAP-Gly2 and other CAP-Gly domains

CAP-Gly2 also bound Ub (Figure 1H), but an Arg in the Leu475 position already indicated that it may utilize a distinct binding mode (Figure S2E). Ub co-crystallization attempts with CAP-Gly2 were unsuccessful, and thus we utilized NMR and HADDOCK docking to generate a model of CAP-Gly2 bound to

(F) Superimposition of the CAP-Gly3-Ub structure onto an NMR-derived HADDOCK model of CAP-Gly2 binding to Ub (red and yellow, cartoon respectively), revealing an offset Ub-binding site for the two CAP-Gly domains.

(G) Surface of CAP-Gly2 (gray) with chemical shift perturbations (red) revealing the offset Ub-binding interface.

(H) Surface view of CAP-Gly2, colored by conservation as in (D), revealing that the Ub-interaction surface is conserved and offset to CAP-Gly3.

(I) Superimposition of the three CAP-Gly domains of CYLD: CAP-Gly1 (aa 120–213, PDB: 1whl, blue), CAP-Gly2-Ub (aa 226–313, PDB: 1whm, red), CAP-Gly3-Ub (aa 467–565, green), and KIF13b (aa 1685–1771, PDB: 2cow, cyan). Residues that coordinate Ub from the two different CAP-Gly2 and CAP-Gly3 interfaces are shown.

(J) Superimposition of SH3-Ub structures from Sla1 (PDB: 2jt4) and Cd2ap (PDB: 2mcn) onto CAP-Gly3, revealing the different modes of Ub binding by SH3 domains and CYLD CAP-Gly3.

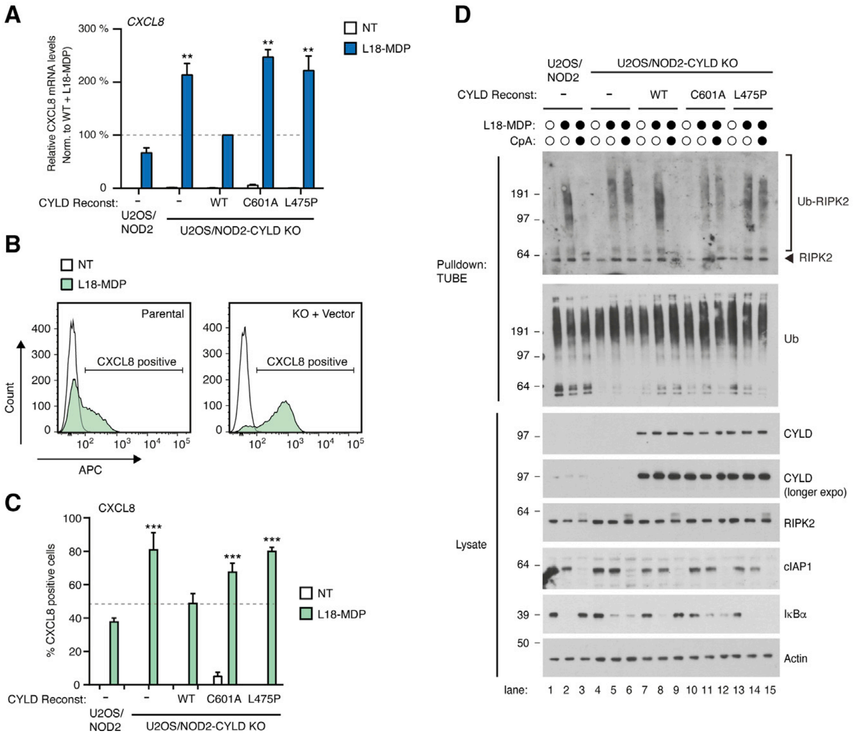

Figure 3. CAP-Gly3 of CYLD is required for regulation of Ub deposition and signaling outcome

(A) Relative levels of *CXCL8* mRNA in U2OS/NOD2 cell lines treated or not for 3 h with 200 ng/mL L18-MDP. mRNA levels are expressed relative to L18-MDP-treated CYLD KO cells reconstituted with WT CYLD. Data represent mean ± SEM of three to eight independent experiments.

(B) Histogram plots showing intracellular flow cytometry analysis of CXCL8-positive U2OS/NOD2 cell lines following stimulation with L18-MDP for 5 h in the presence of brefeldin A and monensin to block secretion of CXCL8.

(C) CXCL8 production in U2OS/NOD2 cell lines as in (B). Data represent mean ± SEM of three independent experiments.

(D) Purification and western blot analysis of Ub conjugates from U2OS/NOD2 cell lines treated for 1 h with CpA (1 μM) or DMSO prior to stimulation with L18-MDP for 1 h as indicated. $^{**}p < 0.01$, $^{***}p < 0.001$; n.s., not significant.

Ub, using a Rosetta-derived model from the assigned CYLD CAP-Gly2 backbone resonances. Surprisingly, all lowest energy minimized models generated from HADDOCK placed Ub on a different interface relative to the CAP-Gly3-Ub structure (Figure 2F). For CAP-Gly2, the Ub is rotated some 45 degrees relative to the binding of CAP-Gly3, and this is reflected in the distribution of chemical shift perturbations along the β1–β3 interface of the CAP-Gly2 domain (Figures 2G and S1G). Analysis of residues conserved among CAP-Gly2 orthologs reveals that the interface identified in HADDOCK is the most conserved part of the domain (Figure 2H), and this was further confirmed by mutagenesis and NMR studies, where mutation of CAP-Gly2 Phe288 to Asp (F288D) abrogated its interaction with Ub (Data S2).

Hence, while our data reveal CYLD CAP-Gly2 and CAP-Gly3 to be novel UBDs, they interact with the Ub Ile44 patch distinctly, through either a central Leu475 (CAP-Gly3) or through a central Phe288 (CAP-Gly2) (Figures 2I and S2E). As mentioned previously, CAP-Gly domains share similarity with SH3 domains, some of which bind Ub. Superposition of CAP-Gly2/3-Ub structures with Ub complexes of Sla1 and CD2AP SH3 domains (PDB: 2JT4 and 2MCN) (He et al., 2007; Ortega Roldan et al., 2013) revealed entirely different Ub interaction modes, in which Ub engages opposing surfaces of each domain type (Figure 2J).

Distinct Ub binding modes for two of the three CYLD CAP-Gly domains, and similarity to SH3 domains, posed the possibility that other CAP-Gly domains may also bind Ub. CAP-Gly domains of six proteins were expressed, purified, and tested for Ub binding by NMR, but of all the CAP-Gly domains tested, only CYLD CAP-Gly2 and CAP-Gly3 bound Ub (Figure S2E; Data S1). As noted previously, CAP-Gly domains (except CAP-Gly3 and KIF13b) have either Pro or Arg at an analogous position to Leu475 that would prevent a CYLD CAP-Gly3-like Ub binding mode (Figures 2I and S2E). Although KIF13b contains a hydrophobic residue (Phe) at the equivalent position to Leu475 (Figure 2I), Ub binding by NMR was not detected, suggesting that additional features such as an extended C terminus and/or hydrophobic residues along the β1–β3 interface are required for Ub binding.

Similarly, while the Ub-binding residue Phe288 in CAP-Gly2 is conserved, it faces toward the hydrophobic core in other CAP-Gly structures known to date and thus would not be available for Ub interactions. Therefore, of the CAP-Gly domains tested, only CYLD CAP-Gly2 and CAP-Gly3 constitute novel UBDs.

Understanding the importance of the CAP-Gly3 UBD

In vitro, inclusion of CAP-Gly3 is essential for full CYLD DUB activity, and we investigated whether this impacted CYLD's ability to regulate innate immune signaling. For this, CYLD knockout (KO) U2OS/NOD2 cells (Elliott et al., 2016) were stably reconstituted with CYLD variants for subsequent analyses of receptor signaling processes (Figure S3A). Wild-type CYLD ($CYLD^{WT}$), a catalytically dead mutant ($CYLD^{C601A}$), or the CAP-Gly3 Ub-binding mutant ($CYLD^{L475P}$) was expressed at similar levels albeit at much higher levels than endogenous CYLD, indicating that the introduced point mutations did not affect protein stability (Figure S3A). Of note, the level of $CYLD^{C601A}$ decreased over time with passaging of the cells, possibly because the mutation has a mild negative effect on proliferation and/or viability of the cells. Endogenous CYLD restricts inflammatory signaling mediated through the intracellular pattern recognition receptor NOD2 (Hrdinka et al., 2016) (Figures 3A–3C and S3B) and, as expected, $CYLD^{WT}$ restored L18-MDP-induced expression of NF-κB response genes and production of the chemokine CXCL8 (also termed interleukin [IL]-8) to the levels measured in parental U2OS/NOD2 cells (Figures 3A–3C and S3B). In contrast, cells reconstituted with

CellPress
OPEN ACCESS

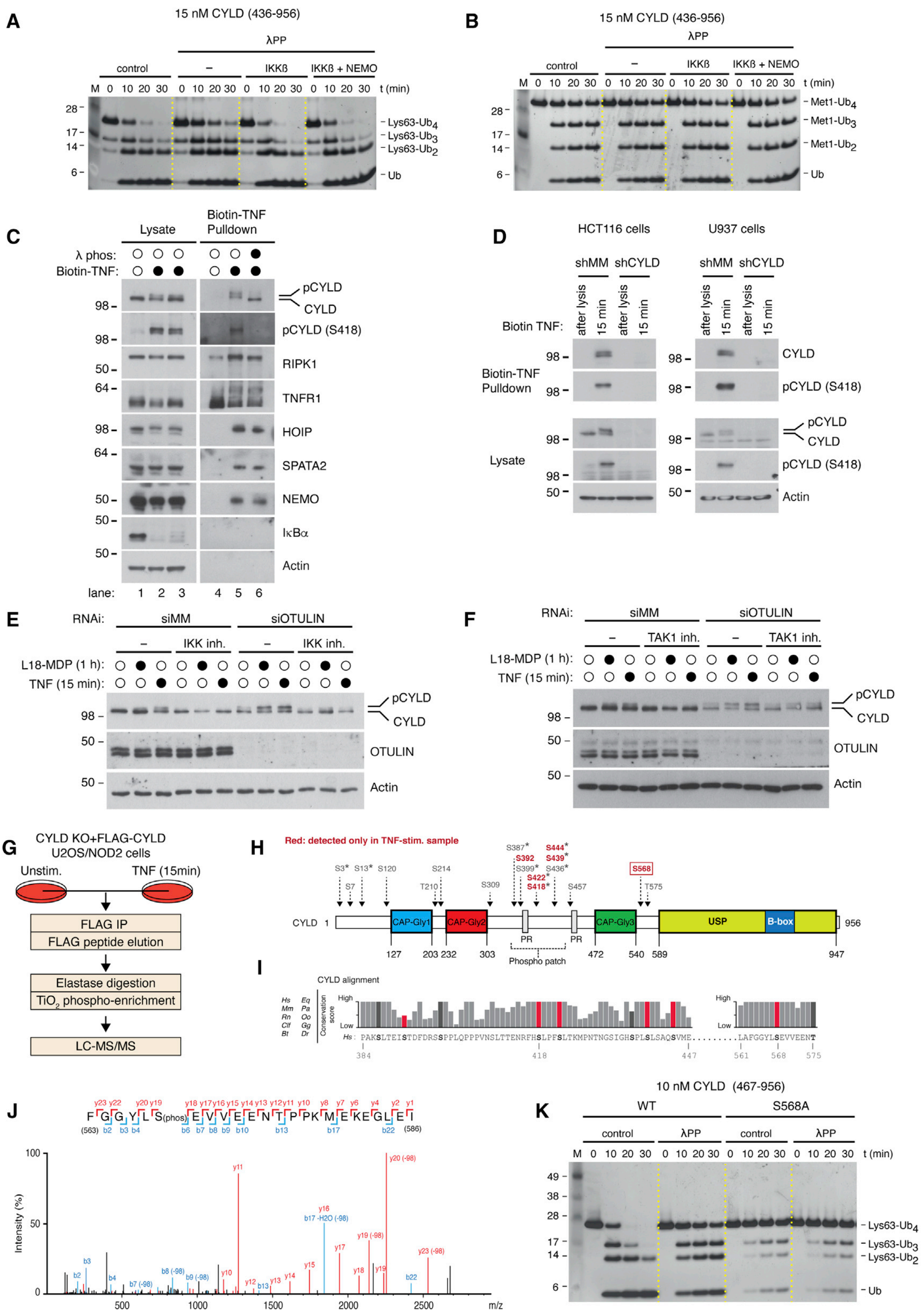

(legend on next page)

the catalytically inactive CYLD^{C601A} or the Ub-binding mutant CYLD^{L475P} remained hyper-responsive to NOD2 stimulation (Figures 3A–3C and S3B).

Mechanistically, CYLD trims RIPK2 ubiquitination after NOD2 stimulation and in the absence of CYLD, NOD2 stimulation leads to accumulation of ubiquitinated RIPK2 (Ub-RIPK2) species with a slower electrophoretic mobility than in CYLD-proficient cells (Elliott et al., 2016; Hrdinka et al., 2016). Ubiquitination of RIPK2 is mediated by multiple E3 Ub ligases, notably IAP proteins and LUBAC (Topal and Gyrd-Hansen, 2021). IAP antagonists (e.g., compound A [CpA]) that induce the degradation of cIAPs and also antagonize the interaction of XIAP with RIPK2 (Damgaard et al., 2013) efficiently prevent the accumulation of ubiquitinated RIPK2 in CYLD-proficient cells but fail to completely prevent RIPK2 ubiquitination in the absence of CYLD (Elliott et al., 2016; Hrdinka et al., 2016) (Figure 3D; compare lanes 1–6). These previous observations were used to assess the catalytic competence of CYLD variants in cells. Cells reconstituted with CYLD^{L475P} or CYLD^{C601A} displayed accumulation of Ub-RIPK2 species with a slower electrophoretic mobility similar to that observed in CYLD-deficient cells, and pre-treatment with CpA failed to block RIPK2 ubiquitination (Figure 3D; compare lanes 10–15). Taken together, this reveals functional importance for the Ub-binding interface in the CYLD CAP-Gly3 domain in regulating NOD2 signaling in U2OS/NOD2 cells.

CYLD is phosphorylated by IKK after TNFR1 and NOD2 stimulation

We next sought to understand CYLD regulation by phosphorylation in more detail. Phosphorylation of CYLD *in vitro* stimulated its Lys63-Ub DUB activity (Figures 1D and 1E), and IKK kinases are known to phosphorylate CYLD (Gringhuis et al., 2014; Hutti et al., 2009; Reiley et al., 2005). To test whether IKKβ accounts for CYLD regulation by phosphorylation *in vitro*, an intermediate-length CYLD variant (aa 436–956) with full activity, but lacking Ser418, was dephosphorylated by λPP. λPP was then removed, and CYLD was incubated with recombinant IKKβ in presence of Mg*ATP. Indeed, re-phosphorylation of CYLD by IKKβ selectively stimulated cleavage of Lys63-Ub (Figures 4A and 4B). NEMO/IKKγ, an adaptor of IKKα and IKKβ that also binds CYLD CAP-Gly3 (Saito et al., 2004), did not further stimulate CYLD DUB activity under these conditions (Figures 4A and 4B). We conclude that CYLD comprises a phospho-activity/specificity switch that can be activated by IKKβ and that turns it from a modestly active Met1- and Lys63-DUB to a highly active Lys63-DUB. This prompted us to assess IKKβ-dependent signaling cascades to uncover physiological phosphorylation sites and functional roles.

We hence investigated how stimulation of TNFR1 and NOD2 regulates phosphorylation of CYLD. Upon TNF treatment, CYLD in complex with SPATA2 is rapidly recruited to the TNF receptor signaling complex (TNF-RSC) by LUBAC (Draber et al., 2015; Elliott et al., 2016; Kupka et al., 2016; Schlicher et al., 2016; Wagner et al., 2016). Western blot analysis of the TNF-RSC purified with biotin-TNF showed that CYLD migrated as two distinct bands, consistent with the idea that CYLD is phosphorylated after TNF treatment (Reiley et al., 2005) (Figures 4C and 4D). Treatment of the biotin-TNF pull-down sample with λPP collapsed the double band of CYLD to a single band that migrated quicker than either band in the sample not treated with phosphatase, indicating extensive phosphorylation (Figure 4C; compare lanes 5 and 6). In line with this, a CYLD double band was detectable in lysates from TNF-treated cells, and phosphorylation of CYLD (pCYLD) at S418 was confirmed using a phospho-specific antibody (Figures 4C and 4D). pCYLD levels in lysates also increased after NOD2 stimulation (L18-MDP treatment), albeit less pronounced than following TNF treatment (Figure 4E; lanes 1–3). The phosphorylation of CYLD after TNF and L18-MDP treatment was blocked by pre-treatment with IKKβ inhibitors VII and XII or the TAK1 inhibitor oxozeaonol (Figures 4E and 4F; lanes 1–6), consistent with our *in vitro* data (Figures 4A and 4B) and previous reports (Reiley et al., 2005; Thein et al., 2014). Intriguingly, depletion of OTULIN increased the levels of pCYLD after TNF and L18-MDP treatment, and furthermore resulted in detectable pCYLD at steady state (Figures 4E and 4F). The pCYLD signal in OTULIN-depleted cells was blocked by IKKβ inhibitors but was only partially reduced by TAK1 inhibitors. The mechanism underlying this is interesting but is not explored further in this study. Taken together, these data suggest that CYLD is subject to extensive IKKβ-dependent phosphorylation following TNFR1 and NOD2 stimulation,

Figure 4. CYLD is phosphorylated at a novel Ser568 site as well as within the phospho-rich patch to activate its activity

(A) Qualitative DUB assay of CYLD (aa 436–956) purified from sf9 cells. CYLD was incubated in buffer alone, or with λPP as in Figures 1D and 1E. λPP was removed and dephosphorylated CYLD was either incubated with buffer alone (−), IKKβ, or the IKK complex (IKKβ + NEMO) prior to DUB assay using Lys63-Ub4 as substrate. For comparison, phosphorylated CYLD from sf9 cells was included (control).

(B) as in (A) but using Met1-Ub4 as substrate.

(C) Purification and western blot analysis of the TNFR1 complex in U2OS/NOD2 cell lines with subsequent λPP treatment. Cells were stimulated with biotin-TNF for 15 min (closed circles) or after lysis (open circles).

(D) Purification and western blot analysis of the TNFR1 complex in HCT116 and U937 cells expressing shRNA targeting CYLD or control shRNA. Cells were stimulated with biotin-TNF for the indicated time points.

(E and F) Western blot analysis of U2OS/NOD2 cells transfected with siRNA targeting OTULIN or mismatch control (shMM) and treated or not with IKK inhibitors VII + XII (E) or TAK1 inhibitor 5*Z*-7-oxozeaonol (F) for 30 min before stimulation with L18-MDP for 1 h or TNF for 15 min.

(G) Workflow for identification of CYLD phosphorylation sites by mass spectrometry.

(H) Schematic representation of identified CYLD phosphorylation sites.

(I) Analysis of evolutionary conservation of identified phosphorylation sites. TNF-induced sites are shown in red.

(J) Spectral data for identification of the novel S568 site in CYLD.

(K) Qualitative DUB assay with CYLD variants purified from sf9 cells. Samples were either incubated in buffer alone or in the presence of λPP prior to incubation with Lys63-Ub4 as substrate.

CellPress
OPEN ACCESS

although we cannot exclude that other kinases inhibited by the kinase inhibitors used in this study contribute to the phosphorylation of CYLD.

Phosphorylation of Ser568 stimulates CYLD Lys63-Ub DUB activity

To identify regulatory sites phosphorylated in response to TNF treatment, FLAG-tagged CYLD expressed in CYLD KO U2OS/NOD2 cells was purified from cells treated or not with TNF for 15 min (Figure S4A). Affinity-purified CYLD was subjected to elastase treatment, phospho-peptide enrichment, and liquid chromatography-tandem mass spectrometry (LC-MS/MS) (Figure 4G). This revealed six serine phosphorylation sites detected only in the TNF-treated samples. Five of these (Ser392, Ser418, Ser422, Ser439, Ser444) clustered within the "phospho-patch" located in the region between the second and third CAP-Gly domains, and four of the sites have been detected in other proteomic studies (Figure 4H; https://www.phosphosite.org/homeAction.action). We also identified a novel evolutionarily conserved site, Ser568, in the linker between CAP-Gly3 and the USP domain (Figures 4H and 4I). Four individual phosphopeptides were identified, and fragmentation analysis unequivocally determined phosphorylation of Ser568 (Figure 4J). To determine whether phosphorylation of Ser568 is the site within the linker region between CAP-Gly3 and the USP domain that regulates CYLD Lys63-Ub DUB activity, CYLD (aa 467–956) carrying a S568A mutation (CYLD^{S568A}) was purified from insect cells. Indeed, CYLD^{S568A} had substantially reduced Lys63-Ub DUB activity relative to CYLD^{WT} and was insensitive to λPP (Figure 4K), suggesting that phosphorylation of Ser568 functions as a switch to stimulate CYLD's Lys63-Ub DUB activity.

Phosphorylation is required for CYLD-dependent regulation of NOD2 signaling

To investigate how phosphorylation of CYLD affects its ability to regulate innate immune signaling, CYLD KO U2OS/NOD2 cells were stably reconstituted with CYLD mutants in which Ser568 and/or Ser418 were changed to alanine (CYLD^{S418A}, CYLD^{S568A}, CYLDS^{S418A,S568A}). The phosphorylation site mutant proteins were expressed at similar levels as CYLD^{WT}, indicating that the introduced point mutations did not affect protein stability (Figures S5A and S5B). Expression of the single-site mutants CYLD^{S418A} or CYLD^{S568A} restored L18-MDP-induced responses to almost the same level as CYLD^{WT} but, strikingly, the combined mutation of Ser418 and Ser568 (CYLD^{S418A,S568A}) interfered with the regulation of NOD2 inflammatory signaling to a similar extent as catalytically inactive CYLD^{C601A} and the Ub-binding mutant CYLD^{L475P} (Figures 3A–3C, 5A, and 5B). Accordingly, cells reconstituted with CYLD^{S418A,S568A} or CYLD^{C601A} displayed accumulation of Ub-RIPK2 species with a slower electrophoretic mobility similar to that observed in CYLD-deficient cells, and pre-treatment with CpA failed to block RIPK2 ubiquitination (Figure 5C). This was in contrast to cells reconstituted with CYLD^{WT} or CYLD^{S418A} in which ubiquitination of RIPK2 appeared similar to that observed in the parental cell line (Figure 5C). Cells reconstituted with CYLD^{S568A} displayed an intermediate deregulation of RIPK2 ubiquitination, which varied between experiments (Figures 5C and S5C). Taken together, this suggests that activation of CYLD's Lys63-Ub DUB activity through phosphorylation of residues Ser418 and Ser568 contributes to the regulation of NOD2 signaling. Intriguingly, in the context of IL-1β receptor signaling, CYLD did not play a significant role in regulating IRAK1 ubiquitination or downstream signaling events in reconstituted CYLD KO U2OS/NOD2 cells, suggesting that ubiquitin landscapes at different receptor complexes may be regulated through distinct mechanisms (Figures S5D–S5F).

Re-defining the role of CYLD activity at the TNF-RSC

In the context of TNF signaling, CYLD regulates ubiquitination of TNF-RSC components, including RIPK1 and TNFR1, to facilitate cell death signaling via RIPK1's kinase activity (Draber et al., 2015; Moquin et al., 2013; O'Donnell et al., 2011; Wei et al., 2017). However, the molecular role of CYLD's DUB activity at the TNF-RSC remains controversial (reviewed in Hrdinka and Gyrd-Hansen [2017]). Purification of the native TNF-RSC from different cell lines following a 15-min treatment with biotin-labeled TNF showed that knockout or depletion of CYLD resulted in reduced accumulation of ubiquitinated forms of TNFR1 and RIPK1, and reduced accumulation of Met1-Ub and Lys63-Ub at the TNF-RSC (Figures 6A, 6B, and S6A). In line with recent reports (Elliott et al., 2016; Wagner et al., 2016), this implies that CYLD as part of the TNF-RSC contributes to the assembly and/or stability of the complex. Indeed, detailed time course analysis of the TNF-RSC in CYLD KO cells reconstituted with CYLD^{WT} or CYLD^{C601A} showed that the catalytic activity of CYLD enhanced the ubiquitination of TNFR1 and trimmed Ub chains on RIPK1 as determined by a faster electrophoretic mobility of Ub-RIPK1 after 5 and 10 min of biotin-TNF stimulation (Figure 6C). Ub chain restriction analysis (UbiCRest) showed that TNFR1 is extensively modified by Met1-Ub whereas Ub-RIPK1 species consist predominantly of other linkages, including Lys63-Ub (Figure 6D) (Emmerich et al., 2016). It also revealed that the slow-migrating Ub-RIPK1 species that accumulated in the CYLD KO cells were sensitive to treatment with the Lys63-seletive DUB OTUD1 but not the Met1-Ub-specific DUB OTULIN. Corresponding to this, the catalytic activity of CYLD increased the amount of Met1-Ub at the TNF-RSC while trimming Lys63-Ub (Figure 6C). This coincided temporally with an increased recruitment and/or retention of LUBAC components after 5 and 10 min of biotin-TNF stimulation in cells reconstituted with CYLD^{WT} as compared to CYLD^{C601A} and CYLD KO cells (Figure 6C). Of note, the observed differences in LUBAC levels and RIPK1 ubiquitination at the TNF-RSC were not due to differences in the cellular level of LUBAC components and RIPK1 (Figure 6C). Taken together, these data suggest that CYLD at the TNF-RSC cleaves Lys63-Ub on RIPK1 while it promotes Met1-Ub-modification of TNFR1, likely by increasing the level of LUBAC within the TNF-RSC.

Phosphorylation is required for CYLD-dependent regulation of TNF signaling

Next, we asked how phosphorylation of CYLD on residues S418 and S568 affects its ability to regulate ubiquitination at the TNF-RSC. Reconstitution of cells with CYLD^{S418A,S568A} restored the levels of LUBAC, Met1-Ub, and Ub-TNFR1 in the TNF-RSC to similar levels as with CYLD^{WT} (Figures 7A, S7A, and S7B). In contrast, CYLD^{S418A,S568A} failed to fully restore trimming of

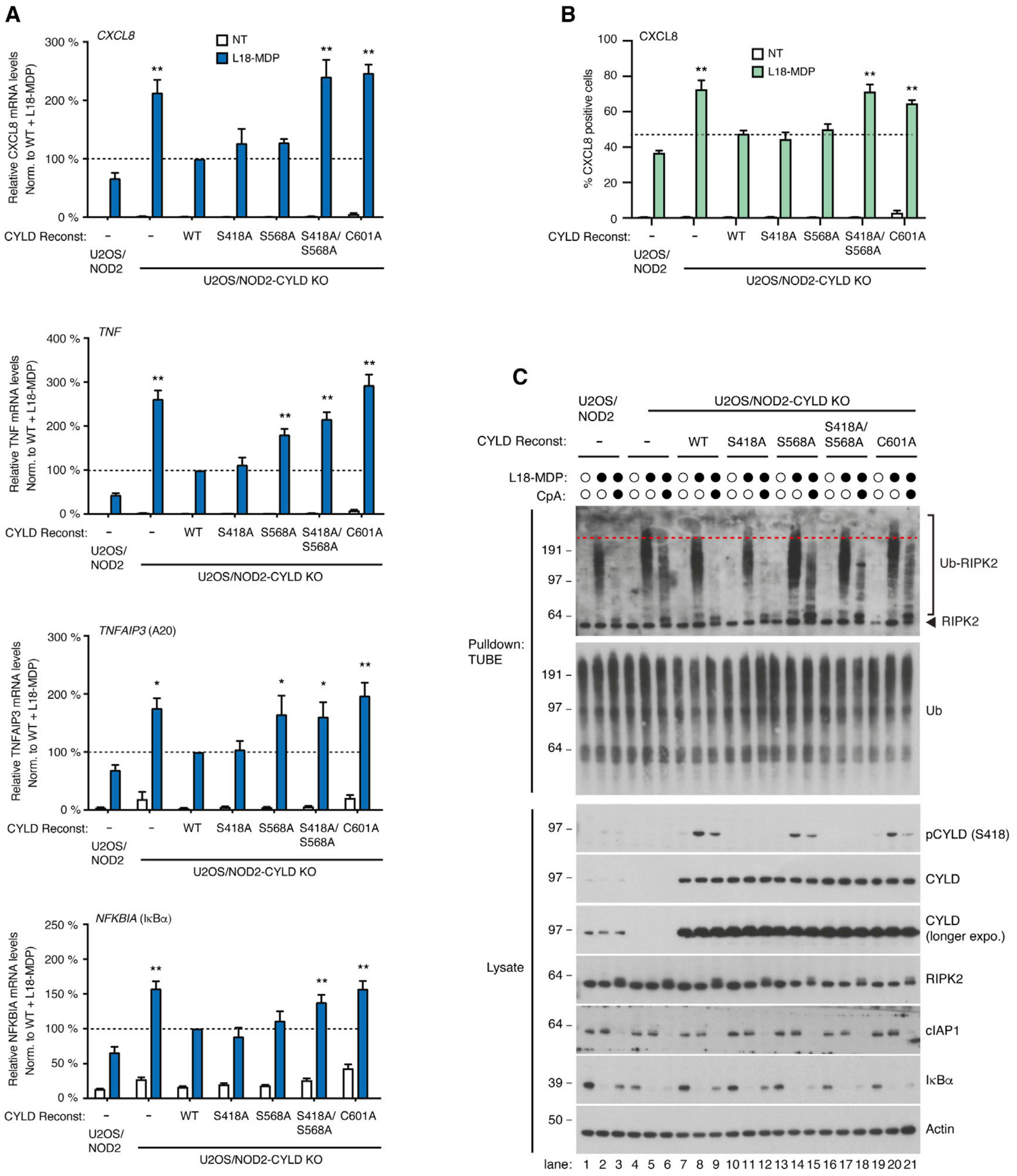

Figure 5. Ser568 phosphorylation together with Ser418 regulates CYLD's ability to control Ub deposition and signaling outcome

(A) Relative levels of NF-κB target gene transcripts in U2OS/NOD2 cell lines treated with L18-MDP for 3 h. Data shown represent mean ± SEM of six to eight independent experiments.

(B) Intracellular flow cytometry analysis of CXCL8 as in Figure 3B. Data shown represent mean ± SEM of six independent experiments.

(C) Purification and western blot analysis of Ub conjugates from U2OS/NOD2 cell lines treated for 1 h with CpA (1 μM) or DMSO prior to stimulation with L18-MDP for 1 h as indicated. *$p < 0.05$, **$p < 0.01$; n.s., not significant.

RIPK1-Ub whereas this was restored in cells reconstituted with $CYLD^{WT}$ (Figures 7A, S7A, and S7B). The effect on Lys63-Ub was less clear although there appeared to be slightly more Lys63-Ub at the TNF-RSC in $CYLD^{S418A,S568A}$ cells than in $CYLD^{WT}$ cells (Figures 7A, S7A, and S7B). Taken together, this suggests that activation of CYLD Lys63-Ub DUB activity by phosphorylation preferentially regulates Lys63-Ub and ubiquitination of RIPK1 whereas general CYLD activity contributes to the generation of Met1-Ub signals by facilitating recruitment and/or retention of LUBAC. We propose that the CYLD-SPATA2-LUBAC complex functions as a phosphorylation-regulated Ub-editing enzyme complex that trims Lys63-Ub and conjugates Met1-Ub to ensure appropriate signaling responses by innate immune receptors (Figure 7B).

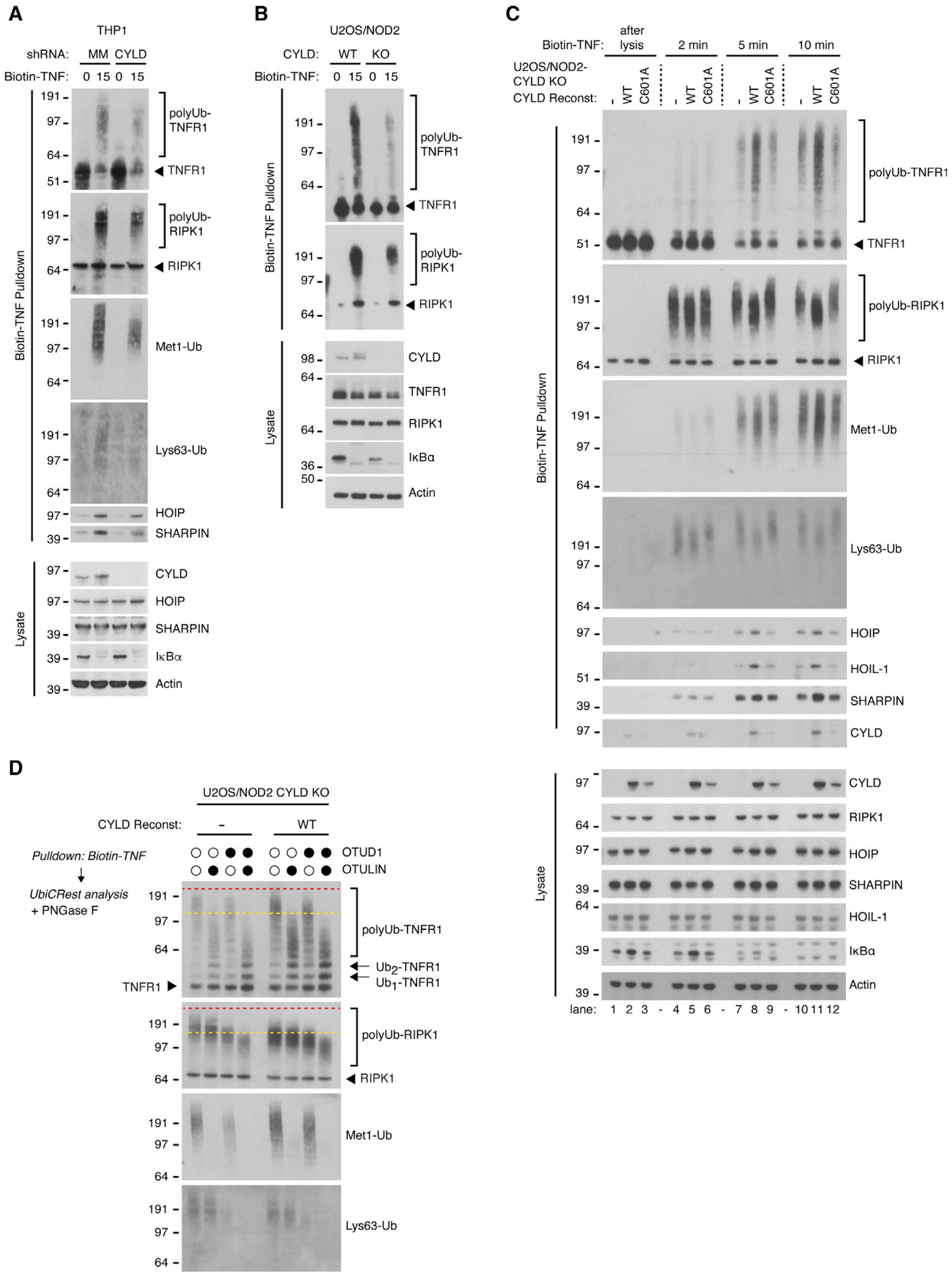

Figure 6. The absence of CYLD activity leads to reduced LUBAC retention at the TNF-RSC as well as increased Lys63-Ub and reduced Met1-Ub on receptor components

(A–C) Purification and western blot analysis of the TNFR1 complex in THP1 cells with shRNA knockdown of CYLD or mismatch control (A) and U2OS/NOD2 cell lines (B and C) stimulated with biotin-TNF as indicated.

(D) UbiCRest analysis of TNFR1 complexes purified as in (B). Samples were incubated or not with OTULIN and/or OTUD1 as indicated and analyzed by western blotting.

DISCUSSION

Structural and biochemical work in the last decade has characterized catalytic domains of DUBs, in particular enzymes of the USP and OTU family, in great detail. Such studies unveiled intriguing intrinsic linkage preferences, and in some cases pointed toward new cellular functions. However, a next layer of complexity, namely the regulation of DUB specificity and function by extra-catalytic domains and posttranslational modifications, has only emerged recently for a selected number of enzymes. Important examples include OTUD5/DUBA, which requires phosphorylation in its catalytic domain to become active (Huang et al., 2012), and OTUD4 that through phosphorylation switches from a low-activity Lys48-DUB to a highly active Lys63-DUB (Zhao et al., 2018). In this study, we add CYLD to the list of enzymes regulated in its activity and its specificity by extra-catalytic UBDs and phosphorylation events. Both regulatory mechanisms boost the hydrolysis of Lys63-Ub over Met1-Ub, effectively turning CYLD into a high-activity DUB that preferentially cleaves Lys63-Ub. This discovery is important, as to date, the preference of the isolated catalytic domain of CYLD for Met1-Ub, and its association with LUBAC via SPATA2, has led to the assumption that it regulates immune receptor signaling by cleaving Met1-Ub.

USP DUBs tend to be large and comprise extra-catalytic/exo domains that provide functional context. In CYLD, the three recognized CAP-Gly domains have been associated with microtubule binding (CAP-Gly1 and 2; Gao et al., 2008) and binding to the IKK complex via NEMO (CAP-Gly3; Saito et al., 2004). Indeed, the canonical role of the CAP-Gly domain is to bind microtubules via a defined surface (Yan et al., 2015). Through structural and biochemical methods, we herein reveal a new Ub-binding function for two of the three CAP-Gly domains in CYLD. Intriguingly, while both domains contact the canonical Ile44 patch, the Ub-binding interface on the CAP-Gly fold is distinct between CAP-Gly2 and CAP-Gly3. CAP-Gly3 uses a central Leu residue, Leu475, and an extended C terminus with hydrophobic residues to bind the Ub Ile44 patch. CAP-Gly2, however, uses different hydrophobic residues, including a uniquely exposed Phe residue, Phe288, that usually resides within the hydrophobic core of CAP-Gly domains. Despite this apparent surface fluidity and species conservation of both interfaces, the Ub-binding role of CAP-Gly domains seems to be restricted to CYLD.

Additionally, Ub-binding interfaces in either domain do not overlap with known binding sites for tubulin (CAP-Gly2) or NEMO (CAP-Gly3), and the interactions may co-exist or potentially even cooperate. In terms of their mechanistic contribution to CYLD activity and/or linkage preference, the CYLD UBDs appear to increase CYLD activity, primarily through increasing the binding of Ub chains to the enzyme, while also contributing to Lys63-linkage cleavage preference. Such mode of activity regulation is common among chain-processing DUBs, many of which contain additional UBDs (Mevissen and Komander, 2017).

Phosphorylation had been reported to be a regulatory mechanism of CYLD. Multiple Ser/Thr residues in a phospho-rich patch of CYLD located between CAP-Gly2 and CAP-Gly3 are found phosphorylated in cells (https://www.phosphosite.org/homeAction.action), creating a phosphorylation hotspot with a negative charge. Mutation of individual phosphorylation sites within this region did not measurably affect CYLD function, implying that it is the net overall charge of this region that affects CYLD function (Reiley et al., 2005). Some attention focused on CYLD Ser418, which, through mutational analysis, has been shown to be important for regulation of CYLD function by IKKε (Hutti et al., 2009). Contrary to this, we uncover that phosphorylation of the conserved Ser568 in the linker between the CAP-Gly3 and the USP domain is critical for activation of CYLD's Lys63-Ub DUB activity. The phospho-regulation of CYLD activity *in vitro* is dependent only on the linker region and requires Ser568, suggesting that the phosphorylation of this site increases the processing of Lys63-Ub by the CYLD USP domain. Further studies are required to determine mechanistically how phosphorylation regulates CYLD activity.

In cells, TNF stimulation induced the phosphorylation of several sites (Ser392, Ser418, Ser422, Ser439, Ser444) within the phospho-rich patch of CYLD in addition to Ser568, which was the only TNF-induced phosphorylation site detected outside this region. Mutation of Ser568 alone only had minor effects on CYLD-dependent regulation of TNFR1 and NOD2 signaling outcomes, but the combined mutation of Ser568 and Ser418 resulted in clear deregulation of these pathways, suggesting that phosphorylation of these residues cooperates for CYLD-dependent regulation of innate immune signaling. Whether phosphorylation of other sites within the phospho-rich patch have a similar role requires further investigation.

Within the NOD2-SC, CYLD trims Lys63- and Met1-Ub conjugated to RIPK2, but CYLD's role in regulating ubiquitination at the TNF-RSC remains less clear (Draber et al., 2015; Elliott et al., 2016; Hrdinka et al., 2016; Schlicher et al., 2016; Wagner et al., 2016). Our study suggests that the stimulation of CYLD's Lys63-Ub activity through phosphorylation of Ser568 and Ser418 is selectively important for trimming of RIPK1-Ub at the TNF-RSC at early time points after TNF treatment, but not for regulation of TNFR1-Ub or for Met1-Ub. This is consistent with the observation that RIPK1 is modified primarily by Lys63-Ub, whereas the TNFR1 is primarily modified with Met1-Ub (Emmerich et al., 2016) (Figure 6D). Paradoxically, the complete absence of CYLD activity led to reduced ubiquitination of TNFR1 and reduced Met1-Ub levels, which coincided with reduced levels of LUBAC components at the TNF-RSC. This indicates that CYLD activity facilitates the retention and/or recruitment of LUBAC to the TNF-RSC and thereby contributes to the deposition of Met1-Ub. To this end, studies of SPATA2, which is required for CYLD's recruitment to the TNF-RSC by LUBAC, similarly showed reduced ubiquitination of TNF-RSC components when SPATA2 is absent (Elliott et al., 2016; Wagner et al., 2016; Wei et al., 2017).

Auto-ubiquitination of LUBAC has been reported to prevent its recruitment to active signaling complexes, and this is controlled by OTULIN in unstimulated conditions (Elliott et al., 2014; Fiil et al., 2013; Heger et al., 2018). As OTULIN does not form part of the TNF-RSC, we speculate that the Met1-Ub DUB activity of CYLD may be important to deubiquitinate LUBAC within the TNF-RSC, facilitating its retention within the complex, and

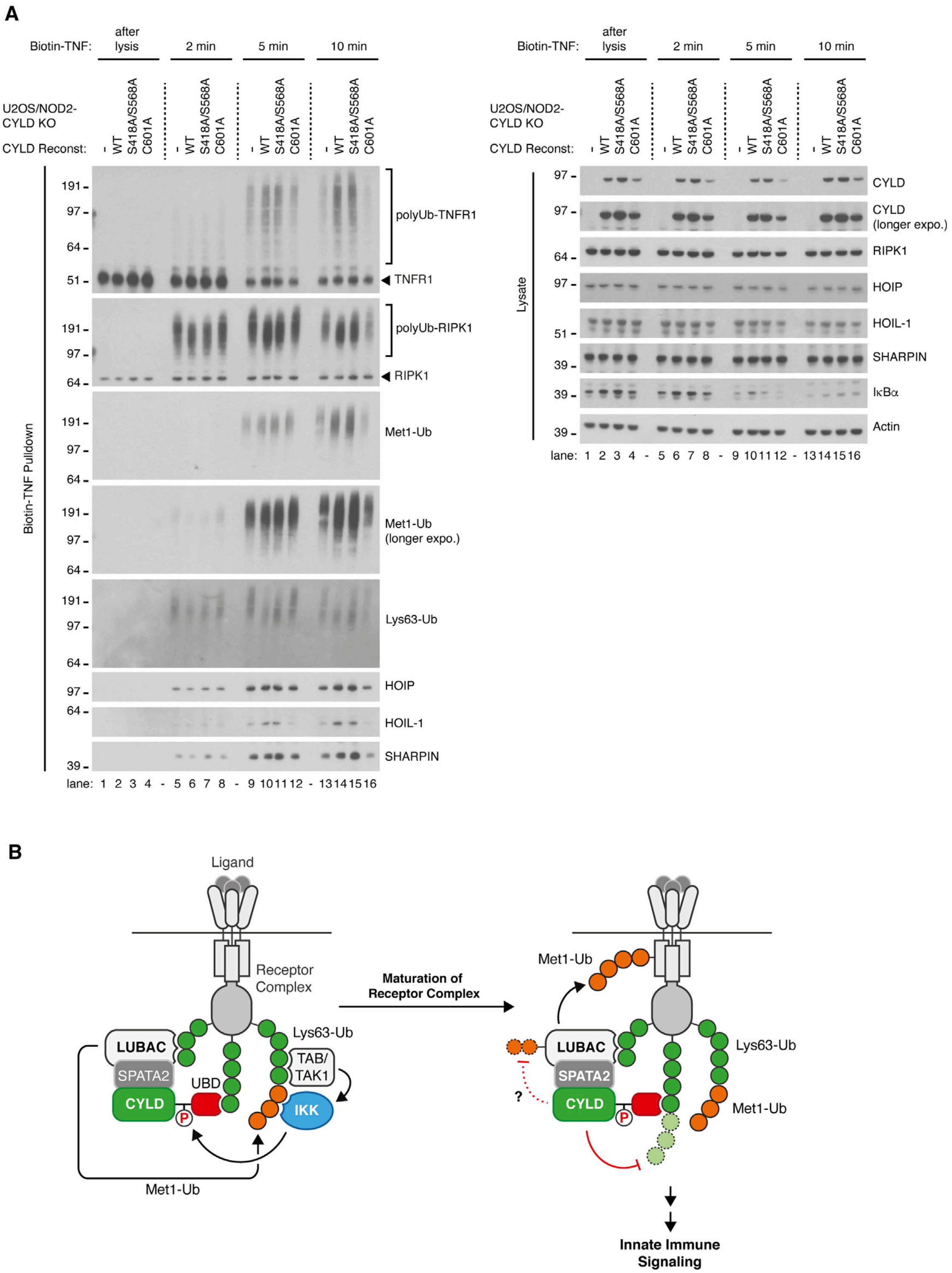

Figure 7. Non-phosphorylation-mediated activity of CYLD retains LUBAC at the TNF-RSC and restores normal Met1-Ub, but not Lys63-Ub, deposition

(A) Purification and western blot analysis of the TNFR1 complex in U2OS/NOD2 cell lines stimulated with biotin-TNF for the indicated time points.

(B) Schematic model of how Ub-binding via CAP-Gly3 and phosphorylation affects CYLD-regulation of Lys63- and Met1-Ub at receptor complexes.

thereby promoting the deposition of Met1-Ub to stabilize the complex.

In conclusion, our study uncovers a previously unknown reliance of extra-catalytic domains and phosphorylation in mediating CYLD's activity as primarily a Lys63-Ub DUB. Through investigation of the phosphorylation-mediated control of its function, we propose that the CYLD-SPATA2-LUBAC complex functions as a Ub-editing complex that hydrolyses Lys63-Ub and generates Met1-Ub on receptor components. In this complex, CYLD indirectly facilitates Met1-Ub deposition, possibly by preventing auto-ubiquitination of LUBAC within the TNF-RSC. This illustrates that CYLD contributes to regulation of immune signaling by mechanisms other than directly counteracting the activity of E3s within receptor signaling complexes.

Limitations of study

To investigate how Ub binding by CAP-Gly3 and phosphorylation of S568 affect CYLD's ability to regulate receptor signaling, we used CYLD KO U2OS/NOD2 cells in which CYLD variants were stably introduced by viral transduction. While this approach enabled us to directly compare the ability of several CYLD variants to regulate NOD2 and TNFR1 signaling events, the system also has clear limitations. First, the reintroduced CYLD variants were substantially overexpressed relative to the endogenous protein, which raises the possibility that the effects observed would not be as evident if the CYLD variants were expressed at endogenous levels. Second, in U2OS cells CYLD has a relatively modest effect on receptor-induced ubiquitination events and NF-κB signaling downstream of NOD2 and TNFR1 (Hrdinka et al., 2016). This made it challenging to investigate how phosphorylation of CYLD on S568 and S418 influenced ubiquitination at the TNF-RSC and signaling outcomes. Whereas we could demonstrate a clear alteration of RIPK1 and TNFR1 ubiquitination in different CYLD-deficient cells (Figures 6A–6C), the differences in RIPK1-Ub and Lys63-Ub at the TNF-RSC between $CYLD^{S418A,S568A}$ and $CYLD^{WT}$ cells are less obvious. To enable the reader to make an assessment of the experimental evidence, we have therefore included three independent repeats of the experiment (Figures 7A, S7A, and S7B).

We were unable to show that CYLD variants regulate TNFR1 signaling outcomes in U2OS cells, as CYLD plays a minor role in regulating NF-κB signaling in U2OS cells and the cells were largely insensitive to cell death induced by treatment with TNF in combination with caspase inhibitors and TAK1 inhibitors/IAP antagonists. We attempted to address the role of the CYLD variants in TNF-induced cell death in mouse embryonic fibroblasts (MEFs) and HT-29 cells. However, reintroduction of CYLD into CYLD KO MEFs or deletion of CYLD in HT-29 cells, in our hands, failed to reliably alter the sensitivity of the cells to TNF-induced cell death. Future studies using primary cells with knockin mutations of endogenous CYLD will be needed to elucidate the physiological importance of the CAP-Gly3 UBD and phosphorylation of S568 in CYLD identified in this study.

STAR★METHODS

Detailed methods are provided in the online version of this paper and include the following:

- KEY RESOURCES TABLE
- RESOURCE AVAILABILITY
  - Lead contact
  - Materials availability
  - Data and code availability
- EXPERIMENTAL MODEL AND SUBJECT DETAILS
  - Cell culture
- METHOD DETAILS
  - Antibodies
  - Generation of cell lines with stable expression of CYLD mutants
  - Transient and stable RNAi knockdown
  - Receptor stimulation
  - TNF pull-down
  - Purification of endogenous ubiquitin conjugates
  - UbiCRest analysis
  - Intracellular cytokine staining
  - RNA isolation, DNA synthesis, and qRT-PCR
  - Mass spectrometry for analysis of CYLD phosphorylation
  - Protein expression and purification
  - Crystallization data collection and refinement
  - Isothermal calorimetry (ITC)
  - Nuclear magnetic resonance spectroscopy
  - Qualitative DUB assays
- QUANTIFICATION AND STATISTICAL ANALYSIS

SUPPLEMENTAL INFORMATION

Supplemental information can be found online at https://doi.org/10.1016/j.celrep.2021.109777.

ACKNOWLEDGMENTS

This work was supported by the Ludwig Institute for Cancer Research Ltd. This research was funded in whole, or in part, by the Wellcome Trust (102894/Z/13/Z and 215612/Z/19/Z). For the purpose of open access, the author has applied a CC BY public copyright license to any author-accepted manuscript version arising from this submission. M.G.-H. is supported by the LEO Foundation. Work in the D.K. laboratory was supported by the Medical Research Council (U105192732), the European Research Council (724804), and the Lister Institute for Preventive Medicine. P.R.E. is supported by a Medical Research Council Career Development Award (MR/R008582/1). Access to DLS was supported in part by the EU FP7 infrastructure grant BIOSTRUCT-X (283570). We thank members of our groups for helpful suggestions and reading the manuscript.

AUTHOR CONTRIBUTIONS

M.G.-H., D.K., and P.R.E. conceived the study and wrote the manuscript. M.G.-H. supervised mammalian cell culture experiments. D.K., with support from P.R.E., supervised *in vitro* biochemical experiments and structural studies. P.R.E. and D.L. performed the experiments, analyzed the data, and wrote the manuscript. J.W., L.S., F.T., and B.M. performed the experiments and analyzed the data. G.B. and R.F. supervised and analyzed mass spectrometry experiments. S.M.V.F. supervised NMR experiments.

DECLARATION OF INTERESTS

D.K. is on the Scientific Advisory Board of BioTheryX, Inc. The remaining authors declare no competing interests.

CellPress
OPEN ACCESS

Received: June 18, 2021
Revised: August 25, 2021
Accepted: September 9, 2021
Published: October 5, 2021

REFERENCES

Adams, P.D., Afonine, P.V., Bunkóczi, G., Chen, V.B., Davis, I.W., Echols, N., Headd, J.J., Hung, L.W., Kapral, G.J., Grosse-Kunstleve, R.W., et al. (2010). PHENIX: A comprehensive Python-based system for macromolecular structure solution. Acta Crystallogr. D Biol. Crystallogr. *66*, 213–221.

Bignell, G.R., Warren, W., Seal, S., Takahashi, M., Rapley, E., Barfoot, R., Green, H., Brown, C., Biggs, P.J., Lakhani, S.R., et al. (2000). Identification of the familial cylindromatosis tumour-suppressor gene. Nat. Genet. *25*, 160–165.

Clague, M.J., Barsukov, I., Coulson, J.M., Liu, H., Rigden, D.J., and Urbé, S. (2013). Deubiquitylases from genes to organism. Physiol. Rev. *93*, 1289–1315.

Cummins, J.M., Kohli, M., Rago, C., Kinzler, K.W., Vogelstein, B., and Bunz, F. (2004). X-linked inhibitor of apoptosis protein (XIAP) is a nonredundant modulator of tumor necrosis factor-related apoptosis-inducing ligand (TRAIL)-mediated apoptosis in human cancer cells. Cancer Res. *64*, 3006–3008.

Damgaard, R.B., Fiil, B.K., Speckmann, C., Yabal, M., zur Stadt, U., Bekker-Jensen, S., Jost, P.J., Ehl, S., Mailand, N., and Gyrd-Hansen, M. (2013). Disease-causing mutations in the XIAP BIR2 domain impair NOD2-dependent immune signalling. EMBO Mol. Med. *5*, 1278–1295.

Damgaard, R.B., Walker, J.A., Marco-Casanova, P., Morgan, N.V., Titheradge, H.L., Elliott, P.R., McHale, D., Maher, E.R., McKenzie, A.N.J., and Komander, D. (2016). The deubiquitinase OTULIN is an essential negative regulator of inflammation and autoimmunity. Cell *166*, 1215–1230.e20.

Damgaard, R.B., Jolin, H.E., Allison, M.E.D., Davies, S.E., Titheradge, H.L., McKenzie, A.N.J., and Komander, D. (2020). OTULIN protects the liver against cell death, inflammation, fibrosis, and cancer. Cell Death Differ. *27*, 1457–1474.

Delaglio, F., Grzesiek, S., Vuister, G.W., Zhu, G., Pfeifer, J., and Bax, A. (1995). NMRPipe: A multidimensional spectral processing system based on UNIX pipes. J. Biomol. NMR *6*, 277–293.

Dominguez, C., Boelens, R., and Bonvin, A.M. (2003). HADDOCK: A protein-protein docking approach based on biochemical or biophysical information. J. Am. Chem. Soc. *125*, 1731–1737.

Draber, P., Kupka, S., Reichert, M., Draberova, H., Lafont, E., de Miguel, D., Spilgies, L., Surinova, S., Taraborrelli, L., Hartwig, T., et al. (2015). LUBAC-recruited CYLD and A20 regulate gene activation and cell death by exerting opposing effects on linear ubiquitin in signaling complexes. Cell Rep. *13*, 2258–2272.

Elliott, P.R. (2016). Molecular basis for specificity of the Met1-linked polyubiquitin signal. Biochem. Soc. Trans. *44*, 1581–1602.

Elliott, P.R., Nielsen, S.V., Marco-Casanova, P., Fiil, B.K., Keusekotten, K., Mailand, N., Freund, S.M., Gyrd-Hansen, M., and Komander, D. (2014). Molecular basis and regulation of OTULIN-LUBAC interaction. Mol. Cell *54*, 335–348.

Elliott, P.R., Leske, D., Hrdinka, M., Bagola, K., Fiil, B.K., McLaughlin, S.H., Wagstaff, J., Volkmar, N., Christianson, J.C., Kessler, B.M., et al. (2016). SPATA2 links CYLD to LUBAC, activates CYLD, and controls LUBAC signaling. Mol. Cell *63*, 990–1005.

Emmerich, C.H., Bakshi, S., Kelsall, I.R., Ortiz-Guerrero, J., Shpiro, N., and Cohen, P. (2016). Lys63/Met1-hybrid ubiquitin chains are commonly formed during the activation of innate immune signalling. Biochem. Biophys. Res. Commun. *474*, 452–461.

Emsley, P., Lohkamp, B., Scott, W.G., and Cowtan, K. (2010). Features and development of Coot. Acta Crystallogr. D Biol. Crystallogr. *66*, 486–501.

Evans, P.R., and Murshudov, G.N. (2013). How good are my data and what is the resolution? Acta Crystallogr. D Biol. Crystallogr. *69*, 1204–1214.

Fiil, B.K., Damgaard, R.B., Wagner, S.A., Keusekotten, K., Fritsch, M., Bekker-Jensen, S., Mailand, N., Choudhary, C., Komander, D., and Gyrd-Hansen, M. (2013). OTULIN restricts Met1-linked ubiquitination to control innate immune signaling. Mol. Cell *50*, 818–830.

Gao, J., Huo, L., Sun, X., Liu, M., Li, D., Dong, J.T., and Zhou, J. (2008). The tumor suppressor CYLD regulates microtubule dynamics and plays a role in cell migration. J. Biol. Chem. *283*, 8802–8809.

Gringhuis, S.I., Kaptein, T.M., Wevers, B.A., Mesman, A.W., and Geijtenbeek, T.B. (2014). Fucose-specific DC-SIGN signalling directs T helper cell type-2 responses via IKKε- and CYLD-dependent Bcl3 activation. Nat. Commun. *5*, 3898.

Harhaj, E.W., and Dixit, V.M. (2011). Deubiquitinases in the regulation of NF-κB signaling. Cell Res. *21*, 22–39.

He, Y., Hicke, L., and Radhakrishnan, I. (2007). Structural basis for ubiquitin recognition by SH3 domains. J. Mol. Biol. *373*, 190–196.

Heger, K., Wickliffe, K.E., Ndoja, A., Zhang, J., Murthy, A., Dugger, D.L., Maltzman, A., de Sousa E Melo, F., Hung, J., Zeng, Y., et al. (2018). OTULIN limits cell death and inflammation by deubiquitinating LUBAC. Nature *559*, 120–124.

Hjerpe, R., Aillet, F., Lopitz-Otsoa, F., Lang, V., England, P., and Rodriguez, M.S. (2009). Efficient protection and isolation of ubiquitylated proteins using tandem ubiquitin-binding entities. EMBO Rep. *10*, 1250–1258.

Hrdinka, M., and Gyrd-Hansen, M. (2017). The Met1-linked ubiquitin machinery: Emerging themes of (de)regulation. Mol. Cell *68*, 265–280.

Hrdinka, M., Fiil, B.K., Zucca, M., Leske, D., Bagola, K., Yabal, M., Elliott, P.R., Damgaard, R.B., Komander, D., Jost, P.J., and Gyrd-Hansen, M. (2016). CYLD limits Lys63- and Met1-linked ubiquitin at receptor complexes to regulate innate immune signaling. Cell Rep. *14*, 2846–2858.

Huang, O.W., Ma, X., Yin, J., Flinders, J., Maurer, T., Kayagaki, N., Phung, Q., Bosanac, I., Arnott, D., Dixit, V.M., et al. (2012). Phosphorylation-dependent activity of the deubiquitinase DUBA. Nat. Struct. Mol. Biol. *19*, 171–175.

Hutti, J.E., Shen, R.R., Abbott, D.W., Zhou, A.Y., Sprott, K.M., Asara, J.M., Hahn, W.C., and Cantley, L.C. (2009). Phosphorylation of the tumor suppressor CYLD by the breast cancer oncogene IKKε promotes cell transformation. Mol. Cell *34*, 461–472.

Kazimierczuk, K., and Orekhov, V.Y. (2011). Accelerated NMR spectroscopy by using compressed sensing. Angew. Chem. Int.Engl. *50*, 5556–5559.

Komander, D., Lord, C.J., Scheel, H., Swift, S., Hofmann, K., Ashworth, A., and Barford, D. (2008). The structure of the CYLD USP domain explains its specificity for Lys63-linked polyubiquitin and reveals a B box module. Mol. Cell *29*, 451–464.

Komander, D., Reyes-Turcu, F., Licchesi, J.D., Odenwaelder, P., Wilkinson, K.D., and Barford, D. (2009). Molecular discrimination of structurally equivalent Lys 63-linked and linear polyubiquitin chains. EMBO Rep. *10*, 466–473.

Kulathu, Y., and Komander, D. (2012). Atypical ubiquitylation—The unexplored world of polyubiquitin beyond Lys48 and Lys63 linkages. Nat. Rev. Mol. Cell Biol. *13*, 508–523.

Kupka, S., De Miguel, D., Draber, P., Martino, L., Surinova, S., Rittinger, K., and Walczak, H. (2016). SPATA2-mediated binding of CYLD to HOIP enables CYLD recruitment to signaling complexes. Cell Rep. *16*, 2271–2280.

Landau, M., Mayrose, I., Rosenberg, Y., Glaser, F., Martz, E., Pupko, T., and Ben-Tal, N. (2005). ConSurf 2005: The projection of evolutionary conservation scores of residues on protein structures. Nucleic Acids Res. *33*, W299–W302.

McCoy, A.J., Grosse-Kunstleve, R.W., Adams, P.D., Winn, M.D., Storoni, L.C., and Read, R.J. (2007). Phaser crystallographic software. J. Appl. Cryst. *40*, 658–674.

Mevissen, T.E.T., and Komander, D. (2017). Mechanisms of deubiquitinase specificity and regulation. Annu. Rev. Biochem. *86*, 159–192.

Montoya, J.F., Peral, J., and Salvador, P. (2011). Surface chemistry and interfacial charge-transfer mechanisms in photoinduced oxygen exchange at O_2-TiO_2 interfaces. ChemPhysChem *12*, 901–907.

Moquin, D.M., McQuade, T., and Chan, F.K. (2013). CYLD deubiquitinates RIP1 in the TNFα-induced necrosome to facilitate kinase activation and programmed necrosis. PLoS ONE *8*, e76841.

Morgenstern, J.P., and Land, H. (1990). Advanced mammalian gene transfer: High titre retroviral vectors with multiple drug selection markers and a complementary helper-free packaging cell line. Nucleic Acids Res. *18*, 3587–3596.

O'Donnell, M.A., Perez-Jimenez, E., Oberst, A., Ng, A., Massoumi, R., Xavier, R., Green, D.R., and Ting, A.T. (2011). Caspase 8 inhibits programmed necrosis by processing CYLD. Nat. Cell Biol. *13*, 1437–1442.

Ortega Roldan, J.L., Casares, S., Ringkjøbing Jensen, M., Cárdenes, N., Bravo, J., Blackledge, M., Azuaga, A.I., and van Nuland, N.A. (2013). Distinct ubiquitin binding modes exhibited by SH3 domains: Molecular determinants and functional implications. PLoS ONE *8*, e73018.

Raasi, S., Varadan, R., Fushman, D., and Pickart, C.M. (2005). Diverse polyubiquitin interaction properties of ubiquitin-associated domains. Nat. Struct. Mol. Biol. *12*, 708–714.

Reiley, W., Zhang, M., Wu, X., Granger, E., and Sun, S.C. (2005). Regulation of the deubiquitinating enzyme CYLD by IκB kinase gamma-dependent phosphorylation. Mol. Cell. Biol. *25*, 3886–3895.

Sahtoe, D.D., and Sixma, T.K. (2015). Layers of DUB regulation. Trends Biochem. Sci. *40*, 456–467.

Saito, K., Kigawa, T., Koshiba, S., Sato, K., Matsuo, Y., Sakamoto, A., Takagi, T., Shirouzu, M., Yabuki, T., Nunokawa, E., et al. (2004). The CAP-Gly domain of CYLD associates with the proline-rich sequence in NEMO/IKKγ. Structure *12*, 1719–1728.

Sato, Y., Goto, E., Shibata, Y., Kubota, Y., Yamagata, A., Goto-Ito, S., Kubota, K., Inoue, J., Takekawa, M., Tokunaga, F., and Fukai, S. (2015). Structures of CYLD USP with Met1- or Lys63-linked diubiquitin reveal mechanisms for dual specificity. Nat. Struct. Mol. Biol. *22*, 222–229.

Schaeffer, V., Akutsu, M., Olma, M.H., Gomes, L.C., Kawasaki, M., and Dikic, I. (2014). Binding of OTULIN to the PUB domain of HOIP controls NF-κB signaling. Mol. Cell *54*, 349–361.

Schlicher, L., Wissler, M., Preiss, F., Brauns-Schubert, P., Jakob, C., Dumit, V., Borner, C., Dengjel, J., and Maurer, U. (2016). SPATA2 promotes CYLD activity and regulates TNF-induced NF-κB signaling and cell death. EMBO Rep. *17*, 1485–1497.

Shen, Y., and Bax, A. (2015). Homology modeling of larger proteins guided by chemical shifts. Nat. Methods *12*, 747–750.

Shen, Y., Lange, O., Delaglio, F., Rossi, P., Aramini, J.M., Liu, G., Eletsky, A., Wu, Y., Singarapu, K.K., Lemak, A., et al. (2008). Consistent blind protein structure generation from NMR chemical shift data. Proc. Natl. Acad. Sci. USA *105*, 4685–4690.

Solyom, Z., Schwarten, M., Geist, L., Konrat, R., Willbold, D., and Brutscher, B. (2013). BEST-TROSY experiments for time-efficient sequential resonance assignment of large disordered proteins. J. Biomol. NMR *55*, 311–321.

Stamenova, S.D., French, M.E., He, Y., Francis, S.A., Kramer, Z.B., and Hicke, L. (2007). Ubiquitin binds to and regulates a subset of SH3 domains. Mol. Cell *25*, 273–284.

Takiuchi, T., Nakagawa, T., Tamiya, H., Fujita, H., Sasaki, Y., Saeki, Y., Takeda, H., Sawasaki, T., Buchberger, A., Kimura, T., et al. (2014). Suppression of LUBAC-mediated linear ubiquitination by a specific interaction between LUBAC and the deubiquitinases CYLD and OTULIN. Genes Cells *19*, 254–272.

Thein, S., Pham, A., Bayer, K.U., Tao-Cheng, J.H., and Dosemeci, A. (2014). IKK regulates the deubiquitinase CYLD at the postsynaptic density. Biochem. Biophys. Res. Commun. *450*, 550–554.

Topal, Y., and Gyrd-Hansen, M. (2021). RIPK2 NODs to XIAP and IBD. Semin. Cell Dev. Biol. *109*, 144–150.

van Zundert, G.C.P., Rodrigues, J.P.G.L.M., Trellet, M., Schmitz, C., Kastritis, P.L., Karaca, E., Melquiond, A.S.J., van Dijk, M., de Vries, S.J., and Bonvin, A.M.J.J. (2016). The HADDOCK2.2 web server: User-friendly integrative modeling of biomolecular complexes. J. Mol. Biol. *428*, 720–725.

Verboom, L., Martens, A., Priem, D., Hoste, E., Sze, M., Vikkula, H., Van Hove, L., Voet, S., Roels, J., Maelfait, J., et al. (2020). OTULIN prevents liver inflammation and hepatocellular carcinoma by inhibiting FADD- and RIPK1 kinase-mediated hepatocyte apoptosis. Cell Rep. *30*, 2237–2247.e6.

Vranken, W.F., Boucher, W., Stevens, T.J., Fogh, R.H., Pajon, A., Llinas, M., Ulrich, E.L., Markley, J.L., Ionides, J., and Laue, E.D. (2005). The CCPN data model for NMR spectroscopy: Development of a software pipeline. Proteins *59*, 687–696.

Wagner, S.A., Satpathy, S., Beli, P., and Choudhary, C. (2016). SPATA2 links CYLD to the TNF-α receptor signaling complex and modulates the receptor signaling outcomes. EMBO J. *35*, 1868–1884.

Wei, R., Xu, L.W., Liu, J., Li, Y., Zhang, P., Shan, B., Lu, X., Qian, L., Wu, Z., Dong, K., et al. (2017). SPATA2 regulates the activation of RIPK1 by modulating linear ubiquitination. Genes Dev. *31*, 1162–1176.

Wertz, I.E., Newton, K., Seshasayee, D., Kusam, S., Lam, C., Zhang, J., Popovych, N., Helgason, E., Schoeffler, A., Jeet, S., et al. (2015). Phosphorylation and linear ubiquitin direct A20 inhibition of inflammation. Nature *528*, 370–375.

Wickström, S.A., Masoumi, K.C., Khochbin, S., Fässler, R., and Massoumi, R. (2010). CYLD negatively regulates cell-cycle progression by inactivating HDAC6 and increasing the levels of acetylated tubulin. EMBO J. *29*, 131–144.

Winn, M.D., Ballard, C.C., Cowtan, K.D., Dodson, E.J., Emsley, P., Evans, P.R., Keegan, R.M., Krissinel, E.B., Leslie, A.G., McCoy, A., et al. (2011). Overview of the CCP4 suite and current developments. Acta Crystallogr. D Biol. Crystallogr. *67*, 235–242.

Winter, G., Waterman, D.G., Parkhurst, J.M., Brewster, A.S., Gildea, R.J., Gerstel, M., Fuentes-Montero, L., Vollmar, M., Michels-Clark, T., Young, I.D., et al. (2018). DIALS: Implementation and evaluation of a new integration package. Acta Crystallogr. D Struct. Biol. *74*, 85–97.

Yan, S., Guo, C., Hou, G., Zhang, H., Lu, X., Williams, J.C., and Polenova, T. (2015). Atomic-resolution structure of the CAP-Gly domain of dynactin on polymeric microtubules determined by magic angle spinning NMR spectroscopy. Proc. Natl. Acad. Sci. USA *112*, 14611–14616.

Zhao, Y., Mudge, M.C., Soll, J.M., Rodrigues, R.B., Byrum, A.K., Schwarzkopf, E.A., Bradstreet, T.R., Gygi, S.P., Edelson, B.T., and Mosammaparast, N. (2018). OTUD4 is a phospho-activated K63 deubiquitinase that regulates MyD88-dependent signaling. Mol. Cell *69*, 505–516.e5.

Zhou, Q., Wang, H., Schwartz, D.M., Stoffels, M., Park, Y.H., Zhang, Y., Yang, D., Demirkaya, E., Takeuchi, M., Tsai, W.L., et al. (2016). Loss-of-function mutations in TNFAIP3 leading to A20 haploinsufficiency cause an early-onset autoinflammatory disease. Nat. Genet. *48*, 67–73.

STAR★METHODS

KEY RESOURCES TABLE

REAGENT or RESOURCE	SOURCE	IDENTIFIER
Antibodies		
Anti-Actin, mouse monoclonal	Merck Millipore	Cat# MAB1501; RRID: AB_2223041
Anti-cIAP1, rat monoclonal	Enzo Life Sciences	Cat# ALX-803-335; RRID: AB_2227905
Anti-CYLD, mouse monoclonal	Santa Cruz Biotechnology	Cat# sc-74435; RRID: AB_1122022
Anti-pS418-CYLD, rabbit polyclonal	Cell Signaling Technology	Cat# 4500
Anti-ERK1/2, rabbit monoclonal	Cell Signaling Technology	Cat# 4695; RRID: AB_390779
Anti-phospho-ERK1/2, rabbit monoclonal	Cell Signaling Technology	Cat# 4370; RRID: AB_2315112
Anti-HOIL-1, mouse monoclonal	Santa Cruz Biotechnology	Cat# sc-365523; RRID: AB_10841591
Anti-HOIP, sheep polyclonal	R&D systems	Cat# AF8039; RRID: AB_2714038
Anti-IκBα, rabbit polyclonal	Cell Signaling Technology	Cat# 9242; RRID: AB_331623
Anti-IRAK1, rabbit polyclonal	Santa Cruz Biotechnology	Cat# sc-7883; RRID: AB_2233753
Anti-JNK, rabbit monoclonal	Cell Signaling Technology	Cat# 9258; RRID: AB_2141027
Anti-phospho-JNK, rabbit monoclonal	Cell Signaling Technology	Cat# 9251; RRID: AB_331659
Anti-OTULIN, rabbit polyclonal	Abcam	Cat# ab151117; RRID: AB_2728115
Anti-p38, rabbit polyclonal	Cell Signaling Technology	Cat# 9212; RRID: AB_330713
Anti-phospho-p38, rabbit monoclonal	Cell Signaling Technology	Cat# 4511; RRID: AB_2139682
Anti-RelA/p65, rabbit monoclonal	Cell Signaling Technology	Cat# 8242; RRID: AB_10859369
Anti-pS536-RelA/p65, rabbit monoclonal	Cell Signaling Technology	Cat# 3033; RRID: AB_331284
Anti-RIPK1, rabbit monoclonal	Cell Signaling Technology	Cat# 3493; RRID: AB_2305314
Anti-RIPK2, rabbit polyclonal	Santa Cruz Biotechnology	Cat# sc-22763; RRID: AB_2300888
Anti-SHARPIN, rabbit polyclonal	Proteintech	Cat# 14626-1-AP; RRID: AB_2187734
Anti-TNFR1, rabbit monoclonal	Cell Signaling Technology	Cat# 3736; RRID: AB_2241018
Anti-ubiquitin, mouse monoclonal	Imgenex	Cat# IMG-5021; RRID: AB_317568
Anti-ubiquitin (P4D1 clone), mouse monoclonal	Cell Signaling Technology	Cat# 3936; RRID: AB_331292
Anti-Lys63-linked ubiquitin, rabbit monoclonal	Merck Millipore	Cat# 05-1308; RRID: AB_1587580
Anti-Met1-linked ubiquitin, rabbit monoclonal	Merck Millipore	Cat# MABS199; RRID: AB_2576212
Anti-mouse-HRP, goat	Agilent (previously Dako)	Cat# P0447; RRID: AB_2617137
Anti-rat-HRP, goat	ThermoFisher	Cat# 31470; RRID: AB_228356
Anti-rabbit-HRP, goat	Bio-Rad	Cat# 1706515; RRID: AB_11125142
Anti-sheep-HRP, donkey	R&D systems	Cat# HAF016; RRID: AB_562591
Anti-CXCL8 (IL8)-APC, mouse	BioLegend	Cat# 511410; RRID: AB_893464
Anti-FLAG M2 affinity gel, mouse monoclonal	Sigma-Aldrich (now Merck Millipore)	Cat# A2220; RRID: AB_10063035
Bacterial and virus strains		
Rosetta II DE3	Novagen	Cat# 71400
DH10Bac	ThermoFisher	Cat# 10361012
Chemicals, peptides, and recombinant proteins		
L18-MDP	Invivogen	Cat# tlrl-lmdp
TNF	Peprotech	Cat# 300-01A
Biotin-TNF	R&D systems	Cat# BT210
FLAG-TNF	Enzo life systems	Cat# ALX-522-008-C050
IL-1β	Cell signaling Technologies	Cat# 2022
Brefeldin A	BioLegend	Cat# 420601
Monensin	BioLegend	Cat# 420701
ICC fixation buffer	BioLegend	Cat# 420801

(*Continued on next page*)

Continued

REAGENT or RESOURCE	SOURCE	IDENTIFIER
N-Ethylmaleimide (NEM)	Sigma Aldrich	Cat# E3876
cOmplete protease inhibitors	Roche	Cat# 04693159001
cOmplete Protease Inhibitor tablets	Sigma-Aldrich	Cat# 11873580001
PhosSTOP	Roche	Cat# 04906845001
lambda protein phosphatase	New England Biolabs and Gift from David Barford	Cat# P0773
3C Protease	Elliott et al., 2016; PMID: 27591049	N/A
SUMO Protease	Elliott et al., 2016; PMID: 27591049	N/A
Insect-XPRESS^{TM} Protein-free Insect Cell Medium	Lonza	Cat# BELN12-730Q
DNaseI	Sigma-Aldrich	Cat# DN25-100mg
Lysozyme	Sigma-Aldrich	Cat# L6876-5G
IPTG	VWR	Cat# 437144N
Tris-(2-carboxyethyl)phosphine (TCEP)	Fluorochem	Cat# M02624-10G
Ubiquitin	Elliott et al., 2016 PMID: 27591049	N/A
15N ammonium chloride	Sigma-Aldrich	Cat# 299251-25G
13C glucose	Sigma-Aldrich	Cat# 389374-10G
**Critical commercial assays**		
RevertAid RT Reverse transcriptase	Thermo Fisher Scientific	Cat# K1691
RiboLock RNase Inhibitor	Thermo Fisher Scientific	Cat# EO0381
RNAeasy mini kit	QIAGEN	Cat# 74104
Rnase-free Dnase set	QIAGEN	Cat# 79254
SYBR select Master Mix	Applied Biosystems	Cat# 4472908
PNGase F	New England Biolabs	Cat# P0704
**Deposited data**		
CYLD CAP-Gly3 (467-565): Ub complex	This study	PDB: 7OWC
CYLD CAP-Gly3 (467-552): Ub complex	This study	PDB: 7OWD
CYLD phosphorylation sites by Mass spectrometry	This study	PRIDE: PXD026791
**Experimental models: Cell lines**		
U2OS/FlpIn/Trex/HA-NOD2 (U2OS/NOD2)	Fiil et al., 2013; PMID: 23806334	N/A
U2OS/NOD2 CYLD KO (9E-8 clone)	Elliott et al., 2016; PMID: 27591049	N/A
U2OS/NOD2 CYLD KO + CYLD^{WT}	This study	N/A
U2OS/NOD2 CYLD KO + CYLD^{L475P}	This study	N/A
U2OS/NOD2 CYLD KO + CYLD^{S418A}	This study	N/A
U2OS/NOD2 CYLD KO + CYLD^{S568A}	This study	N/A
U2OS/NOD2 CYLD KO + CYLD$^{S418A, S568A}$	This study	N/A
U2OS/NOD2 CYLD KO + CYLD^{C601A}	This study	N/A
HCT-116	Cummins et al., 2004; PMID: 15126334	N/A
THP1	ATCC	Cat# TIB-202
U937	ATCC	Cat# CRL-1593.2
Sf9	ThermoFisher	Cat# 11496015
**Oligonucleotides**		
Listed in Table S2		
**Recombinant DNA**		
pBabe-puro	Morgenstern and Land, 1990; PMID: 2194165	RRID: Addgene_1764
pBabe-puro-CYLD^{WT}	This study	N/A
pBabe-puro-CYLD^{L475P}	This study	N/A

(*Continued on next page*)

Continued

REAGENT or RESOURCE	SOURCE	IDENTIFIER
pBabe-puro-CYLD^{C601A}	This study	N/A
pBabe-puro-CYLD^{S418A}	This study	N/A
pBabe-puro-CYLD^{S568A}	This study	N/A
pBabe-puro-CYLD^{S418A,S568A}	This study	N/A
pTRIP-Puro	Hrdinka et al., 2016; PMID: 26997266	N/A
pTRIP-Puro-shMM	Hrdinka et al., 2016; PMID: 26997266	N/A
pTRIP-Puro-shCYLD	Hrdinka et al., 2016; PMID: 26997266	N/A
pACEBAC-SUMO*	This study	N/A
pACEBAC-SUMO*-CYLD 583-956	This study	N/A
pACEBAC-SUMO*-CYLD 542-956	This study	N/A
pACEBAC-SUMO*-CYLD 467-956	This study	N/A
pACEBAC-SUMO*-CYLD 460-956	This study	N/A
pACEBAC-SUMO*-CYLD 1-956	This study	N/A
pOPINS-CYLD 120-213	This study	N/A
pOPINS-CYLD 226-313	This study	N/A
pOPINS-CYLD 460-582	This study	N/A
pOPINS-CYLD 467-582	This study	N/A
pOPINS-CYLD 467-582^{L475P}	This study	N/A
pOPINS-CYLD 226-313^{F288D}	This study	N/A
pOPINB-NEMO 383-419	This study	N/A
pOPINS-CLIP170 48-359	This study	N/A
pOPINS-CLIP170 48-147	This study	N/A
pOPINS-TBCB 151-244	This study	N/A
pOPINS-TBCE 1-110	This study	N/A
pOPINS-KIF13B 1685-1784	This study	N/A
pOPINS-DCTN1 1-107	This study	N/A
**Software and algorithms**		
GraphPad Prism	GraphPad	https://www.graphpad.com
Adobe Creative Suite	Adobe	https://www.adobe.com
Fiji (ImageJ)	https://www.nature.com/articles/nmeth.2019	https://imagej.net/software/fiji/
FlowJo	BD Biosciences	https://www.bdbiosciences.com/en-us/products/software/flowjo-v10-software
PEAKS Server v1.7	Bioinformatics Solutions Inc.	https://www.bioinfor.com/peaks-online/
Phenix	Adams et al., 2010; PMID: 20124702	https://www.phenix-online.org
COOT	Emsley et al., 2010; PMID: 20383002	https://www2.mrc-lmb.cam.ac.uk/Personal/pemsley/coot
CCP4 7.2	Winn et al., 2011; PMID: 21460441	https://www.ccp4.ac.uk/?page_id=1417
Pymol	Schrödinger, Inc	https://www.pymol.org/2/
Topspin 3.0	BRUKER	https://www.bruker.com/en/products-and-solutions/mr/nmr-software/topspin.html
NMRPipe	Delaglio et al., 1995; PMID: 8520220	https://www.ibbr.umd.edu/nmrpipe/index.html
Analysis 2.4	Vranken et al., 2005; PMID: 15815974	https://www.ccpn.ac.uk/v2-software/software/analysis

(*Continued on next page*)

Continued		
REAGENT or RESOURCE	SOURCE	IDENTIFIER
Pomana	Shen and Bax, 2015; PMID: 26053889	https://spin.niddk.nih.gov/bax/software/POMONA/
CS-Rosetta 3.7	Shen et al., 2008; PMID: 18326625	https://spin.niddk.nih.gov/bax/software/CSROSETTA/
Haddock 2.2	van Zundert et al., 2016; PMID: 26410586	https://www.bonvinlab.org/software/haddock2.2/
PEAQ Analysis	Malvern Instruments	https://www.malvernpanalytical.com/en/
Other		
Ni-NTA	QIAGEN	Cat# 30210
Resource Q	Cytiva Life Sciences	Cat# 17117901
HiLoad Superdex75	Cytiva Life Sciences	Cat# 28989333
HiLoad Superdex 200	Cytiva Life Sciences	Cat# 28989335

RESOURCE AVAILABILITY

Lead contact

Further information and requests for resources and reagents should be directed to and will be fulfilled by the lead contact, Mads Gyrd-Hansen (mgyrd@sund.ku.dk)

Materials availability

All reagents generated in this study will be made available by the lead contact upon request.

Data and code availability

- X-ray crystallography data and mass spectrometry data have been deposited at Protein Data Bank (PDB) and Proteomics Identification Database (PRIDE), respectively, and are publicly available as of the date of publication. Accession numbers are listed in the key resources table.
- This study does not report original code.
- Any additional information required to reanalyze the data reported in this paper is available from the lead contact upon request.

EXPERIMENTAL MODEL AND SUBJECT DETAILS

Cell culture

Human osteosarcoma U2OS cells (gender: female) were cultured in DMEM supplemented with 10% FBS and 60 μg/ml penicillin + 100 μg/ml streptomycin (1x PenStrep) in a humidified incubator at 37°C and 5% CO_2. U2OS/FlpIn/TRex/HA-NOD2 (U2OS/NOD2) cells were engineered in the Gyrd-Hansen lab from U2OS cells with the TRex^{TM} FlpIn system as described (Fiil et al., 2013). U2OS/NOD2 CYLD KO cells (9E-8 clone) are previously described (Elliott et al., 2016) and were used for reconstitution of CYLD expression (WT, S418A, S568A, S418A/S568A, C601, and L475P). Human monocytic THP1 cells (gender: male) and U937 cells (gender: male) were cultured in RPMI supplemented with 10% FBS, 1x PenStrep, 50 μM 2-mercaptoethanol, 1 mM Sodium pyruvate and GlutaMAX in a humidified incubator at 37°C and 5% CO2. Human colon carcinoma HCT-116 cells (gender: male) were cultured in McCoy's 5A (Modified) Medium, (GlutaMAX) supplemented with 10% FBS and 1x PenStrep in a humidified incubator at 37°C and 5% CO2. The cell lines used in this study have not been authenticated.

METHOD DETAILS

Antibodies

Antibodies were used according to the manufacturers' instructions and are listed in the key resources table.

Generation of cell lines with stable expression of CYLD mutants

U2OS/NOD2 CYLD KO cells were retrovirally transduced with CYLD-encoding virus particles. Full-length WT CYLD was cloned into the retroviral pBabe-puro vector (Morgenstern and Land, 1990). CYLD mutations (L475P, S418A, S568A, S418A/S568A, and C601) were generated by site-directed mutagenesis and were verified by sequencing. Retroviral particles were generated by transfection of

pBabe constructs (10 μg) into Phoenix-AMPHO cells in 10 cm plates and culturing for 3 days. Supernatant was collected and filtered through a 0.45 μm filter. Final concentrations of 5% w/v PEG-8000 and 150 mM NaCl were added to the supernatant and incubated with rotation at 4°C overnight. Virus particles were collected by centrifugation at 3500 g for 15 min and pellets resuspended in sterile PBS. U2OS/NOD2 CYLD KO cells were infected with the concentrated retroviral particles (1 – 4 μl) in the presence of 6-10 μg/ml polybrene (Sigma-Aldrich) over night and selected with 1 μg/ml puromycin (Invivogen) for one week.

Transient and stable RNAi knockdown

U2OS/NOD2 cells were seeded in 12-well plates and reverse transfected with a mix containing Lipofectamine RNAiMAX (Invitrogen Life Technologies) and siRNA oligonucleotides to a final concentration of 35 nM. After 72 h cells were stimulated as described. For stable knockdown of CYLD, the lentiviral vector pTRIP-Puro-shCYLD was used for delivery of shRNA targeting CYLD and pTRIP-Puro-shMM lentiviral vectors was used for delivery of non-targeting control shRNA (shMM) as previously described (Hrdinka et al., 2016). Hairpin sequences are listed in Key resources table. Lentiviral particles were generated in HEK293FT cells by co-transfection of shRNA vectors with packaging vectors psPAX2 and pMD.G. The virus-containing supernatants were harvested after 72 h, filtered through 0.45 μm filters, and concentrated by precipitation with PEG-8000. Cells were plated for infection in 6-well plates, infected with the concentrated lentiviral particles in the presence of 6-10 μg/ml polybrene overnight, and selected in the presence of 1 μg/ml puromycin until control cells had died.

Receptor stimulation

Cells were treated with the NOD2 ligand L18-MDP (InvivoGen) at 200 ng/ml, TNF (Peprotech) at 5 ng/ml, or IL-1β (GIBCO Life Technologies) at 10-20 ng/ml, added directly to the culture medium for the times indicated. Cells were lysed in the lysis buffer indicated for each methodology or TBS + 0.5% NP40 supplemented with 5 mM N-Ethylmaleimide (NEM; Sigma Aldrich), cOmplete protease inhibitor cocktail and PhosSTOP (Roche). Following stimulation, cells were processed as described.

TNF pull-down

Cells were stimulated with 50 ng/ml Biotin-TNF (R&D systems) for each indicated time point. For “0” time points, Biotin-TNF was added to cleared cell lysates. Two 15 cm plates of cells at 80% confluency were used for each condition. Media was replaced with 10 mL fresh media prior to lysis to remove unbound TNF. Cells were lysed in buffer containing 30 mM TrisHCl, 120 mM NaCl, 2 mM EDTA, 2 mM KCl, 1% Triton X-100 supplemented with 1 mM DTT, 5 mM N-Ethylmaleimide (NEM; Sigma Aldrich), cOmplete protease inhibitors and phosSTOP. Lysates were incubated on ice for 15 min and cleared by centrifugation. Lysate samples were collected and for ”0” time point samples, Biotin-TNF was added. Lysates were subsequently incubated with Streptavidin magnetic beads (Thermo Fisher Scientific; 88816) for at least 2 h at 4°C and washed five times in lysis buffer. Samples were eluted with 1x LSB for 5 min at 95°C. For phosphatase treatment, beads were resuspended and washed prior to LSB elution in 1x protein metallophosphatase (PMP) buffer (New England Biolabs) supplemented with $MnCl_2$ and incubated for 30 min at 30°C with lambda protein phosphatase (λPP, New England Biolabs). Samples were analyzed by SDS-PAGE and immunoblotting.

Purification of endogenous ubiquitin conjugates

Ub-conjugates were enriched using the Tandem Ubiquitin Binding Entity (TUBEs) methodology (Hjerpe et al., 2009) as previously described (Hrdinka et al., 2016). U2OS/NOD2 cells were seeded into 10 cm plates and treated at 80% confluency with 200 ng/ml L18-MDP for 1 h or 10 ng/ml IL-1β for 30 min. Where indicated, cells were treated with 1 μM Compound A (a kind gift from Tetralogic Therapeutics) for 1 h before receptor stimulation. Cells were lysed in TUBE lysis buffer (20 mM Na2HPO4, 20 mM NaH2PO4, 1% (v/v) NP-40, 2 mM EDTA) supplemented with 1 mM DTT, 5 mM N-Ethylmaleimide (NEM, Sigma Aldrich), cOmplete protease inhibitors (Roche), and PhosSTOP (Roche), incubated on ice for 15 min, and lysates were cleared by centrifugation. To purify ubiquitin conjugates, Glutathione Sepharose 4B beads (GE healthcare) were pre-bound for at least 1 h rotating at 4°C with GST-1xUBA^{ubq} (30 μg per condition) (Fiil et al., 2013) in TUBE lysis buffer and washed 1x with TUBE lysis buffer. Cleared lysates were added and incubated at 4°C overnight with rotation. Beads were washed four times with 500 μL of ice-cold PBS with 0.1% (v/v) Tween-20, and the bound material was eluted with 1x LSB for 5 min at 95°C. Samples were analyzed by SDS-PAGE and immunoblotting.

UbiCRest analysis

Biotin-TNF pull-down was performed as described above with Biotin-TNF stimulation for 15 min. After pull-down, beads were washed as described and then resuspended in DUB buffer (20 mM HEPES, 100 mM NaCl 1 mM MnCl2, 0.01% (w/v) Brij-35). DUBs were added: OTULIN (1 μM), OTUD1 (0.2 μM) or left without DUB as control and incubated for 30 min at 37°C. For deglycosylation of the TNFR1, PNGase F (New England Biolabs) was added according to manufacturer’s protocol into the DUB reaction volume. Bound material was eluted with 4xLSB to a final concentration of 1xLSB for 10 min at 70°C and analyzed by SDS-PAGE and immunoblotting.

Intracellular cytokine staining

U2OS/NOD2 cells (parental, CYLD KO, and CYLD KO reconstituted with CYLD mutants) were seeded in 12 well plates and stimulated for 5 h with 200 ng/ml L18-MDP in the presence of 5 μg/ml Brefeldin A (BioLegend) and 2 μM Monensin (BioLegend) protein transport

inhibitors. Following stimulation, cells were washed with PBS, disassociated with trypsin/EDTA (GIBCO Life Technologies) and fixed with ICC fixation buffer (BioLegend) O/N at 4°C. Cells were washed with PBS before being permeabilized with Perm/Wash buffer containing 2% (v/v) FCS, 0.1% (w/v) saponin, and 0.1% (w/v) NaN_3 in PBS and incubated in the Perm/Wash buffer with anti-IL8/APC (Biolegend) at a dilution of 100:1 for 1h at room temperature. Cells were washed with PBS and analyzed by FACS Canto Flow Cytometer (BD Biosciences) and data processed using FlowJo software (BD Biosciences).

RNA isolation, DNA synthesis, and qRT-PCR

Cells were plated in a 12 well plate and stimulated for 3 h with 200 ng/ml L18-MDP, 5 ng/ml TNF or 10 ng/ml IL-1β. RNA was isolated with RNeasy mini kit (QIAGEN) with on-column digestion of DNA performed alongside with the RNase-free DNase set (QIAGEN) according to the manufacturer's protocol. Total RNA was reverse transcribed with RevertAid RT Reverse transcriptase (Thermo Fisher Scientific) and a mixture of anchored oligo $(dT)_{20}$ primers with random pentadecamers in the presence of RiboLock RNase Inhibitor (Thermo Fisher Scientific). qPCR was performed with SYBR select Master Mix (Applied Biosystems). Gene-specific cDNA was amplified with the primer pairs listed in Key resources table.

Mass spectrometry for analysis of CYLD phosphorylation

For identification of phosphorylation sites in CYLD by LC-MS/MS, $2.5x10^8$ CYLD KO U2OS/NOD2 cells reconstituted with FLAG-CYLD were unstimulated or stimulated with TNF at 20 ng/ml for 15 mins. Cells were lysed in TBS + 0.5% NP40 supplemented with 5mM NEM, cOmplete protease inhibitor cocktail and PhosSTOP, and cleared by centrifugation. Inputs were taken and remaining lysate was incubated with FLAG (M2) affinity gel (Merck Millipore) for 2 h at 4°C. Samples were washed with TBS-T before elution with 100 μg/ml FLAG peptide for 15 mins. A fraction was removed for immunoblotting before preparation for LC-MS/MS: Samples were digested with Elastase, desalted using SepPak reversed phase columns and peptides were eluted with 1 M Glycolic acid in 80% Acetonitrile containing 5% TFA. Eluted peptides were phospho-enriched using 10 μM TiO_2 spin columns as described in (Montoya et al., 2011). The enriched peptides were dried and injected into an LC-MS/MS platform consisting of Dionex Ultimate 3000 nano LC and Orbitrap Fusion Lumos instruments. Sample separation was achieved with a 60 min gradient of 2%–35% acetonitrile in 0.1% formic acid and 5% DMSO and 250 nl/min flow rate with an EasySpray column (Thermo Fisher Scientific; ES803) of 50 cm length and 75 μm inner diameter. MS1 spectra were acquired with a resolution of 120.000 and an ion target of 400.000, followed by a top speed duty cycle of up to 3 s for MS/MS acquisition. Precursor ions were isolated in the quadrupole with a mass window of 1.6 Th and fragmented with HCD@28% normalized collision energy. MS/MS data was acquired in the ion trap with rapid scan mode and an ion target of 4000. LC-MS/MS data was analyzed using PEAKS Server V.7 (Bioinformatics Solutions) and a Uniprot/Trembl database. Variable modifications were set to Phosphorylation (S, T, Y), Oxidation (M) and Deamidation (N, Q). Mass tolerance was 10 ppm for precursor and 0.5 Da for fragment mass. The false discovery rate was set to 1% at peptide level.

Protein expression and purification

His6-SUMO*-3C-CYLD variants for protein expression from insect cells were cloned into a pACEBAC1-derived vector and transformed into DH10Bac cells (Invitrogen). Bacmids were transfected into sf9 cells to produce initial baculovirus, which was subsequently amplified and used to infects fresh sf9 cells. Cells were collected 72 hr post-infection. Cell pellets were resuspended in lysis buffer (20 mM Tris pH 8.5, 300 mM NaCl, 10 mM imidazole, 2 mM beta-mercaptoethanol, 5% (v/v) glycerol and 5 μM ZnCl2), supplemented with: cOmplete protease inhibitor (Roche), 1 mM PMSF, 0.2% (v/v) tween-20, 50 mM NaF and 10 mM glycerol-2-phosphate. Cells were lysed through manual homogenization and sonication and the resulatant supernatant clarified from centrifugation (18,000 rpm, 30 min) was applied to 1 mL Ni-NTA resin (QIAGEN). Resin was subsequently washed in lysis buffer and Ni-bound protein eluted in: 20 mM Tris pH 8.5, 300 mM NaCl, 2 mM beta-mercaptoethanol, 300 mM imidazole and 5% (v/v) glycerol. The His6-SUMO* tag was cleaved overnight at 4 C through addition of 3C protease while the protein was dialysed into anion exchange buffer: 20 mM Tris pH 8.5, 5% (v/v) glycerol, 4 mM DTT. Dilaysed samples were loaded onto a 6 mL ResourceQ anion exchange column (GE Healthcare) and eluted against a linear gradient of anion exchange buffer containing 1 M NaCl. CYLD-containing fractions were concentrated and further purified through size exclusion chromatography (HiLoad Superdex 200, GE Healthcare). The resultant fractions were judged to be 90%–95% pure following SDS-PAGE analysis and flash frozen. For dephosphorylation of CYLD from insect cells, lambda protein phosphatase (Gift from Dr David Barford, MRC-LMB) was incubated in the presence of 1 mM MnCl2 3 h prior to size exclusion chromatography.

His6-SUMO CAP-Gly constructs were expressed in Rosetta2 (DE3) pLacI cells. Cells were grown at 30°C in 2xTY medium supplemented with 30 μg/ml kanamycin and 34 μg/ml chloramphenicol to an OD_{600} of 0.6-1.0. The culture was cooled to 18°C prior to overnight induction with 400 μM IPTG. Cells were resuspended and lysed by sonication in lysis buffer (20 mM Tris pH 8.5, 300 mM NaCl, 50 mM imidazole, 2 mM β-mercaptoethanol, lysozyme, DNaseI (Sigma), 1 mM PMSF and cOmplete protease inhibitor cocktail (Roche). CAP-Gly proteins were purified by immobilised metal affinity chromatography using a HisTrap column (GE Healthcare). Pooled fractions were dialysed overnight into anion exchange buffer (20 mM Tris pH 8.5, 4 mM DTT). Proteins were purified by anion exchange chromatography using a ResourceQ column (GE Healthcare) and eluted in buffer containing: 20 mM Tris pH 8.5, 1 M NaCl, 4 mM DTT. Eluted fractions CAP-Gly domains were subjected to size exclusion chromatography (HiLoad 16/60 Superdex 75, GE Healthcare) in buffer containing: 20 mM Tris pH 7.4, 200 mM NaCl, 4 mM DTT. The resultant fractions were judged to be 95%–99% pure following SDS-PAGE analysis and flash frozen.

Crystallization data collection and refinement

CYLD CAP-Gly3 (465-552 and 465-565)–Ub crystals were grown by sitting-drop vapor diffusion with 1.1 molar excess of Ub. Crystals of CAP-Gly3 (aa 465-552)–Ub grew from conditions containing: 40 (v/v) PEG 300, 100 mM phosphate/citrate pH 4.2 and did not require transferring to a cryo-protecting solution prior to vitrification. Crystals of CAP-Gly3 (aa 465-565)–Ub grew from conditions containing: 35% (w/v) PEG 1,000, 50 mM HEPES pH 7.3 and were transferred to reservoir solution containing 25% (v/v) glycerol prior to cryo-cooling.

Diffraction data were collected at Diamond Light source beamline I03. Diffraction images were processed using DIALS (Winter et al., 2018) and manually scaled using AIMLESS (Evans and Murshudov, 2013) as part of the CCP4 software package (Winn et al., 2011). The structure of CYLD CAP-Gly3 bound to Ub was determined by molecular replacement using PHASER (McCoy et al., 2007) placing CAP-Gly3 and Ub (PDB IDs: 1ixd and 1ubq respectively). Iterative rounds of model building and refinement were performed with COOT (Emsley et al., 2010) and PHENIX (Adams et al., 2010), respectively. Data collection and refinement statistics can be found in Table S1. All structure figures were generated with Pymol (https://www.pymol.org/2/).

Isothermal calorimetry (ITC)

Prior to ITC experiments all samples were exchanged into ITC buffer: 20 mM HEPES pH 7.4, 200 mM NaCl, 2 mM TCEP. ITC data were performed using an Auto-iTC200 instrument (Malvern Instruments, Malvern, UK) at 25°C. 300 μM of CYLD CAP-Gly1-3 in the calorimeter cell were titrated against 4.5 mM Ub in the syringe using 19 injections of 2 μL preceded by a small 0.5 μl pre-injection. The changes in heat release were integrated over the entire titration and fitted to a single-site binding model using the PEAQ Analysis software (Malvern Instruments).

Nuclear magnetic resonance spectroscopy

His6-SUMO-tagged CYLD CAP-Gly2/3 and variants were expressed in 2M9 medium supplemented with ^{15}N NH_4Cl and for triple resonance experiments, ^{13}C glucose, and purified using standard HisTrap and anion exchange protocols described above. BEST-TROSY spectra used to monitor protein interactions (Solyom et al., 2013) and triple resonance assignment spectra were acquired at 298K on Bruker Avance III 600 MHz spectrometer equipped with a cryogenic triple TCI probe, unless otherwise stated. All samples were prepared with 5% v/v D_2O as a lock solvent in 20mM Tris pH 7.4, 100mM NaCl, 4 mM DTT. Backbone chemical shifts for the CAP-Gly3 domain were assigned using HNCO and HN(CA)CO; HNCA and HN(CO)CA experiments collected as pairs with 2048, 64 and 128 complex points in the proton, nitrogen and carbon dimensions respectively. The HNCACB and HN(CO)CACB experimental pair was collected with 2048. 64 and 110 complex points in the proton, nitrogen and carbon dimensions respectively. Backbone chemical shift assignments for the CAP-Gly2 domain were collected on an Bruker Avance II+ 700MHz spectrometer fitted with a TCI triple resonance cryoprobe using the same experimental set up as above, and supplemented with a HN(CA)NNH collected with 2048, 80 and 96 complex points in the proton and nitrogen dimensions respectively to provide N,N connections. All triple resonance experiments were recorded with Non Uniform Sampling (NUS) of between 14 and 50% to aid data acquisition times. Data processing and analysis were performed with Topspin3.0 (Bruker) and Analysis 2.4 (Vranken et al., 2005) with Data was processed using NMRPipe (Delaglio et al., 1995) including compressed sensing for NUS data reconstruction (Kazimierczuk and Orekhov, 2011)

Weighted chemical shift perturbations were calculated using the following equation:

$$\left(\delta H^2 + \left(\frac{\delta N}{5}\right)^2\right)^{0.5}$$

A model of CAP-Gly2 (226-313) was created using the backbone assignment and the homology-modeling webserver Pomona (Shen and Bax, 2015) that created input files for model calculation using Rosetta (Shen et al., 2008). Rosetta decoys were calculated in house using Rosetta version 3.7, with the best decoy determined by scoring the lowest Rosetta energy model with the backbone Ca RMSD using the scoring script included with the Pomona input files.

Complex models were created using the webserver Haddock2.2 (Dominguez et al., 2003; van Zundert et al., 2016). Protein residues with greater than 40% solvent accessibility were determined using Naccess2.2 and those residues that also had greater than the mean CSP as determined by NMR analysis were marked as active residues, with passive residues determined automatically by the Haddock server.

Qualitative DUB assays

Ub chain cleavage assays were performed as previously described (Elliott et al., 2016) using purified Met1- and Lys63 -linked tetra ubiquitin chains. CYLD variants were prepared at twice the desired enzyme concentration. In the case of IKKβ activation, CYLD pretreated with lambda protein phosphatase that was subsequently purified by size exclusion chromatography resulting in purified dephosphorylated CYLD, was incubated with 10 mM Mg-ATP with or without IKKβ for 30 min prior to the DUB assay. Reactions were performed at RT and the reaction was initiated through the addition of DUB to ubiquitin substrate. At designated time points, samples were taken and the reaction was quenched through addition of SDS sample buffer and analyzed by SDS-PAGE and stained using silver stain kit (Biorad).

QUANTIFICATION AND STATISTICAL ANALYSIS

Two-way ANOVA was used to determine the statistical significance of the qRT-PCR and flow cytometry data. The analysis was performed using GraphPad Prism (GraphPad Software Inc). Statistical details of experiments are described in the figure legends.

Cell Reports, Volume 37

Supplemental information

Regulation of CYLD activity

and specificity by phosphorylation

and ubiquitin-binding CAP-Gly domains

Paul R. Elliott, Derek Leske, Jane Wagstaff, Lisa Schlicher, Georgina Berridge, Sarah Maslen, Frederik Timmermann, Biao Ma, Roman Fischer, Stefan M.V. Freund, David Komander, and Mads Gyrd-Hansen

Table S1. Data Collection and Refinement Statistics, Related to Figure 2.

	CYLD 464-552 + Ub	CYLD 464-565 + Ub
**Data collection**		
Beamline	Diamond I03	Diamond I03
Space group	$P\ 4_12_12$	$P\ 2_12_12_1$
a, *b*, *c* (Å)	43.93, 42.93, 171.34	63.93, 64.49, 75.93
α, β, γ (°)	90.00, 90.00, 90.00	90.00, 90.00, 90.00
Wavelength	0.96863	0.96863
Resolution (Å)	41.64-1.71 (1.74-1.71)	64.49-1.85 (1.89-1.85)
R_{merge}	4.2 (59.1)	6.1 (56.6)
$< I / \sigma I >$	15.0 (1.9)	14.0 (2.1)
CC(1/2)	0.99 (0.90)	0.99 (0.75)
Completeness (%)	100.0 (99.9)	100.0 (100.0)
Redundancy	6.0 (6.3)	6.3 (6.3)
**Refinement**		
Resolution (Å)	41.64 - 1.71	49.15 - 1.85
No. reflections (test set)	18,237	27,435
R_{work} / R_{free}	21.0/23.8	18.3/23.6
No. atoms		
Protein	1342	2658
Water	98	192
B-factors		
Wilson B	34.76	29.47
Protein	49.9	40.6
Water	55.1	46.4
R.m.s deviations		
Bond lengths (Å)	0.011	0.009
Bond angles (°)	1.174	1.017
Ramachandran statistics (outliers, allowed, favoured)	0.6, 3.8, 95.6	0.0, 1.5, 98.5

Numbers in brackets are for the highest resolution bin.
Datasets were collected and structures determined from a single crystal each.

Figure S1

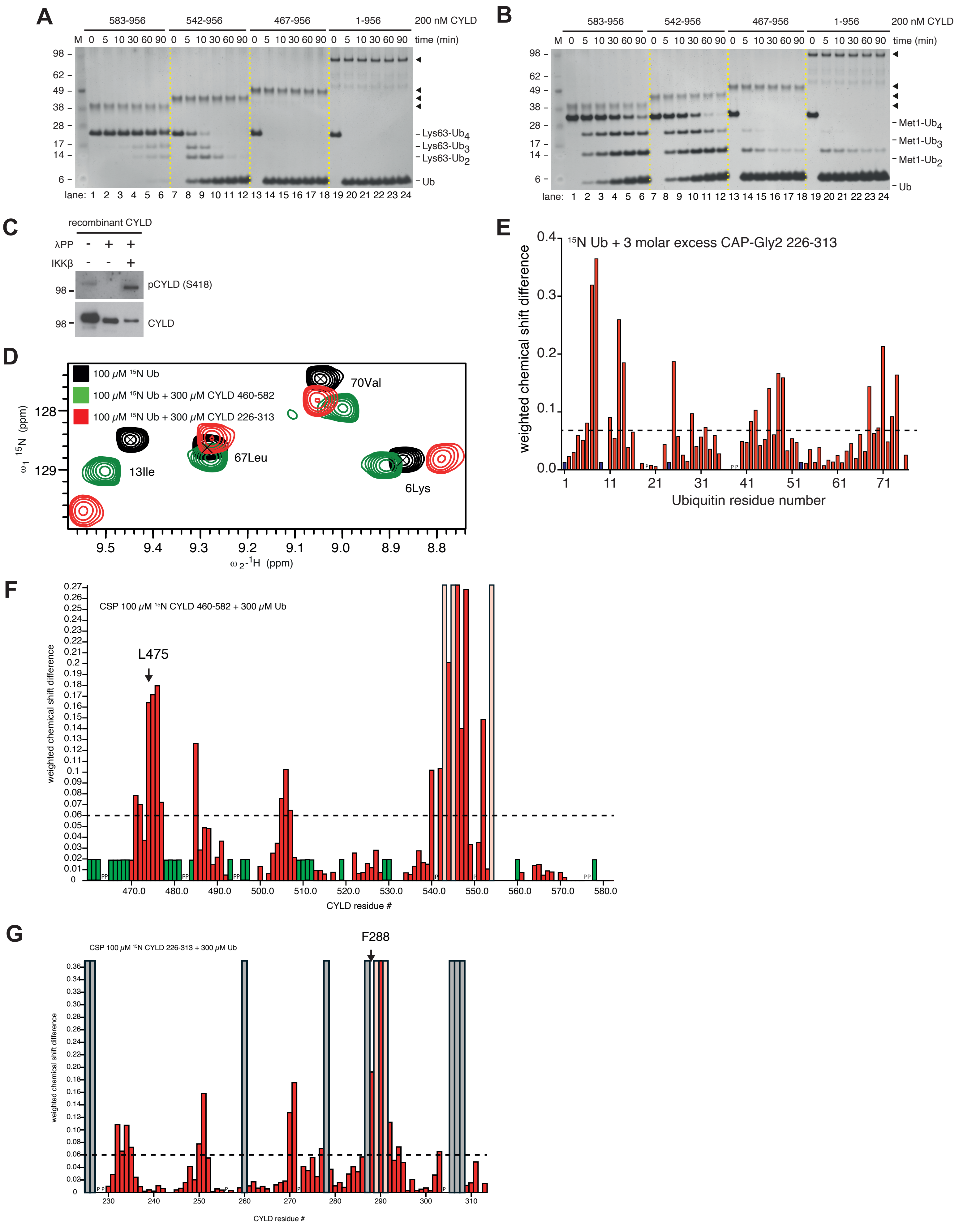

A
583-956
542-956
467-956
1-956
200 nM CYLD
time (min)
Lys63-Ub4
Lys63-Ub3
Lys63-Ub2
Ub
lane:
B
583-956
542-956
467-956
1-956
200 nM CYLD
time (min)
Met1-Ub4
Met1-Ub3
Met1-Ub2
Ub
lane:
C
recombinant CYLD
λPP
IKKβ
pCYLD (S418)
CYLD
D
100 μM 15N Ub
100 μM 15N Ub + 300 μM CYLD 460-582
100 μM 15N Ub + 300 μM CYLD 226-313
70Val
67Leu
13Ile
6Lys
ω1 15N (ppm)
ω2-1H (ppm)
E
15N Ub + 3 molar excess CAP-Gly2 226-313
weighted chemical shift difference
Ubiquitin residue number
F
CSP 100 μM 15N CYLD 460-582 + 300 μM Ub
L475
weighted chemical shift difference
CYLD residue #
G
CSP 100 μM 15N CYLD 226-313 + 300 μM Ub
F288
weighted chemical shift difference
CYLD residue #

Figure S1: IKKβ phosphorylates CYLD *in vitro*, Related to Figure 1.

(A) Qualitative DUB assays for assessing CYLD enzyme activity. Lys63-Ub4 was incubated with different CYLD fragments (200 nM) and Ub cleavage was monitored over 90 minutes. Samples were resolved by SDS-PAGE and stained using silver stain. (B) Qualitative DUB assays as in (A) but using Met1 Ub4 as a substrate. (C) To investigate phosphorylation of CYLD by IKKβ, full-length CYLD purified from sf9 cells was treated with λPP. The λPP was subsequently removed by size exclusion chromatography and the dephosphorylated CYLD was either incubated with buffer alone (-) or IKKβ. Phosphorylation status of CYLD was determined by western blotting as indicated. (D) BEST-TROSY region for ^{15}N-labelled Ub (**Extended Data 1**) collected alone or in the presence of three-molar excess CAP-Gly2 (226-313) or CAP-Gly3 (460-582). (E) Chemical shift perturbation plot of ^{15}N-labelled Ub titrated with three-fold excess of CAP-Gly2 (226-313). (F) Chemical shift perturbation plot of ^{15}N-labelled CAP-Gly3 titrated with three-fold excess of Ub. (G) Chemical shift perturbation plot as in (F) with ^{15}N-labelled CAP-Gly2.

Figure S2

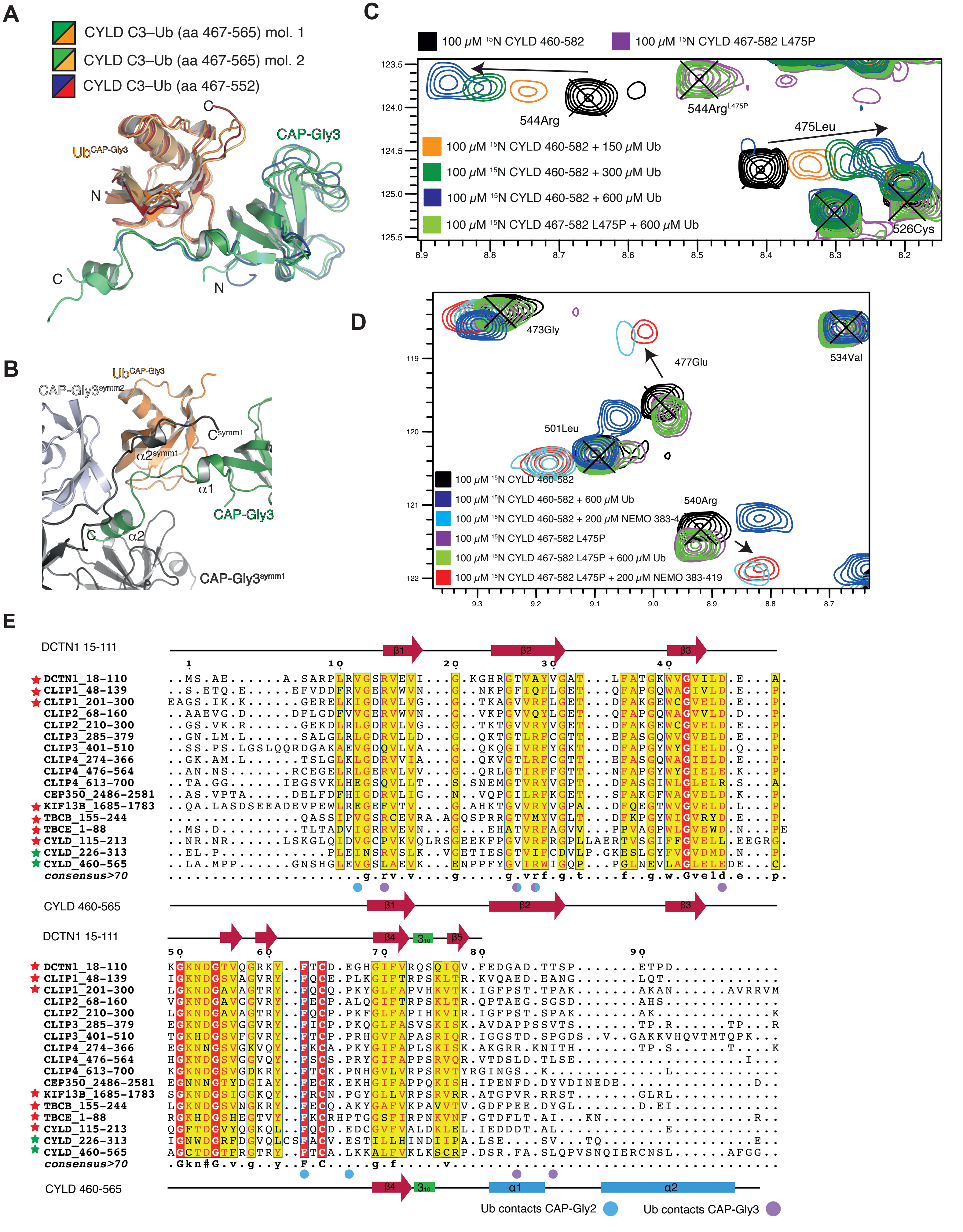

A
CYLD C3–Ub (aa 467-565) mol. 1
CYLD C3–Ub (aa 467-565) mol. 2
CYLD C3–Ub (aa 467-552)
Ub^CAP-Gly3
CAP-Gly3
C
N
B
Ub^CAP-Gly3
CAP-Gly3^symm2
C^symm1
α2^symm1
α1
CAP-Gly3
α2
CAP-Gly3^symm1
C
100 μM 15N CYLD 460-582
100 μM 15N CYLD 467-582 L475P
544Arg
544Arg^L475P
475Leu
526Cys
100 μM 15N CYLD 460-582 + 150 μM Ub
100 μM 15N CYLD 460-582 + 300 μM Ub
100 μM 15N CYLD 460-582 + 600 μM Ub
100 μM 15N CYLD 467-582 L475P + 600 μM Ub
D
473Gly
534Val
477Glu
501Leu
540Arg
100 μM 15N CYLD 460-582
100 μM 15N CYLD 460-582 + 600 μM Ub
100 μM 15N CYLD 460-582 + 200 μM NEMO 383-419
100 μM 15N CYLD 467-582 L475P
100 μM 15N CYLD 467-582 L475P + 600 μM Ub
100 μM 15N CYLD 467-582 L475P + 200 μM NEMO 383-419
E
DCTN1 15-111
DCTN1_18-110
CLIP1_48-139
CLIP1_201-300
CLIP2_68-160
CLIP2_210-300
CLIP3_285-379
CLIP3_401-510
CLIP4_274-366
CLIP4_476-564
CLIP4_613-700
CEP350_2486-2581
KIF13B_1685-1783
TBCB_155-244
TBCE_1-88
CYLD_115-213
CYLD_226-313
CYLD_460-565
consensus>70
CYLD 460-565
Ub contacts CAP-Gly2
Ub contacts CAP-Gly3

Figure S2: CAP-Gly domain 2 and 3 of CYLD bind Ub, Related to Figure 2.

(A) Superimposition of the two CAP-Gly3 molecules from crystal form 2 (467-565) (orthorhombic space group $P2_12_12_1$) and CAP-Gly3 (467-552) from crystal form 1 (tetragonal space group $P4_12_12$) onto one another, revealing low RMSD between the Ub orientation in all the structures obtained. (B) Crystal packing within crystal form 2 stabilizes helix $\alpha 2$ of CAP-Gly3 and positions Val551 against a separate interface of Ub. (C) BEST-TROSY region for ^{15}N-labelled CAP-Gly3 and the CAP-Gly3 Ub-binding mutant, L475P (**Extended Data 3-4**). The L475P mutation does not significantly alter the conformation of the CAP-Gly3 domain and is unable to bind Ub. (D) BEST-TROSY region for ^{15}N-labelled CAP-Gly3 and CAP-Gly3 L475P in the presence of six-fold molar excess of Ub or two-fold molar excess NEMO (383-419), revealing that mutation of the Ub binding interface does not affect NEMO binding. (E) Structure-based sequence alignment of all reported mammalian CAP-Gly domains. Secondary structure for DCTN1 CAP-Gly domain (18-110) is shown on top and CYLD CAP-Gly3 (460-565) is shown on the bottom. CAP-Gly2 and CAP-Gly3 residues that bind Ub are shown as blue and purple spheres respectively. CAP-Gly domains tested for Ub binding (**Extended Data 1**) that do not bind Ub are depicted as red stars, whereas CAP-Gly2 and CAP-Gly3 that bind Ub are represented as green stars.

Figure S3

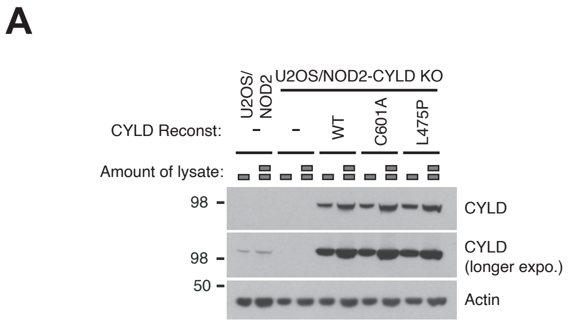

A
U2OS/NOD2
U2OS/NOD2-CYLD KO
CYLD Reconst:
–
–
WT
C601A
L475P
Amount of lysate:
98
98
50
CYLD
CYLD (longer expo.)
Actin

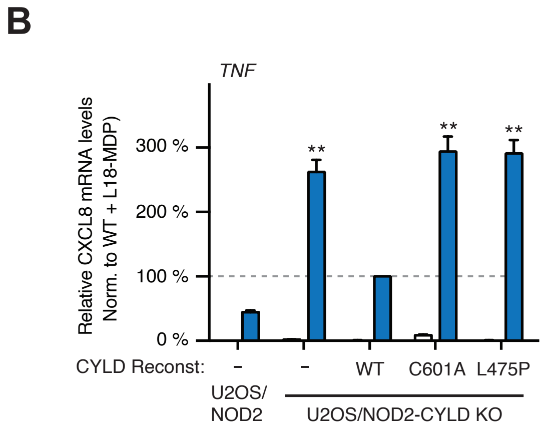

B
TNF
Relative CXCL8 mRNA levels
Norm. to WT + L18-MDP)
300 %
200 %
100 %
0 %
**
**
**
CYLD Reconst:
–
–
WT
C601A
L475P
U2OS/NOD2
U2OS/NOD2-CYLD KO

Figure S3: CAP-Gly3 of CYLD is required for regulation of Ub deposition and signaling outcome, Related to Figure 3.

(A) Western blot analysis of CYLD levels in U2OS/NOD2 CYLD KO cells with reintroduced CYLD mutants as indicated. (B) qRT-PCR of U2OS/NOD2 CYLD KO cell lines with reintroduced CYLD mutants to assess *TNF* mRNA levels. Cells were stimulated for 3 h with 200 ng/ml L18-MDP. Values were normalized to values from L18-MDP-treated CYLD KO cells reconstituted with $CYLD^{WT}$. Data shown represents mean ±SEM of at least 3 independent experiments.

Figure S4

A

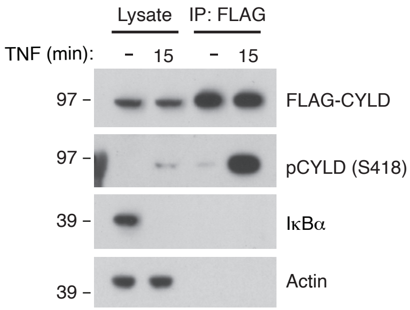

Lysate
IP: FLAG
TNF (min):
–
15
–
15
97 –
FLAG-CYLD
97 –
pCYLD (S418)
39 –
IκBα
39 –
Actin

Figure S4: TNF-induced phosphorylation of CYLD, Related to Figure 4.

(A) Immunoprecipitation of FLAG-tagged CYLD from lysates of U2OS/NOD2 CYLD KO cells expressing FLAG-CYLD^{WT} and stimulated or not with 20ng/ml TNF for 15 min. Lysates and IP samples were analyzed by western blotting as indicated before being processed for LC-MS/MS.

Figure S5

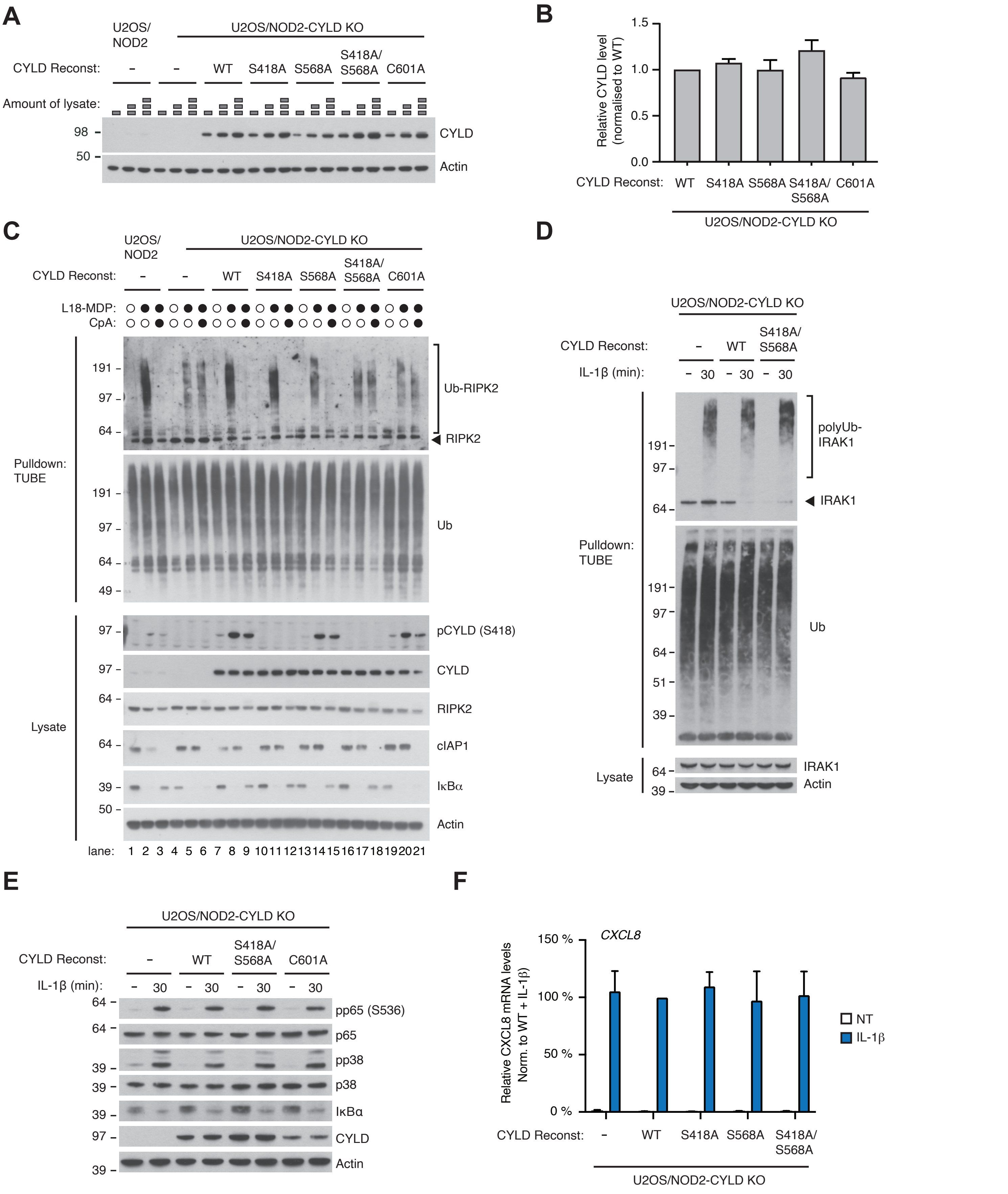
A
U2OS/NOD2
U2OS/NOD2-CYLD KO
CYLD Reconst: – – WT S418A S568A S418A/S568A C601A
Amount of lysate:
98
50
CYLD
Actin
B
Relative CYLD level (normalised to WT)
1.5
1.0
0.5
0.0
CYLD Reconst: WT S418A S568A S418A/S568A C601A
U2OS/NOD2-CYLD KO
C
U2OS/NOD2
U2OS/NOD2-CYLD KO
CYLD Reconst: – – WT S418A S568A S418A/S568A C601A
L18-MDP:
CpA:
191
97
64
Ub-RIPK2
RIPK2
Pulldown: TUBE
49
Ub
pCYLD (S418)
CYLD
RIPK2
cIAP1
IκBα
Actin
Lysate
39
50
lane: 1 2 3 4 5 6 7 8 9 10 11 12 13 14 15 16 17 18 19 20 21
D
U2OS/NOD2-CYLD KO
CYLD Reconst: – WT S418A/S568A
IL-1β (min): – 30 – 30 – 30
polyUb-IRAK1
IRAK1
Pulldown: TUBE
Ub
51
Lysate
IRAK1
Actin
E
U2OS/NOD2-CYLD KO
CYLD Reconst: – WT S418A/S568A C601A
IL-1β (min): – 30 – 30 – 30 – 30
pp65 (S536)
p65
pp38
p38
IκBα
CYLD
Actin
F
CXCL8
Relative CXCL8 mRNA levels Norm. to WT + IL-1β)
150 %
100 %
50 %
0 %
NT
IL-1β
CYLD Reconst: – WT S418A S568A S418A/S568A
U2OS/NOD2-CYLD KO

Figure S5: Ser568 phosphorylation together with Ser418 regulates CYLDs ability to control Ub deposition and signaling outcome after NOD2 but not IL-1β receptor stimulation, Related to Figure 5.

(A) Western blot analysis of CYLD levels in U2OS/NOD2 CYLD KO cells reconstituted with CYLD variants as indicated. (B) Densitometric quantification of CYLD levels in (A). Data shown represents mean ±SEM of 2 values. (C) Purification and western blot analysis of Ub conjugates from U2OS/NOD2 cell lines. Cells were treated for 1 h with CpA (1 μM) or DMSO prior to stimulation with 200 ng/ml L18-MDP for 1 h as indicated. Repeat experiment of data shown in Figure 5C to illustrate the partial reversal of RIPK2-Ub in cells expressing CYLD S568A. (D) Purification and western blot analysis of Ub conjugates from U2OS/NOD2 cell lines. Cells were treated for 30 min with 20 ng/ml IL-1β as indicated. (E) Western blot analysis to assess signaling after IL-1β treatment. Cells were treated with 10 ng/ml IL-1β for 30 min as indicated. (F) Relative levels of *CXCL8* mRNA in U2OS/NOD2 cell lines treated or not with 10 ng/ml IL-1β for 3 h. Data shown represents mean ±SEM of at least 3 independent experiments. $^*p < 0.05$, $^{**}p < 0.01$, *n.s.,* not significant.

Figure S6

A

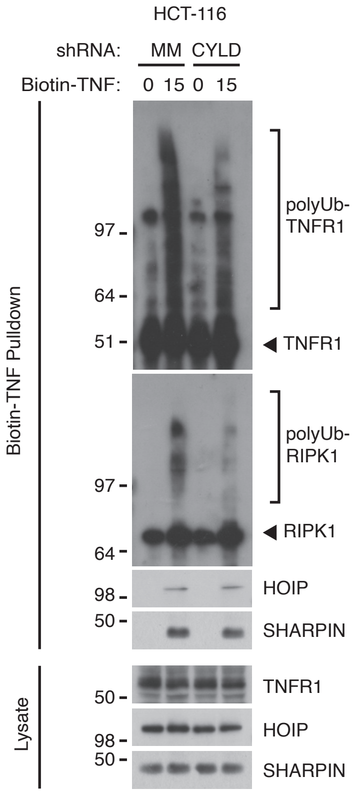
HCT-116
shRNA:
MM
CYLD
Biotin-TNF:
0
15
0
15
Biotin-TNF Pulldown
97
64
51
polyUb-
TNFR1
TNFR1
97
64
polyUb-
RIPK1
RIPK1
98
HOIP
50
SHARPIN
Lysate
50
TNFR1
98
HOIP
50
SHARPIN

Figure S6: The absence of CYLD activity interferes with ubiquitination of TNF-RSC components, Related to Figure 6.

(A) Purification and western blot analysis of the TNFR1 complex in HCT-116 cells with shRNA knockdown of CYLD or mismatch control. Cells were stimulated with 50 ng/ml Biotin-TNF for indicated time points. Samples used for western blots analysis are from the same experiment shown in Figure 4B.

Figure S7

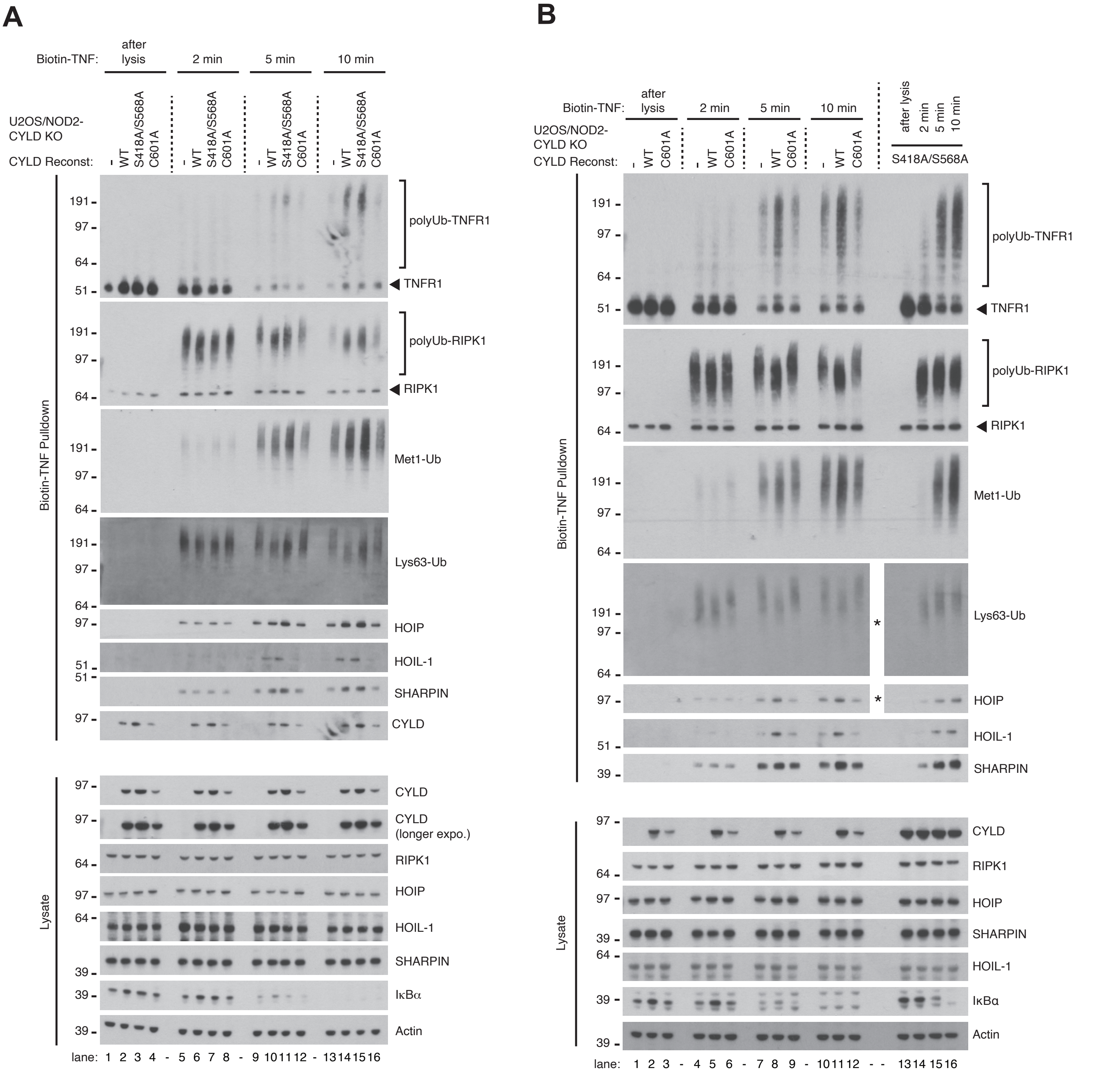
A
Biotin-TNF:
after lysis
2 min
5 min
10 min
U2OS/NOD2-CYLD KO
CYLD Reconst:
-
WT
S418A/S568A
C601A
Biotin-TNF Pulldown
polyUb-TNFR1
TNFR1
polyUb-RIPK1
RIPK1
Met1-Ub
Lys63-Ub
HOIP
HOIL-1
SHARPIN
CYLD
Lysate
CYLD
CYLD (longer expo.)
RIPK1
HOIP
HOIL-1
SHARPIN
IκBα
Actin
191
97
64
51
39
lane: 1 2 3 4 - 5 6 7 8 - 9 10 11 12 - 13 14 15 16
B
Biotin-TNF:
after lysis
2 min
5 min
10 min
U2OS/NOD2-CYLD KO
CYLD Reconst:
-
WT
C601A
S418A/S568A
Biotin-TNF Pulldown
polyUb-TNFR1
TNFR1
polyUb-RIPK1
RIPK1
Met1-Ub
Lys63-Ub
HOIP
HOIL-1
SHARPIN
*
Lysate
CYLD
RIPK1
HOIP
SHARPIN
HOIL-1
IκBα
Actin
191
97
64
51
39
lane: 1 2 3 - 4 5 6 - 7 8 9 - 10 11 12 - - 13 14 15 16

Figure S7: Non-phosphorylation-mediated activity of CYLD retains LUBAC at the TNF-RSC and restores normal Met1-Ub, but not Lys63-Ub, deposition, **Related to Figure 7.**

(A and B) Independent repeats of the data shown in Figure 7A. Purification and western blot analysis of the TNFR1 complex in U2OS/NOD2 cell lines as indicated. Cells were stimulated with 50 ng/ml Biotin-TNF for indicated time points. Lanes 1-12 in (B) are also shown in Figure 6C. Asterisks in (B) indicate that the scanned blots for Lys63-Ub and HOIP have been cut and reassembled to align with other blots in the figure. Due to a loading error the $CYLD^{S418A/S586A}$ samples (lanes 13-16) were shifted one lane relative to the other blots.

Extended data 1

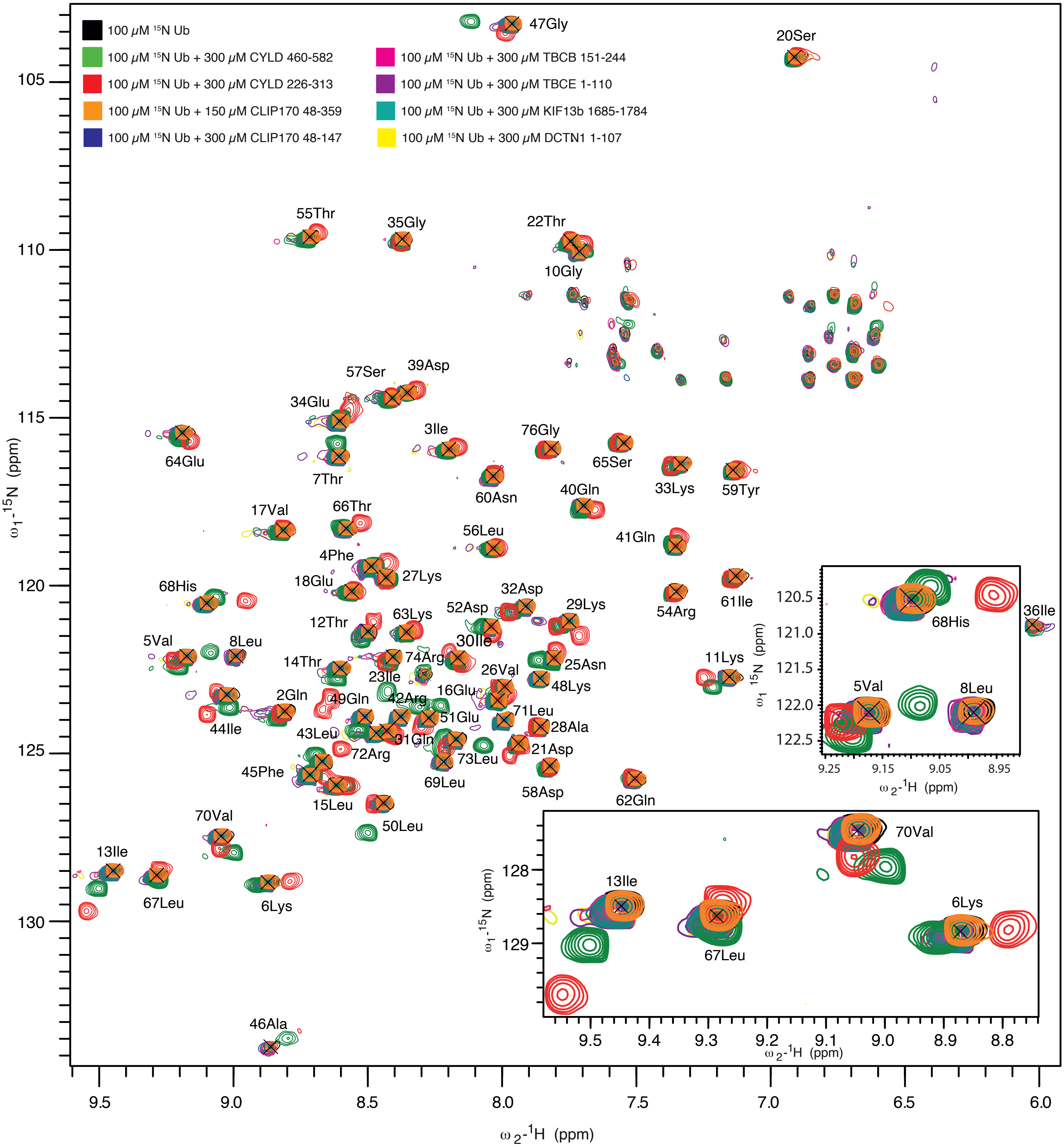

Extended Data 1. Map of chemical shift perturbations, Related to Figure 1.

BEST-TROSY spectra for ^{15}N-labelled Ub alone (black) or in the presence of three-fold molar excess CAP-Gly domains. Chemical shift perturbations are only observed for CYLD CAP-Gly2 and CAP-Gly3 domains, green and red respectively. Residue numbers correspond to resonance assignment of Ub are shown.

Extended data 2

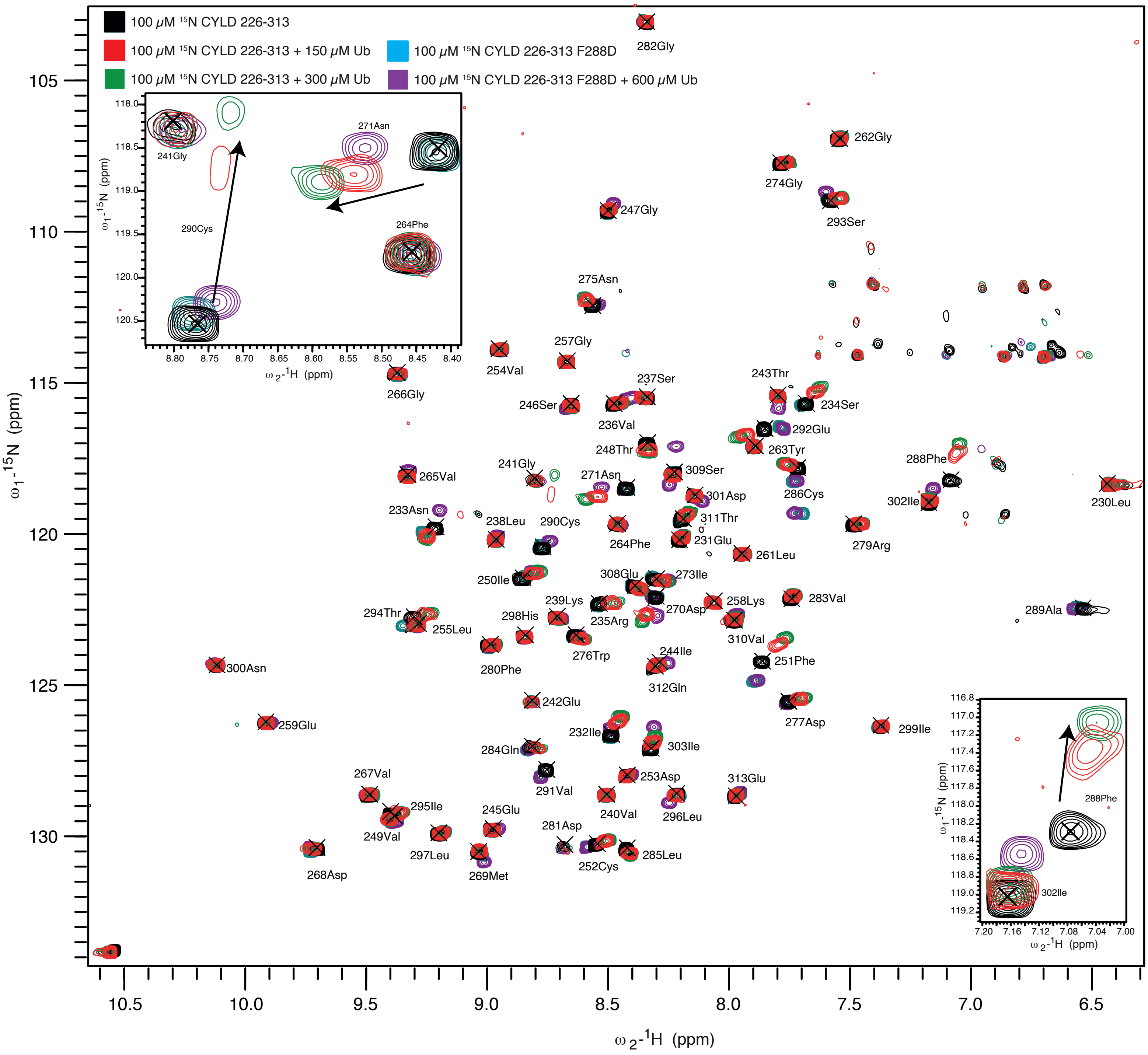

Extended Data 2. Map of chemical shift perturbations, Related to Figure 2.

BEST-TROSY spectra for ^{15}N-labelled CAP-Gly2 (black) or CAP-Gly2 F288D (blue) alone or in the presence of increasing concentrations of Ub. Complete backbone assignment of ^{13}C, ^{15}N CAP-Gly2 resonances permitted detailed interactions between CAP-Gly2 and Ub to be observed. Insert two regions show detailed titration of resonances Cys290 and Phe288.

Extended data 3

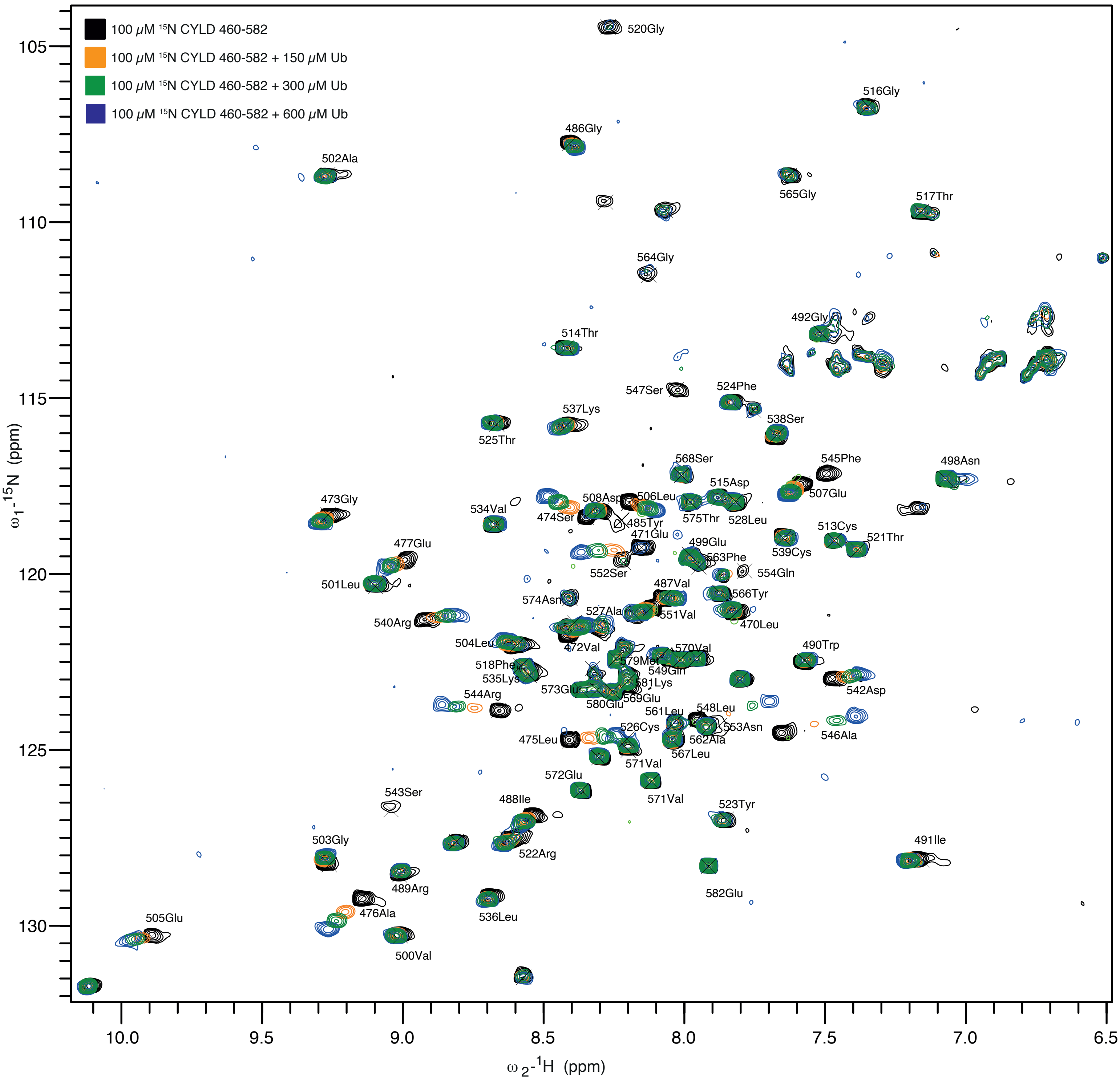

Extended Data 3. Map of chemical shift perturbations, Related to Figure 2.

BEST-TROSY spectra for ^{15}N-labelled CAP-Gly3 (black) with increasing concentrations of Ub. Resonance positions for CAP-Gly3 amino acids derived from complete backbone assignment of ^{13}C, ^{15}N CAP-Gly3 (460-582) spectra are shown.

Extended data 4

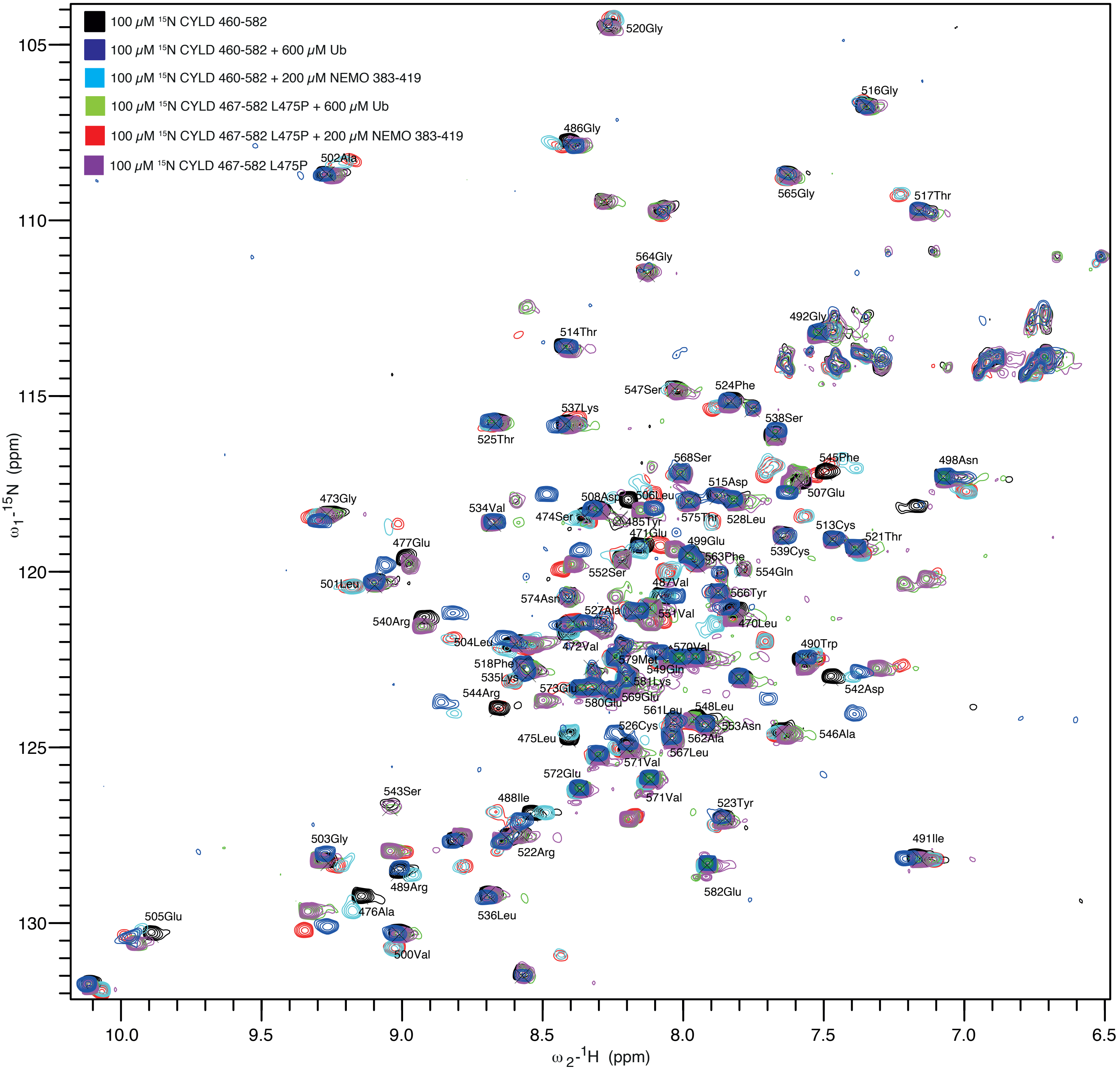

Extended Data 4. Map of chemical shift perturbations, Related to Figure 2.

BEST-TROSY spectra for ^{15}N-labelled CAP-Gly3 (black) in the presence of six-fold molar excess of Ub (dark blue) or two-fold molar excess of NEMO (light blue). The CAP-Gly3 Ub-binding mutant L475P, was recorded alone (purple) or in the presence of six-fold molar excess NEMO (red) exhibited similar chemical shift perturbations as WT CAP-Gly3. However, L475P is does not undergo any chemical shift perturbations in the presence of six-fold molar excess Ub (green).
